# Transformation of artistic style and innovative design of oriental folk patterns based on AIGC Technology—A case study of Zhuxian town new year paintings from China

**DOI:** 10.1371/journal.pone.0346020

**Published:** 2026-05-27

**Authors:** Jiangxu Zhang, Jinsong Kuang, Xiaosi Huang, Pengfei Lei, Dag Øivind Madsen, Yongqing Feng

**Affiliations:** 1 Xiamen Academy of Arts and Design, Fuzhou University, Xiamen, China; 2 School of Economics and Trade, Hunan University of Technology and Business, Changsha China; 3 School of Humanities and Arts, Xiangtan Institute of Technology, Xiangtan, China; 4 USN School of Business, University of South-Eastern Norway, Hønefoss, Norway; 5 School of Applied Economics, Guizhou University of Finance and Economics, Guiyang, Guizhou, China; Macau University of Science and Technology, MACAO

## Abstract

In response to the limited cross-domain innovation in the digitalization of traditional oriental folk art, this study takes the New Year pictures of China’s Zhuxian Town as a case. It develops a collaborative technical framework of combining the Liblib platform and a LoRA model to explore AI-generated digital re-creation of traditional art. A high-quality dataset was built through a three-stage process of image acquisition, multidimensional screening, and expert review. A three-layer keywords thesaurus was constructed through literature analysis, questionnaire surveys, and semantic clustering. Using a two-stage training strategy combining pre-training and fine-tuning, along with dynamic optimization, the model accurately captures the stylistic features of Zhuxian New Year pictures. The generated outputs integrate traditional aesthetics with modern design and are applied to cultural and graphic creative products. The results demonstrate the effectiveness of the proposed framework for preserving artistic style while enabling cross-domain innovation and offer a practical technical reference for the digital inheritance and modernization of related folk arts.

## 1. Introduction

With deep learning, GANsGans, DMS, and other technologies, Artificial Intelligence Generated Content (AIGC) has become a key tool for art design, showing significant potential in image generation, style transfer and cross-modal integration, and providing a new path for traditional art innovation [1–3]. At present, AIGC is transitioning from theory to practice, enhancing creative efficiency and facilitating the integration of traditional and modern elements [4], but the relevant applications are still in the experimental stage, especially in the field of oriental folk art patterns, which lack systematic research, and the existing achievements focus on single style generation, with insufficient cross-domain innovation [5,6]. As representatives of oriental folk art, Zhuxian Town’s Figure New Year pictures feature bright colors and profound folk customs [7], but they face an inheritance dilemma amid modernization [8]. Although there are rescue protection methods such as 3D scanning and AI modeling [9], the creative transformation driven by AIGC still needs to be deepened. Therefore, it is of great significance to conduct relevant research to promote the inheritance of traditional art and culture and the integration of modern innovation.

Based on the core requirements of the research on “digital recreation of oriental folk art,” the Liblib platform was selected to carry out LoRA model training, and its adaptability is highly consistent with the research objectives, mainly for the following three reasons: first, it can effectively support the traditional art pattern feature extraction and model fine-tuning, and adapt to the training scene of small sample data of Zhuxian Town New Year pictures; Second, the function of the platform meets the needs of cross-domain pattern design, and can realize the integration of traditional patterns and modern elements, providing practical support for exploring the path of digital recreation.

The purpose of this study is to take the New Year pictures of Zhuxian Town as the research object, extract the core features of the patterns through the Liblib platform and the LoRA model, integrate modern design elements, construct cross-domain mixed patterns, explore the AIGC digital recreation path of traditional folk art, and realize the coordinated development of cultural inheritance and modern innovation. In view of the existing problems of cross-domain innovation and the lack of systematic research in the field of oriental folk art in AIGC, this study clarifies the feasibility of the Liblib+LoRA model in the application of traditional pattern feature extraction and fusion generation, and provides an effective reference for the digital protection and innovation of similar folk art.

The rest of the paper is structured as follows. The second part presents related work on artistic style transformation based on AIGC technology. The third part analyzes the characteristics of China’s Zhuxian Town figure New Year Painting, serving as a representative example of oriental traditional folk art. The fourth part is the method and process of AIGC based on Liblib and LoRA. The fifth part is the results and analysis of the Liblib+LoRA generation model framework. The sixth part is the conclusion, which discusses implications and limitations.

## 2. Related work

Research on the transformation of artistic styles driven by AIGC technology initially centered on Western art systems, leading to three major technical paradigms: Neural Style Transfer (NST), Generative Adversarial Networks (GANs), and diffusion models. Gatys proposed Neural Style Transfer using convolutional neural networks (CNNs) to separate content and style features, achieving, for the first time, efficient transfer of Western classical styles such as Van Gogh’s “Starry Night” and Picasso’s Cubism [10]. This method minimized the weighted sum of content loss and style loss, infusing target styles while preserving image content, marking a milestone in computational art. However, NST relies on manually designed style loss functions that are ineffective at transferring complex styles (such as Abstract Expressionism) and computationally expensive. Since 2017, the rise of Generative Adversarial Networks (GANs) has propelled style transfer into an interactive phase. CycleGAN and StarGAN achieved bidirectional conversion between visual formats like photographs, oil paintings, and comics by constructing multi-domain mapping mechanisms. For example, pix2pix model based on conditional GANs, accurately converts semantic labels into realistic images, demonstrating engineering value in fields such as architectural design and product rendering [11]. However, these methods have highlighted the issue of “pattern simplification” in Oriental art, where discriminators focus solely on pixel-level authenticity, neglecting the deeper consistency of cultural semantics. Wang et al. noted that when transferring abstract aesthetic features, such as “negative space” and “texturing techniques,” from Chinese landscape paintings, GAN-generated results often suffer from limitations in adversarial losses, leading to flattened brushstroke structures [12]. After 2020, diffusion models, notably Stable Diffusion, revolutionized the generation paradigm through the “denoising diffusion-inverse process.” This model demonstrates overwhelming advantages in Western realistic painting (such as classical oil paintings) and abstract art (such as Pop Art). It supports high-resolution generation at 1024x1024, achieving semantic controllability through text prompts (e.g., “Renaissance style, delicate light and shadow”), and generating diversity far surpassing that of early GANs. The research shows that the diffusion model has greatly improved the style restoration of Monet’s light and shadow and Dali’s surrealism (based on clip image text alignment score), significantly improving the efficiency and freedom of artistic creation.

In integrating AIGC with traditional Oriental art design, Chen et al. [13] combined a Cycle GAN with characteristics of Chinese ink painting, proposing a layered algorithm for processing “gongbi” and “xieyi” styles. They achieved style transfer in ink-figure paintings by extracting features of brush density and line rhythm. Experiments showed that this method could reproduce the “five tons of ink” layering effect at 78%. However, it still relied on manual parameter tuning for expressing abstract aesthetics like “atmosphere,” failing to capture culturally specific aesthetic logics such as “negative space” and “the interplay of reality and illusion” [14]. Yang et al. conducted a digital study on Wuhu iron paintings, optimizing the generation of metal forging textures using a Hierarchical Visual Transformer (HVT) model combined with a grayscale co-occurrence matrix to quantify the spatial distribution of hammering marks, thereby increasing the texture similarity to 82% [15]. However, this method heavily depends on the parametric description of specific processes (such as hammering force and angle), making it difficult to transfer to other Oriental art forms (such as paper cutting and New Year paintings), revealing limitations in technical versatility. Mersha et al. [16] further noted that existing models face the issue of “semantic drift” when processing culturally specific content. For example, in Chinese New Year paintings, models may simplify the solemn posture of “door gods” into vivid visual symbols, overlooking their cultural connotations of warding off evil and bringing good fortune, leading to works that lack deep artistic logic. Empirical studies show that without introducing a cultural knowledge base, the diffusion model’s correct recognition rate for Oriental art symbols is only 53%, significantly lower than that for Western art scenes [16].

In summary, current research presents a pattern of “Western dominance and Oriental lag,” with the core contradiction being the compatibility between technological paradigms and cultural semantics. Although Western art styles have developed mature technical systems, research on Oriental traditional art is still in its infancy, with the main challenge being the deep modeling of cultural semantics and the precise translation of visual symbols. Western art styles (such as Realism and Abstract Expressionism) rely on quantifiable visual features (such as color brightness and brushstroke direction). In contrast, Oriental art (such as Literati Painting and Ukiyo-e) emphasizes esthetic categories that cannot be directly observed, such as “atmosphere” and “vitality.” The shallow feature extraction of existing models falls short of meeting these needs [12]. Moreover, regional style analysis is insufficient, and general models (such as Stable Diffusion) suffer from “semantic drift” when handling culturally specific content. For example, they lack targeted analysis of the unique “knife flavor” carving texture and the color system formed by natural pigments like Huaihuang and Muhong in Zhuxian Town New Year paintings.

The potential contributions of this study include the following three aspects: First, from the perspective of digital remastering, the study expands the research boundaries of innovation in traditional Oriental folk art. Existing studies often focus on the static preservation and replication of traditional New Year paintings (e.g., the transmission of intangible cultural heritage skills). However, in this study, we use low-rank fine-tuning techniques based on the LoRA model to extract the “rough lines” and “flat color overlay” characteristics of Zhuxian Town New Year paintings. By combining these with the parameterization design module of Liblib software, it achieves semantic integration of traditional elements with modern styles (such as dynamically matching the “door god holding a halberd” posture with minimalist geometric backgrounds), providing a new research path for the digital transformation of traditional New Year paintings. Second, the study enriches the research on cross-domain hybrid design application paths in intangible cultural heritage folk art. Although some previous studies have explored the application of digital technology in traditional culture, specific implementation paths have not received sufficient attention. Through the deep integration of technology and art, this study reveals the possibility of digital remastering to preserve cultural essence while promoting artistic innovation, offering theoretical support for the modern transformation of traditional folk art. Third, in terms of research methodology, this study uses a closed-loop process of “LoRA fine-tuning-Liblib interaction design-user feedback,” enabling real-time adjustments to generated results via the Liblib interface (e.g., optimizing color saturation and adjusting pattern proportions). This has the benefit of achieving human-machine collaborative innovation and providing methodological support for the digital design of complex cultural products.

The structure of the rest of the paper is as follows: The second part analyzes the characteristics of China’s Zhuxian Town figure New Year pictures as a representative example of traditional oriental folk art; the third part is the AIGC technical pattern design process based on Liblib and LoRA; the fourth part is the design and application of Liblib + LoRA generative model architecture; the fifth part is the application scene of Chinese Zhuxian Town figure New Year pictures; the sixth part is the conclusion and enlightenment.

## 3. Analysis of the characteristics of New Year paintings in China’s Zhuxian Town

The Chinese New Year picture of Zhuxian Town is a treasure of traditional oriental folk art, reflecting a strong accumulation of regional culture and rich, colorful forms of expression. It shows a distinctive regional style in terms of aesthetic implications, theme types, color application, and clothing background. In this study, we combine the historical heritage and existing works of art of Chinese Zhuxian Town’s New Year pictures, make an in-depth analysis, and summarize the main artistic characteristics of its characters.

### 3.1. Esthetic features

#### 3.1.1. Body proportions.

**Fig 1:**
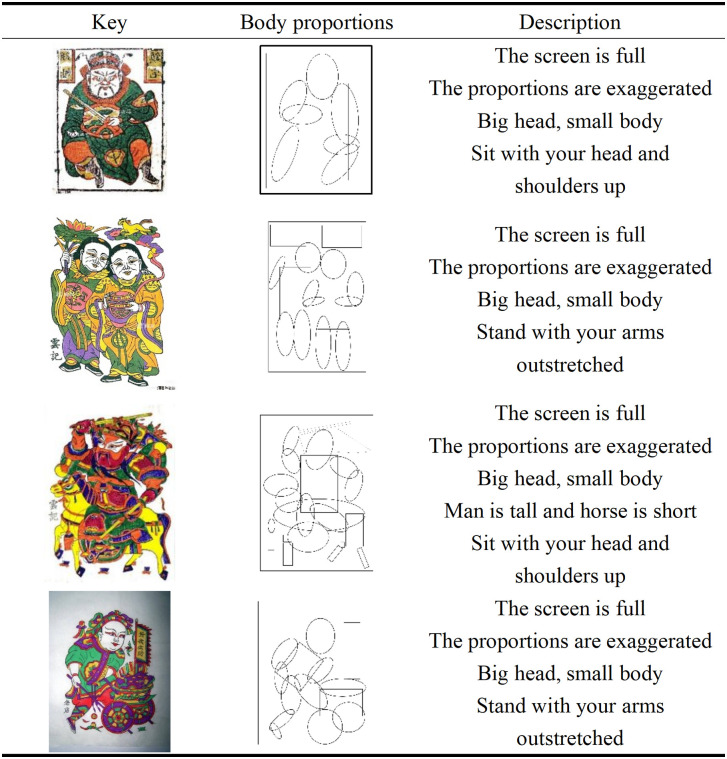
Body proportions of characters in New Year paintings in Zhuxian Town.

Zhuxian Town Figure New Year pictures often use exaggeration to shape the figure, highlighting the proportions of a large head and a small body, thereby strengthening the expression of facial emotion and conforming to the tradition of exaggeration and symbolism in oriental folk art [17]. This design not only enhances the image’s vividness and visual impact [18], but also deepens the viewer’s impression, consolidates its status as a cultural symbol, and conveys the auspicious meaning of the festival [19], becoming a distinctive identification mark of New Year pictures in folk art (see Fig 1).

#### 3.1.2. Facial features.

**Fig 2:**
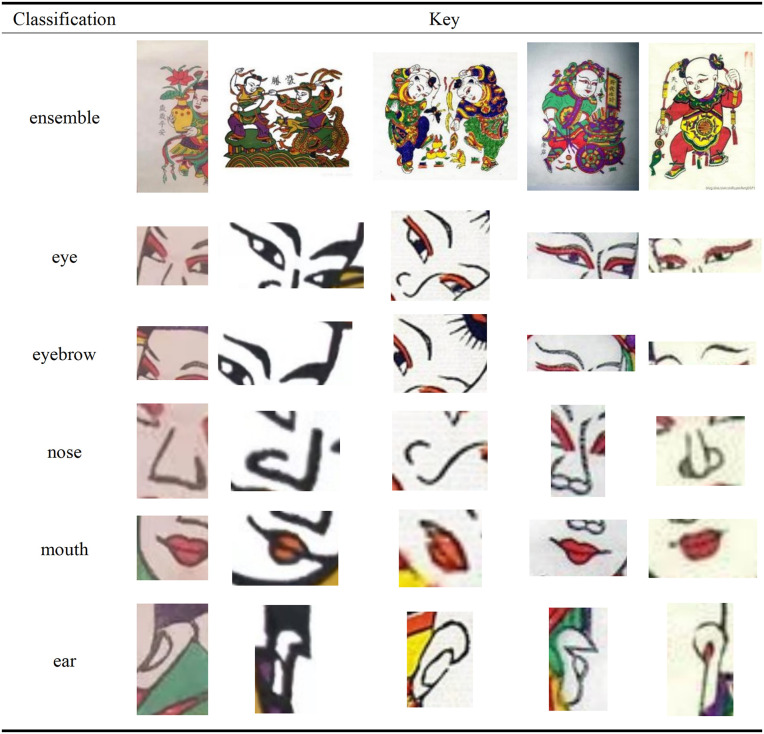
Facial features of characters in the year of Zhuxian Town.

The Zhuxianzhen New Year pictures depict the face with exaggerated symbolism, enlarging the eyes, eyebrows, and other features to highlight emotion and character, enhance visual impact, and shape a unique artistic style. Facial expressions are not only related to the identity and personality of the characters [20], but also contain a festive atmosphere, repose the yearning for a better life, and endow the works with strong drama and vividness (see Fig 2).

### 3.2. Genre features

**Fig 3:**
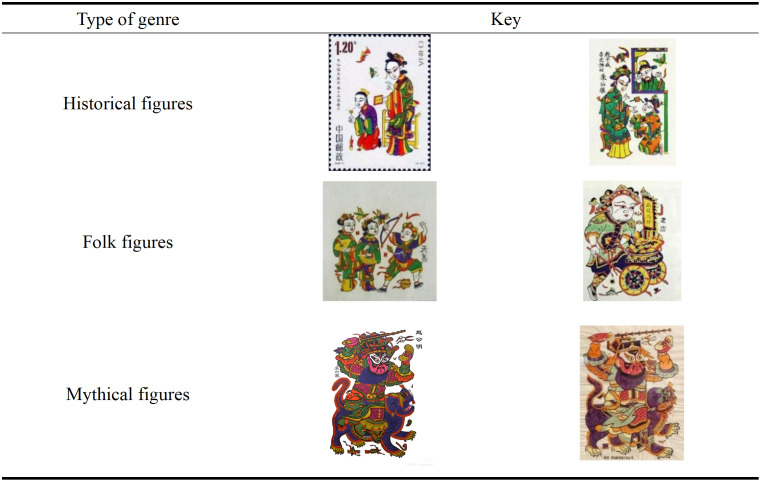
Types of New Year paintings on characters in Zhuxian Town.

Zhuxian Town’s figure New Year pictures are rich in themes, covering history, folk heroes, mythical figures, etc., highlighting the profound cultural heritage of oriental folk art [7]. Its image mostly contains auspicious symbols (such as the God of wealth, wealth and longevity), which not only embodies the yearning for happiness and longevity, but also carries the admiration for mythical and historical figures [21], both praising virtue and the guiding role of education [22], and consolidating cultural and social values through the diversity of themes (see Fig 3).

### 3.3. Color characteristics

**Fig 4:**
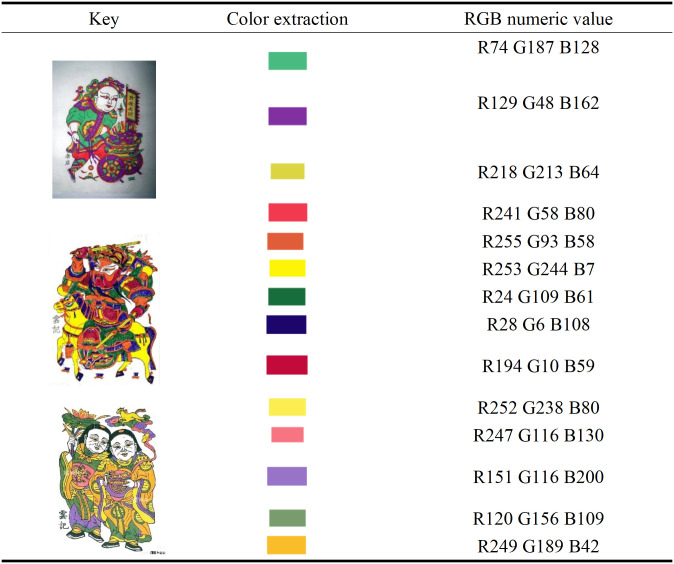
Color characteristics of character New Year paintings in Zhuxian Town.

Zhuxian Town figure New Year pictures are characterized by bright and strong color contrast (see Fig 4). The core colors include black, red, purple, green and yellow, which not only reflect the folk “five color concept,” but also convey the festival atmosphere and auspicious implication [23]. In traditional culture, red is associated with good luck, celebration and wealth [24]. Green and yellow symbolize vitality and prosperity. Bright colors not only enhance visual impact, highlight characters, but also evoke the yearning for happiness and harmony. They have both decorative functions and rich cultural symbolic significance [25].

### 3.4. Clothing and element characteristics

**Fig 5:**
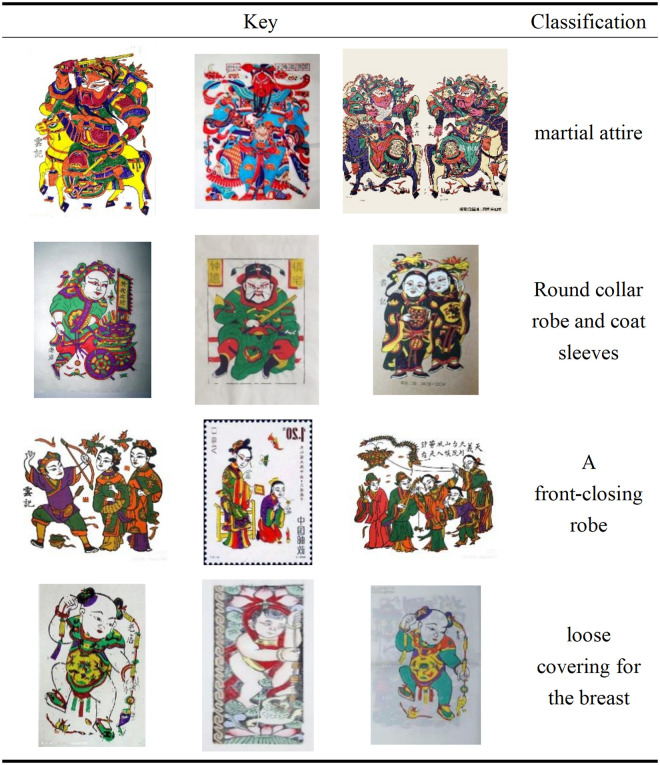
Costume of characters in Zhuxian Town.

The clothing and element design of Zhuxian Town figure New Year pictures highlight the richness of regional culture [8]. The costumes are mostly based on the Ming Dynasty official clothes or folk traditional styles (such as round neck robes and wide sleeves), which not only identify historical figures, immortals and other identities, but also convey auspicious and noble cultural symbols; auspicious clouds, flowers and other decorative elements symbolize good luck, prosperity and longevity, and enrich the deep social and cultural value of the work [26]. Its decorative and symbolic treatment also highlights the image of the characters and promotes the harmony and unity of the work (see Fig 5).

## 4. Methods and processes

### 4.1. Research methods

#### 4.1.1. Core technology selection.

We adopt the collaborative scheme of “Liblib platform+LoRA model” to enhance text-to-image generation. The Liblib platform is an advanced system that integrates deep learning and a generative adversarial network (GAN). It supports text-to-image and image-to-image conversion through a diffusion model and a confrontation neural network. This platform provides a stable, flexible, and general-purpose framework for handling complex image-generation tasks.

#### 4.1.2. Data construction methods.

Data construction primarily involves building high-quality image and keyword databases. Among them, the high-quality image library adopts the three-level method of “multi-channel collection – multi-dimensional screening – expert review” to collect the public resources, academic literature images, and network public images of the museum, and finally determines 210 effective images according to the resolution ≥ 1080p, integrity, typical style, and comprehensive theme. Through the process of “literature analysis – Questionnaire – semantic clustering,” the keyword thesaurus extracts core keywords from three dimensions of artistic features, cultural semantics, and technical parameters, and establishes an accurate mapping between text and visual features after optimization. Among them, the optimal redrawing range and key guidance coefficient of the picture are collected through expert interviews, and the key weight optimization is collected through an anonymous questionnaire survey. See S1 Appendix and S2 Appendix for the specific scheme.

#### 4.1.3. Model training and optimization methods.

To train an optimal model that generates high-quality New Year pictures, a dynamic optimization method is primarily used, involving continuous evaluation and iterative adjustment of the model’s generation performance. Specifically, we dynamically adjust the keyword weight distribution to match the aesthetics and style consistency of the generated image, and fine-tune its details, color saturation, and composition balance. Using the above dynamic optimization strategy, an optimal model that stably produces high-quality Zhuxian Town New Year picture-style images is successfully trained.

#### 4.1.4. Cross-domain fusion generation method.

The “double path generation method” is adopted to achieve deep integration between traditional artistic features and modern design elements, balancing creativity and practical transformation. In terms of the wenshengtu path, input instructions are constructed based on the keyword paradigm of “traditional core elements+modern design language,” highlighting the cultural identity and modern style of New Year pictures, and through the semantic guidance of the standardized thesaurus, innovative patterns with both cultural identification and modern aesthetic value are generated. In terms of the path of drawing from drawing, the original color of Zhuxian Town is used as the input, and the optimized LoRA is used to inject the colors, lines and cultural semantics of New Year pictures on the basis of retaining the functional structure of the works, so as to achieve creative transformation and avoid “semantic drift” and “feature distortion.”

### 4.2. Design process

Artificial intelligence content generation (AIGC) technology relies on advanced algorithms and image generation models to automatically generate creative content through large-scale dataset learning, providing a new path for cross-domain mixed pattern design, that is, integrating pattern features from different fields to produce innovative designs. This study focuses on the New Year pictures of Zhuxian Town. Based on an in-depth analysis of its historical origins, artistic style, and pattern characteristics, this study selects an appropriate theme field and carries out cross-domain mixed pattern innovation practice using AIGC technology, aiming to generate a fusion pattern inspired by the style of New Year pictures. It should be noted that the current AIGC model still has significant limitations in the detailed processing and style-accurate reproduction of traditional handicraft patterns (such as Zhuxian Town New Year pictures) [27].

This research adopts the collaborative technology scheme of “Liblib platform+LoRA model”: Liblib, as an AIGC tool integrating deep learning and generation of countermeasures networks (Gans), cooperates with countermeasures neural network to realize text-image and image-image bidirectional conversion [28], with an intuitive interface, efficient computing power and good compatibility; LoRA (low rank adaptation), as an efficient in-depth learning and fine-tuning tool, is widely used in engineering, art design and other fields. Its core advantage is to freeze the main parameters of the pre-training model, capture task-specific characteristics only by adjusting a small number of low-rank matrix parameters, and avoid the high cost and resource consumption of large-scale re-training. When the two are combined, LoRA optimizes the generation effect through the mechanism of reinforcement learning and human feedback, accurately matches the core features of Zhuxianzhen New Year pictures, such as bright colors, exaggerated shapes, and symmetrical composition, and reduces the creation threshold for non-professionals.

**Fig 6:**
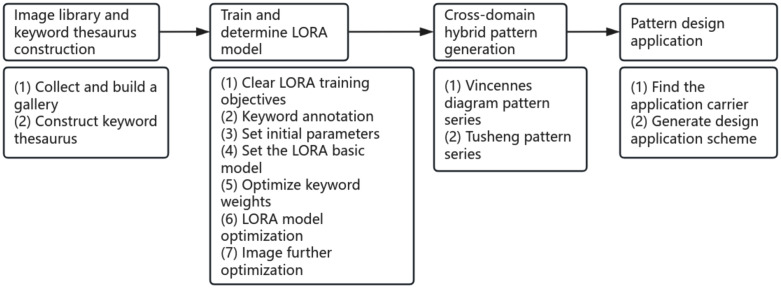
Liblib Design flow chart.

During the implementation of the study, the relevant data for Zhuxian Town New Year pictures were first screened and sorted, and a training image library containing 210 high-quality images and a three-dimensional, standardized keyword thesaurus was constructed. Then, Liblib was fine-tuned in orientation based on the LoRA model. Through two-stage training of “pre training fine tuning” and dynamic parameter optimization, it ensured that the model accurately captured the artistic characteristics of New Year pictures; Finally, cross-domain mixed patterns are generated through the two paths of Wenshengtu and Tushengtu, so as to realize the organic integration of traditional art and modern technology. This method not only retains the cultural connotation and artistic charm of Zhuxian Town New Year pictures but also promotes the innovation and inheritance of traditional crafts through cross-domain integration. The specific design process is shown in Fig 6.

### 4.3. Ethical statement

This study adheres to the Declaration of Helsinki and the ICMJE ethical guidelines. Before the study began, all participants signed a written Informed Consent Form (see S3 Appendix) and a Joint Ethical Compliance Commitment by the Authors (see S4 Appendix), acknowledging the purpose, anonymity, and privacy protection clauses; the consent forms were encrypted and kept by the researchers. Data evaluation data obtained from five experts’ signed Data Use Consent Forms (see S5 Appendix) and External Expert Statements (see S6 Appendix). A Human Participant Study Checklist (see S7 Appendix) was also completed. All electronic data was stored on an encrypted hard drive, accessible only to team members. A second anonymization review was conducted before publication, completely removing all direct and indirect personal identifiers; details are provided in the attached S8 Appendix " Data Anonymization Instructions.” The researchers pledge that the rights and privacy of participants are the primary principles throughout the entire process.

### 4.4. Disclosure of AI Use

During the preparation of this manuscript, the authors used AI software such as Kimi/ChatGPT/LIBLIB for language polishing, translation, and chart generation. The authors have reviewed and edited the AI-generated output to ensure its accuracy and applicability. These AI tools were used solely to assist in writing and did not participate in core research design, data collection, statistical analysis, or results interpretation.

## 5. Results and analysis

According to the design process, the AIGC pattern design for Zhuxian Town’s New Year paintings includes three stages: First, collect images of traditional New Year paintings from Zhuxian Town and establish a material library, converting image texts into keyword libraries; second, train and optimize LoRA to obtain the optimal LoRA model that best represents the style of Zhuxian Town’s New Year paintings. Finally, the optimal LoRA model will be used to generate cross-domain hybrid patterns for Zhuxian Town’s New Year paintings.

### 5.1. Image library and keyword library construction

Before generating the Chinese Zhuxian Town New Year paintings, a large number of images of figures from Zhuxian Town were first collected and organized. These images included traditional auspicious patterns such as door gods, Nian beasts, and the symbols of fortune, prosperity, and longevity. Each image was accompanied by a detailed text description, covering details such as the story, figure design, artistic style, and color scheme [29]. By summarizing the initial images and text descriptions, a library of images and a keyword lexicon were formed to train the AIGC technology model [30].

#### 5.1.1. Constructing a Graphic Library.

To generate high-quality images, initial images need to undergo preprocessing, including resolution normalization, noise reduction, watermark removal, and color adjustment, to ensure consistency and applicability. In this study, we collected and analyzed 300 images of Chinese New Year pictures in Zhuxian Town, which were all from official publications such as integration of Chinese woodcut New Year pictures· Zhuxian Town volume edited by fengjicai and Zhuxian Town woodcut New Year pictures edited by Zhang Jizhong [31,32]. In order to avoid copyright problems, the way of use complies with Article 24 of the copyright law of the People’s Republic of China, “appropriate citation” (see S9 Appendix), and the title, author, year of publication, and page number of the book have been fully marked in the references according to the academic citation standard. All images are only used for academic research, teaching, and non-commercial communication, and the list and source of images are disclosed in the form of supplementary materials, which are continuously subject to the supervision and correction of the copyright owner and the public.

This study adopted the Liblib AIGC generation model, in which image generation is guided by the model’s learning from initial images and textual descriptions of Zhuxian Town New Year paintings. Ensuring that the initial images faithfully represent traditional styles is crucial. Therefore, we conducted a rigorous screening of the collected images, with criteria including whether the image “matches the basic characteristics of Zhuxian Town New Year paintings (such as composition, color usage, and character modeling),” whether the image is clear, free from watermarks or noise, and whether it excludes duplicate, blurry, or structurally unclear images. Ultimately, 210 high-quality images were selected to build an image library rich in the characteristics of Zhuxian Town New Year paintings, serving as training data for the generation model. This image library will help the model accurately capture the artistic essence of Zhuxian Town New Year paintings, thereby generating images that conform to traditional styles [33].

#### 5.1.2. Constructing a keyword thesaurus.

**Fig 7:**
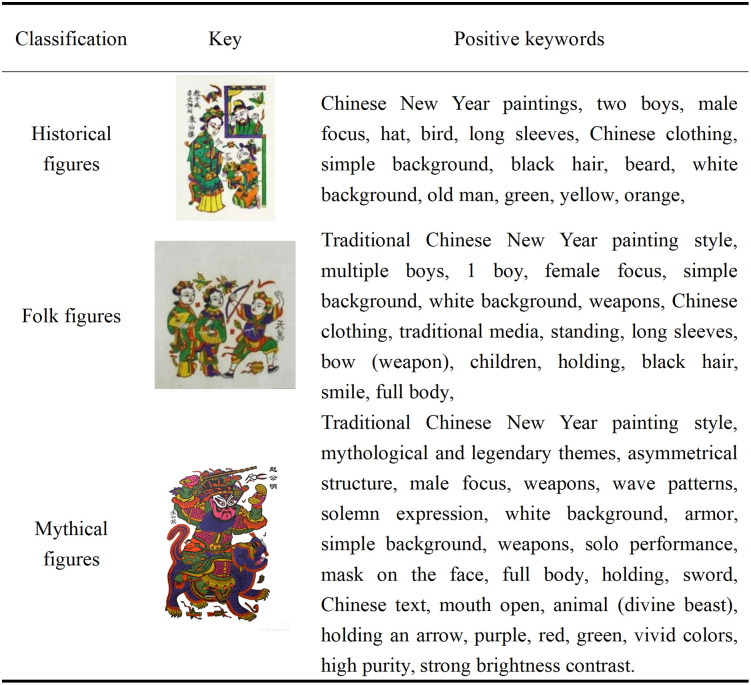
Overview of positive keywords.

**Fig 8:**
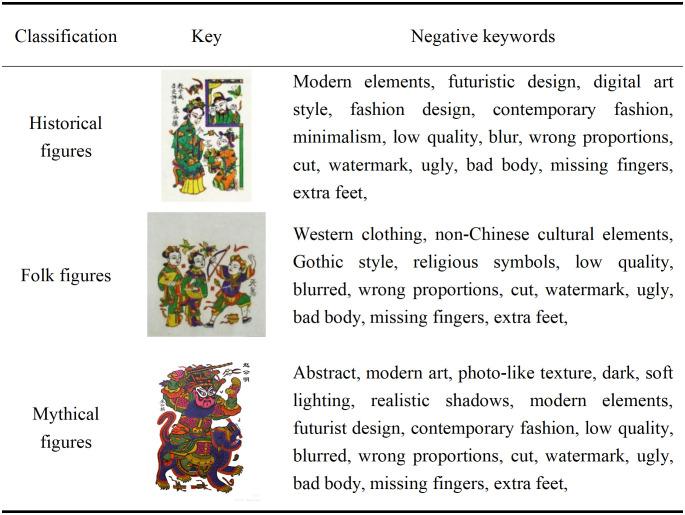
Overview of negative keywords.

Keywords, as an alternative element to textual descriptions, play a crucial role in the training process of AIGC generation models [34]. In this study, text descriptions of each image were screened and extracted into keywords to serve as input features for the Liblib and LoRA generation models, aiding the models in learning the traditional artistic style of Zhuxian Town character New Year paintings. Two methods were used for keyword extraction. First, based on theoretical research related to Zhuxian Town character New Year paintings, descriptive terms relevant to the artistic style, pattern characteristics, and cultural connotations of Zhuxian Town character New Year paintings were extracted from the screened image library, focusing on common traditional elements, composition methods, color usage, and theme features. Second, using ChatGPT-4o and other image generation tools, we generated keywords related to the style of Zhuxian Town character New Year paintings. We meticulously dissected the traditional characteristics of Zhuxian Town’s New Year paintings, paying particular attention to body proportions, facial features, themes, color usage, clothing, and backgrounds, ensuring that the generated images accurately reflect the artistic style of these paintings. Subsequently, we aggregated the keywords obtained from both methods and divided them into positive and negative keywords, constructing a comprehensive and precise keyword library (see Fig 7,8).

### 5.2. Train and determine the LoRA model

**Fig 9:**
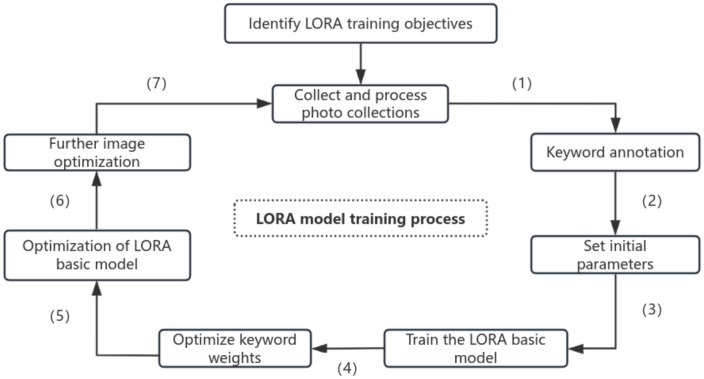
LoRA model training flow chart.

Before using the Liblib model to generate images, it is necessary to train and build a LoRA base model with the style of Zhuxian Town New Year paintings. The training process mainly includes the following four steps: First, clarify the training objectives of the LoRA model; Second, select appropriate images from a library to form an image set; Third, label the image set with keywords and set initial parameters. Through this process, the required LoRA base model can be obtained; subsequently, optimize the keyword weights, adjust the LoRA base model, and further refine the images to generate the optimal LoRA model (see Fig 9).

#### 5.2.1. Clarify the LoRA training objectives.

Clear training objectives help determine the number of image sets and the type of basic model to train. LoRA’s training objectives can be divided primarily into representational and stylistic categories. Representational goals focus on specific IP characters, figures, objects, or poses, requiring precise reproduction of their features; stylistic goals cover painting style, scenes, colors, and overall style, emphasizing the reproduction of artistic styles and thematic characteristics and offering higher generalization capabilities. Given that the research topic of this paper is New Year paintings of Zhuxian Town, the LoRA training objectives were chosen to be representational, ensuring accurate reproduction of the distinctive features of Zhuxian Town New Year paintings. Collecting and processing image sets for LoRA learning requires distinct goals and materials. An excellent image set is key to training an outstanding LoRA model. In the Zhuxian Town New Year painting library, as mentioned before, images were processed by removing watermarks, bubbles, and text, changing complex backgrounds, adjusting colors, modifying sizes, cropping, and using high-definition enlargement to form a new image set consisting of 50 images. Finally, analyzing and balancing the quantity and proportions of various image categories in the image set to avoid a high proportion of images from the same category, as this can affect model generalization.

#### 5.2.2. Keyword annotation.

Keyword annotation involves using the AI image recognition technology on the Liblib platform to automatically extract keywords from images, then manually correcting them by combining them with a constructed keyword dictionary to ensure accuracy. On the Liblib platform, commonly used automatic annotation plugins include BLIP, DeepBooru, and WD1.4. BLIP describes images through natural language and sentence structure, while DeepBooru and WD1.4 annotate images using words and phrases. Among these three, WD1.4 is preferred for its higher descriptive accuracy in this study’s image annotation [9]. Based on the results of automatic annotation, we employ four manual correction methods: modification, merging, splitting, and synonym reduction, ultimately determining the positive keywords. The image generation process relies on precise keyword guidance and image references, and the weight of keyword descriptions directly affects the quality and content of the generated images. Therefore, keyword accuracy is crucial to ensuring a high degree of consistency with the image style. We extracted descriptions of subject matter, composition, color, elements, clothing, and facial features from the keyword dictionary. We adjusted the weight coefficients of key guiding words in the LoRA model to optimize the image generation effect.

#### 5.2.3. Set initial parameters.

Set training parameters according to the final LoRA model target. The parameters include the base model, training mixed precision, total learning rate, learning rate scheduler, optimizer, enabling layered learning rate training, network size, etc. (see Table 1).

**Table 1:** Settings of some parameters in the initial LoRA model.

Parameter	Parameter values
Base model	SD1.5_TuCiv_CNMale_1.0
Train with mixed precision	bfloat16/FP16
Overall learning rate	1e-5
Learning rate scheduler	Linear
optimizer	Lion
Enable layered learning rate training	deny
Network size	102

#### 5.2.4. Train the basic LoRA model.

**Fig 10:**
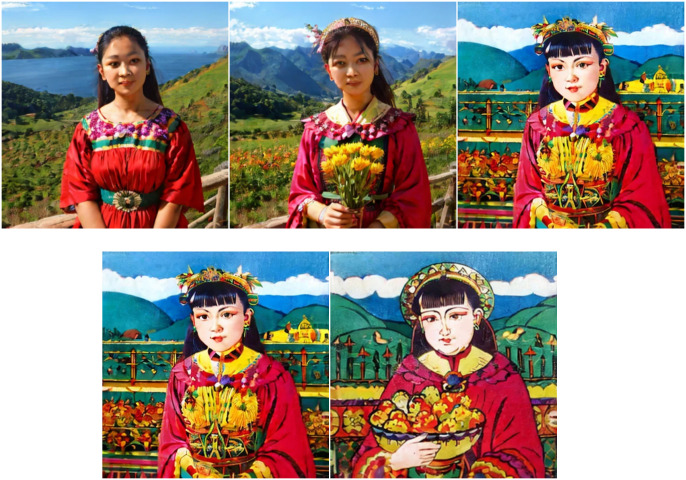
Initial results of LoRA training.

First of all, by attaching keywords to each image and setting various parameters such as 16 times of single training, 10 rounds of training, and saving a LoRA every 2 rounds, after 5 rounds of continuous learning and iterative optimization, the basic model of LoRA and the process image of 5 rounds of learning are finally obtained, as shown in Fig 10.

Secondly, the LoRA base model was tested for both text-to-image and image-to-image generation. The text-to-image method relies on input positive and negative keyword descriptions, the trained LoRA model, and basic weights. Through 20–30 iterations, an initial draft of images that match the artistic style of Zhuxian Town’s New Year paintings is generated; in the image-to-image method, with the aid of padding images, positive and negative keywords, the trained LoRA model, basic weights, and Classifier‑Free Guidance scales, 20–30 iterations are used to ultimately produce an initial draft of images that conform to the artistic style of Zhuxian Town’s New Year paintings [13].

#### 5.2.5. Optimize keyword weight.

In this link, the user survey method is used to evaluate the quality of the generated image and the degree of style matching, and to sort keywords and optimize keyword weights. This study uses an anonymous survey to evaluate the results (See S10 Appendix for the user’s informed consent form). There are 40 respondents, including 23 males and 17 females. The participants include designers, art scholars, consumers, students, and teachers. Through the respondents’ evaluation based on a set of predefined evaluation criteria, including theme, composition, color, elements, clothing, and facial features, the keywords are selected or sorted according to the image feature category, and the weight ranking of each keyword in the image generation is obtained by using the evaluation method of direct sorting and selection. The final evaluation results are shown in Table 2 (see S1 Appendix and S2 Appendix for detailed data).

**Table 2:** User evaluation results (part).

Personnel serial number	Sort	Subject matter	Composition of a picture	Colour	Element	Dress and personal adornment	Facial features
1	1	historical figures	symmetric	Red color	figure	Officers’ dress	oxeye
	3	Mythical figures	asymmetric	Yellow	animal	Folk costumes	Brow stubble
	2	Folk figures	Scenario-based	Green	A lucky symbol	Costume of the gods	The mouth is exaggerated
2	1	historical figures	symmetric	Green	A lucky symbol	Officers’ dress	Brow stubble
	3	Folk characters	asymmetric	Red color	animal	Costume of the gods	oxeye
	2	Mythical figures	Scenario-based	Yellow	figure	Folk costumes	The mouth is exaggerated
3	1	Mythical figures	symmetric	Red color	figure	Costume of the gods	oxeye
	2	Folk figures	asymmetric	Yellow	A lucky symbol	Folk costumes	The mouth is exaggerated
	3	historical figures	Scenario-based	Green	animal	Officers’ dress	Brow stubble

The keyword weight ranking is derived through statistical evaluation results (see Table 3). The keywords most characteristic of Zhuxian Town figure New Year paintings are “symmetrical structure,” “door god,” “weapon,” and “cloak.” In addition, other keyword descriptions can enhance the stylistic similarity and the detail of the image content.

**Table 3:** Keyword weight ranking.

Classification	Image features	Keyword	Sort
Subject matter	historical figures	Majestic and Brave, Armor and Battle Robes, Resolute Expression	1
	Mythical figures	Sacred and Majestic, Clouds and Cranes, Otherworldly and Serene	3
	Folk figures	Friendly and approachable, traditional clothing, radiant smile	2
Composition of a picture	symmetric	Balance, Stability, Central	1
	asymmetric	Dynamic, Guiding the Eye, Irregular	2
	Scenario-based	Environment, Narrative, Layered	3
Colour	Red color	Vibrant, Traditional, Warm	2
	Yellow	Bright, Rich, Gentle	1
	Green	Lively, Tranquil, Natural	3
Element	figure	Exaggerated, Traditional, Festive	1
	animal	Symbolic, Vivid, Auspicious	3
	A lucky symbol	Meaningful, Exquisite, Intricate	2
Dress and personal adornment	Costume of the gods	Mysterious, Exquisite, Ethereal	3
	Folk costumes	Traditional, Simple, Diverse	2
	Officers’ dress	Solemn, Refined, Noble	1
Facial features	oxeye	Exaggerated, Round, Bright	1
	The mouth is exaggerated	Big Mouth, Bold, Defined Outline	3
	Brow stubble	Dense, Heavy, Prominent	2

#### 5.2.6. LoRA model optimization.

During testing, this study used optimized keywords and selected padding images that matched the artistic style of Zhuxian Town’s New Year paintings to ensure the generated images accurately reflected the characteristics of traditional patterns. If the generated image already matches the style of Zhuxian Town’s New Year paintings, there is no need to further optimize the LoRA model; it can be considered the optimal model. However, in most cases, the generated results still require multiple rounds of testing and optimization to achieve optimal results [35]. Therefore, even if the initial image aligns with the style of Zhuxian Town’s New Year paintings, parameters such as the training base model, optimizer, network size, and sample resolution must be repeatedly adjusted until the generated image accurately reproduces the traditional artistic style of Zhuxian Town’s New Year paintings.

Therefore, the final model is determined through the three steps of “iterative training – Thinking evaluation – optimal decision” and four rounds of iteration of the LoRA model. First, in the iterative training, four rounds of LoRA model training are adopted to gradually optimize, and four key parameters (basic model, prompt word system, network structure, training intensity) are fine-tuned based on the results of the previous round in each round. The goal is to improve the generation ability of “Zhuxianzhen New Year painting style.” Secondly, the four-dimensional evaluation method is used to qualitatively evaluate the images generated by each round of the LoRA model. The standard requirements include four aspects: ① style compliance: check the four core features of “rough lines, strong color contrast, full composition and solemn image of door god”; ② Image quality: check hand deformity, facial blur and structural dislocation; ③ Prompt response: measure the accuracy of positive style guidance and negative quality constraints; ④ Training stability: take the stability of the loss curve as the index. Finally, the image generated by the fourth round of the Rola model is superior to those of the first three rounds in color, composition, and style, and shows no significant defects or loss of instability, so it is determined to be the optimal model (see S11 Appendix for detailed parameters). In the optimal model, when the following parameter values are set, the optimal LoRA model that matches the Chinese New Year pictures of Zhuxian Town figures can be obtained (see Table 4).

**Table 4:** Parameter setting of LoRA optimal model.

parameter	Parameter values
base model	Sichuan Mianzhu New Year painting _2024NewYear
Number of single training sessions	16
Number of training rounds	9
batch size	2
Train the hybrid precision	fp16
Sample resolution	512x512
Random seed count	1000000001
sample mode	DPM++ 2M Karras
call-word	DPM++ 2M Karras
Negative prompts	(worst quality, low quality:1.4),(depth of field, blurry:1.2),(greyscale, monochrome:1.1),3D face, cropped, lowres, text,(nsfw:1.3),(worst quality:2),(low quality:2),(normal quality:2), normal quality, ((grayscale)), skin spots, acnes, skin blemishes, age spot, ((ugly:1.331),(duplicate:1.331),(morbid:1.21),(mutilated:1.21),(tranny:1.331), mutated hands, ((poorly drawn hands:1.5), blurry, bad anatomy:1.21),(bad proportions:1.331), extra limbs, disfigured:1.331),(missing arms:1.331),(extra legs:1.331),(fused fingers:1.61051),(too many fingers:1.61051),(unclear eyes:1.331), lowers, bad hands, missing fingers, extra digit, bad hands, missing fingers, extra arms and legs)
Save a LoRA every N rounds	2
LoRA preserves accuracy	fp16
Total learning rate	1e-4
Unet learning rate	0.0001
Text encoder learning rate	0.00001
Learning rate scheduler	cosine_with_restarts
optimizer	Adam8bit
Restart count	1
Network size	128
Network Alpha	64
Keep n tokens	64
Maximum token length	75
Noise offset	75
Random seed count	−1
clip skip	1

**Fig 11:**
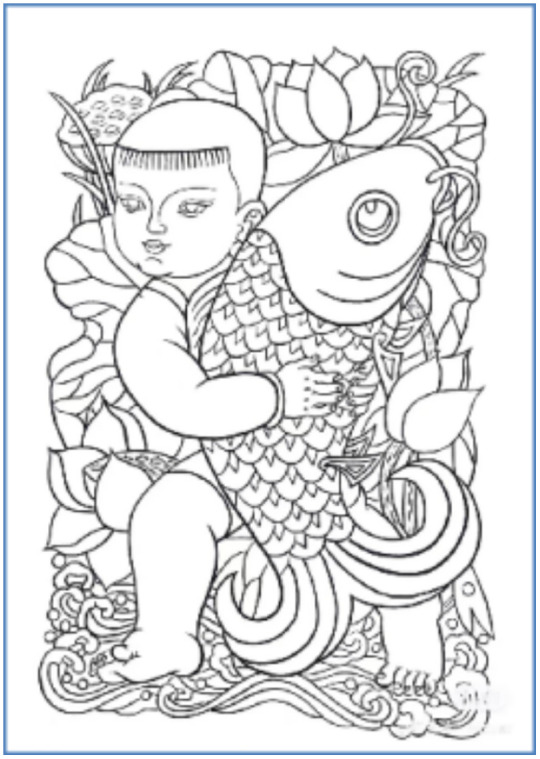
Background.

**Fig 12:**
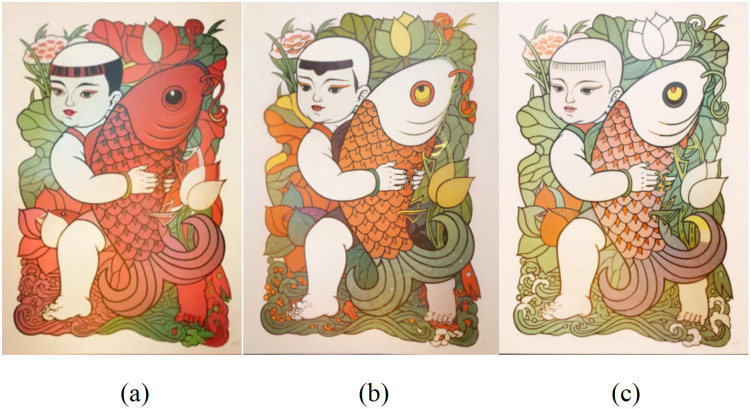
Image generation types of the optimal LoRA model.

Based on the LoRA optimal model obtained from the above optimization, the new Zhuxian Town figure New Year pictures were generated in the picture generation task, using the pad map and corresponding style keywords as inputs (as shown in Fig 11,12, detailed generation parameters can be found in S12 Appendix). In terms of visual effect, the generated image reflects the following significant features. To sum up, the results of the mapping match the typical artistic style of Zhuxian Town New Year pictures in terms of color expression, compositional structure, and style characteristics, indicating that the trained LoRA model has strong style transfer and content generation capabilities in practical applications.

**Fig 13:**
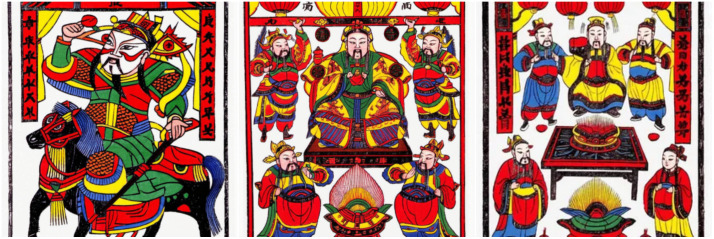
Pictures generated based on the Liblib platform.

In addition, in order to more intuitively compare the pictures generated by the LoRA optimal model with the style of the New Year pictures of Zhuxian Town figures, a group of New Year pictures of Zhuxian Town figures (Fig 13) was generated as the control group without using the LoRA optimal model and using the Liblib platform alone. It can be intuitively observed that the pictures generated solely on the Liblib platform and departing from the LoRA optimal model, with the characteristics of New Year pictures of Zhuxian Town figures, are always unsatisfactory compared with the real New Year pictures of Zhuxian Town figures.

**Fig 14:**
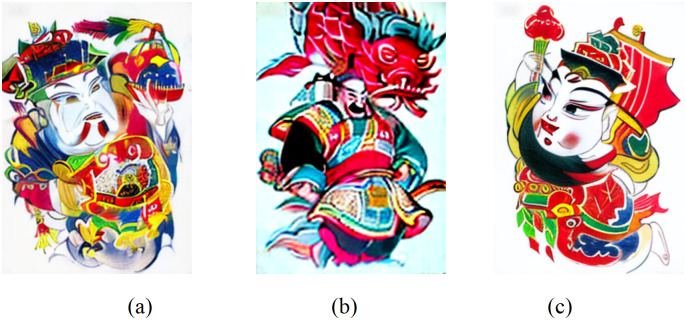
Image generated based on SDXL-LoRA model.

Finally, in order to more intuitively verify the superiority of the LoRA model in this study, we selected an existing mainstream LoRA model as a comparison, that is, the universal LoRA model based on stable diffusion XL (SDXL-LoRA). The two groups of models use the same prompt words (“door god of Zhuxian Town, symmetrical composition, red series, exaggerated face”) and random seeds (seed = 3008506526) to generate images, and the results are shown in Fig 14.

This study summarizes the comparative analysis of the images generated by the LoRA optimal model and SDXL-LoRA model (see Table 5). Obviously, the pictures generated by this research model are superior to the existing LoRA model in terms of lines, colors and character modeling: the lines show rough woodcut texture, the contrast model is either thin or soft, the colors strictly follow the traditional four-color system, the contrast model is either messy or light, the character modeling accurately presents the characteristics of “big head, small body, exaggerated face,” and the contrast model is either maladjusted or realistic. To sum up, this research model more accurately restores the core artistic features of Zhuxian Town New Year pictures.

**Table 5:** Comparative analysis of LoRA optimal model and SDXL-LoRA model.

Model	Figure number	Style characteristics	Main issues
LoRA optimal model	Fig 12 (a)–(c)	Rough lines, saturated colors, exaggerated figures, full and symmetrical composition	No obvious defects, highly consistent with the characteristics of Zhuxian Town New Year pictures
SDXL-LoRA	Fig 14 (a)–(c)	The color is disordered, the lines are broken, and the charm of traditional New Year pictures is lacking	Serious style drift, failed to capture the core features of Zhuxian Town New Year pictures

#### 5.2.7. Image generation parameter optimization.

**Fig 15:**
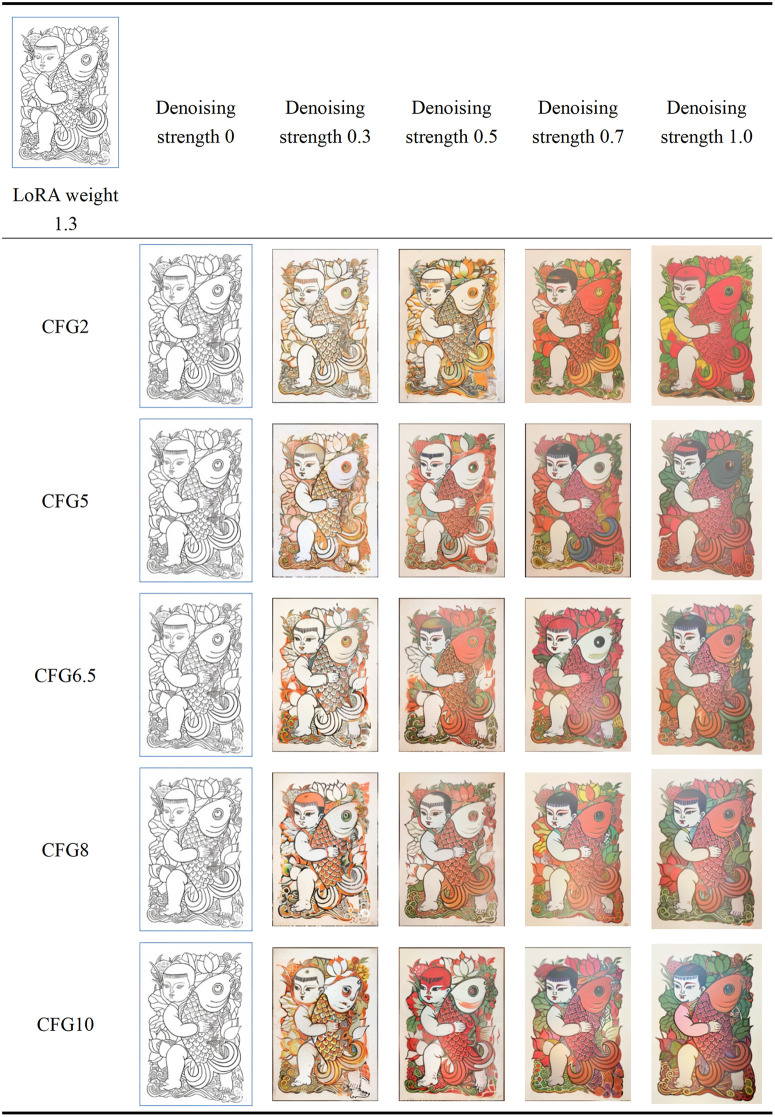
Parameter adjustment effect (1).

**Fig 16:**
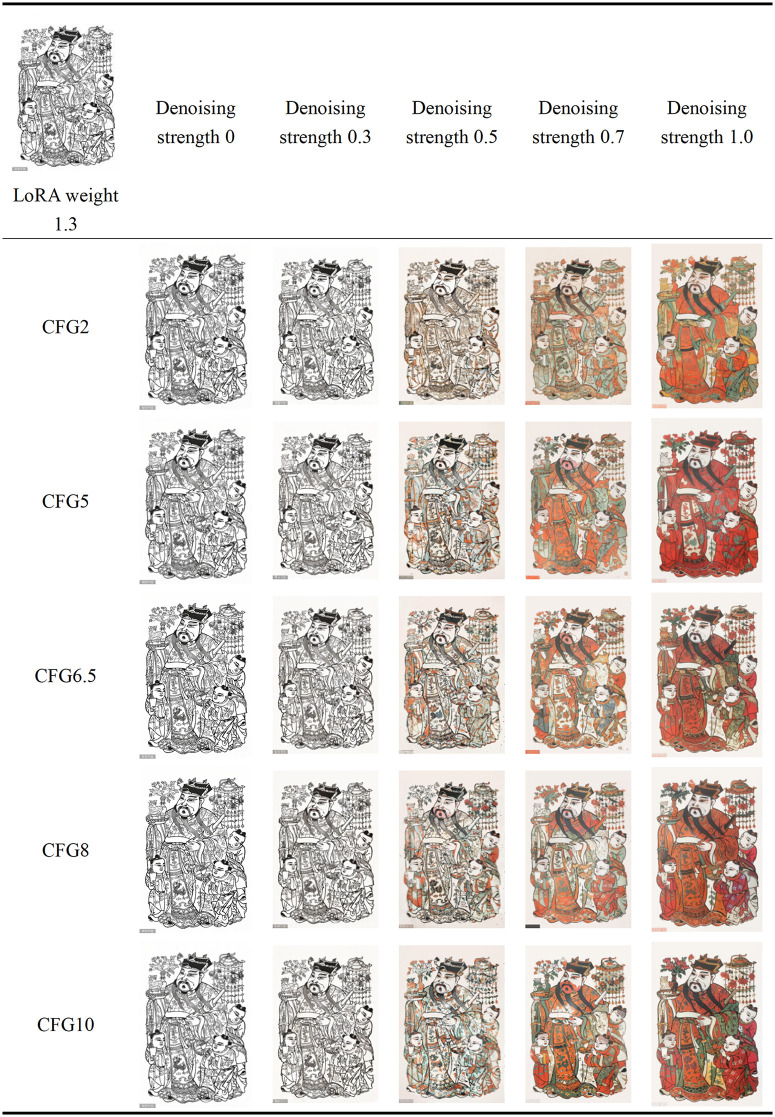
Parameter adjustment effect (2).

After optimizing the LoRA model to better align the generated patterns with the style of Zhuxian Town’s New Year paintings, further adjustments to the repainting amplitude and Classifier-Free Guidance (CFG) scale are needed during the image-to-image generation process. The repainting amplitude refers to the degree of adjustment and modification applied to the base image when generating an image. A higher repainting amplitude parameter means a greater degree of modification to the base image. Specifically, 0 does not change the base image; values below 0.3 will slightly modify the base image, values above 0.7 will significantly alter the base image, and 1 will result in an entirely different image from the base image. The Classifier-Free Guidance scale measures the impact of keywords on image style, ranging from 0 to 30. The higher the weight, the closer the image is to the keyword description. This study combines text and base images, adjusting the repainting amplitude and CFG values to ensure that the generated images remain consistent with the original base images while maintaining their basic characteristics. Control Net technology is used for effective control. Additionally, the optimal LoRA model weight is set to 1.3. To optimize the generated images, two base images were selected, resulting in 120 images, from which the 50 most representative images were selected for display (see Fig 15,16, detailed generation parameters can be found in S12 Appendix), ensuring they best align with the artistic style of Zhuxian Town’s New Year paintings.

Subsequently, we invited five experts to subjectively evaluate the images generated from the above two tables (see Table 6) to determine the optimal repainting amplitude and Classifier-Free Guidance (CFG) scale (See S13 Appendix for the recruitment process).

**Table 6:** Expert evaluation results.

Expert serial number	Most preference for text	The feature map with the strongest inclination	Most preferred fidelity of puppet style features	Mix content optimum	Hybrid style optimum
1	Denoising strength 0 CFG10	Denoising strength 1 CFG2	Denoising strength 1 CFG2	Denoising strength 0.5 CFG6.5	Denoising strength 0.7 CFG6.5
2	Denoising strength 0 CFG10	Denoising strength 1 CFG5	Denoising strength 1 CFG2	Denoising strength 0.5 CFG6.5	Denoising strength 1 CFG6.5
3	Denoising strength 0 CFG8	Denoising strength 0.7 CFG2	Denoising strength 1 CFG2	Denoising strength 0.5 CFG6.5	Denoising strength 1 CFG8
4	Denoising strength 0.3 CFG8	Denoising strength 1 CFG2	Denoising strength 1 CFG5	Denoising strength 0.5 CFG8	Denoising strength 0.7 CFG8
5	Denoising strength 0 CFG10	Denoising strength 1 CFG2	Denoising strength 0.7 CFG2	Denoising strength 0.5 CFG8	Denoising strength 1 CFG8

The specific recruitment process for experts is as follows:

① Expert Recruitment and Screening Procedure: To ensure the authority and impartiality of the evaluation, we collaborated with the China Arts and Crafts Association to carefully recruit a panel of five experts. The recruitment process adhered to strict inclusion and exclusion criteria. Inclusion criteria (which must be met simultaneously) included: at least ten years of professional experience in the relevant field, experience in judging national-level New Year painting exhibitions, and no commercial interests in AIGC technology. Exclusion criteria (if any of these criteria were met, the candidate would not be eligible) included: participation in the development of AI painting tools within the past three years; academic collaboration with members of the research team; and age over 70 years old. A dual-track system of “organizational recommendation followed by qualification review” was used for preliminary screening, resulting in a shortlist of candidate experts. Supporting materials were then rigorously reviewed against the aforementioned criteria. The final list of five experts was confirmed upon the project leader’s approval.
② Ethical Compliance: A formal invitation package was sent to the selected experts, including: an informed consent form, an authorization form, an external expert statement, a summary of the study objectives in both Chinese and English, and an explanation of the evaluation criteria (50 AIGC-generated images with different redrawing magnitudes and Classifier Free Guidance scales). All experts provided written informed consent prior to participation, and they were given time to review all materials and familiarize themselves with the evaluation framework.
③ Evaluation Execution: The expert evaluation phase proceeded as follows: Each expert conducted a subjective evaluation of the generated image set. Upon completion, the research team collected and organized the evaluation results for subsequent analysis.

The stylistic tendencies of generated images were quantified using a five-dimensional evaluation system. ① The most preference for text means the degree of fit between the image content and the text description (1 = complete deviation, 7 = highly consistent); ② The feature map with the strongest inclination refers to the structural similarity of the generated result to the original pad map (1 = significant distortion, 7 = perfect inheritance); ③ Most preferred fidelity of puppet style feature shows the continuation of the artistic style of the pad map (1 = style fracture, 7 = complete inheritance); ④ The most suitable mixed content expresses the coordination of text elements and pad map content (1 = conflict separation, 7 = organic integration); ⑤ The most suitable mixed style means the balance level of traditional style and modern design (1 = Unbalanced anomie, 7 = innovation fusion). Five domain experts independently scored each generated image based on the above dimensions (see S14 Appendix and S15 Appendix for the original data source), to sort and analyze the scoring data of the five dimensions, resulting in Fig 16.

The evaluation results show that when the repainting amplitude coefficient is 0, the image style mainly depends on the pad image’s content; as the repainting amplitude coefficient increases, the image style gradually deviates from the pad image, approaching the keyword description, with colors tending towards the red spectrum. In terms of mixing degree and style representation, when the repainting amplitude is 0.5, the generated image style most closely resembles the traditional characteristics of Zhuxian Town New Year paintings; when the repainting amplitude is between 0.5 and 1, the generated image style is most ideal, capable of retaining traditional elements while moderately incorporating modern design sensibilities. When the CFG value is 2, the content and outline of the generated image are more similar to those of the pad image, demonstrating a high degree of image similarity; when the CFG value is 10, the generated image leans more toward textual description, with a fuller composition, brighter colors, and richer details.

### 5.3. Statistical test analysis

To verify the influence of redrawing amplitude and CFG parameters on the quality of Zhuxianzhen New Year pictures generated by AIGC and to ensure the reliability of the evaluation data, this study conducted statistical tests and analyses. The data type is the continuity score (1–7 points) for five evaluation dimensions, assessed by five experts, including two core independent variables: redrawing amplitude (0, 0.3, 0.5, 0.7, 1.0) and CFG parameters (2, 5, 6.5, 8, 10). The analysis process includes an inter-rater reliability test, a main-effect parameter test, a post-comparison, and an effect calculation.

#### 5.3.1. Results of inter-rater reliability analysis.

The interrater reliability was assessed using Kendall’s W and ICC (2, K) to ensure the scores were reliable. Kendall’s w coefficient measures the consistency between raters, while ICC (2, K) evaluates the consistency of raters’ scores on the same work. The results are as follows:

As shown in Table 7 and Table 8, Kendall’s w ≥ 0.76 and P < 0.001 for all dimensions, indicating that the consistency of expert scores is significantly higher than the random level. The intra-group correlation coefficient (ICC (2, K)) for the overall score was 0.84, the standard error was 0.031, the 95% confidence interval was [0.75, 0.91], and the p-value was less than 0.001, indicating excellent reliability of the score data.

**Table 7:** Kendall’s w coefficient.

Evaluation Dimension	Number of Ratings	Kendall’s W	Standard Error	χ² Value	df	p-value	95% Confidence Interval
Most preference for text (Content Relevance)	250	0.78	0.042	93.60	4	< 0.001	[0.65, 0.89]
The feature map with the strongest inclination (Structural Similarity)	250	0.81	0.038	100.25	4	< 0.001	[0.69, 0.91]
Most preferred fidelity of puppet style feature (Style Consistency)	250	0.83	0.036	104.63	4	< 0.001	[0.72, 0.92]
Most Suitable Mixed Content (Harmony)	250	0.76	0.045	90.00	4	< 0.001	[0.63, 0.88]
Most Suitable Mixed Style (Balance)	250	0.79	0.041	95.88	4	< 0.001	[0.67, 0.90]

**Table 8:** ICC coefficient.

Reliability Type	ICC Value	Standard Error	95% Confidence Interval	p-value	Reliability Level
Two-Way Random Effects Model	0.84	0.031	[0.75, 0.91]	< 0.001	Excellent

#### 5.3.2. Main effects of parameters and post-hoc comparison results.

The Friedman test was used to analyze the effect of redrawing amplitude and CFG parameters on the score. The Friedman test is a nonparametric test used to evaluate the median difference between two or more related samples. After the comparison, the Holm correction method was used to determine the optimal parameter range via pairwise comparisons. The effect quantity calculation reports the R value and its 95% confidence interval to assess the parameter’s influence on the score.

The Friedman test was used to evaluate the influence of different redrawing amplitudes and CFG parameters on the image generation quality of Zhuxian Town figure New Year pictures. As shown in Tables 9,10, all evaluation dimensions showed significant differences across parameter settings (P < 0.001), with effect sizes (r) ranging from moderate to strong. Specifically, the effect size r for the overall score in Table 9 is 0.79, and that in Table 10 is 0.74, indicating strong effects. This shows that the denoising strength and CFG parameters significantly affect the style consistency and detail reduction of the generated image.

**Table 10:** Friedman test of CFG parameters.

Evaluation Dimension	χ² Value	df	p-value	Effect Size r	95% Confidence Interval
Most preference for text (Content Relevance)	26.34	4	< 0.001	0.32	[0.20, 0.43]
The feature map with the strongest inclination (Structural Similarity)	29.87	4	< 0.001	0.35	[0.23, 0.46]
Most preferred fidelity of puppet style feature (Style Consistency)	27.51	4	< 0.001	0.33	[0.21, 0.44]
Most Suitable Mixed Content (Harmony)	31.24	4	< 0.001	0.36	[0.24, 0.47]
Most Suitable Mixed Style (Balance)	34.62	4	< 0.001	0.38	[0.26, 0.49]
Overall Rating	98.72	4	< 0.001	0.74	[0.65, 0.81]

**Table 9:** Friedman test for redrawing amplitude.

Evaluation Dimension	χ² Value	df	p-value	Effect Size r	95% Confidence Interval
Most preference for text (Content Relevance)	28.76	4	< 0.001	0.34	[0.22, 0.45]
The feature map with the strongest inclination (Structural Similarity)	32.18	4	< 0.001	0.36	[0.24, 0.47]
Most preferred fidelity of puppet style feature (Style Consistency)	30.52	4	< 0.001	0.35	[0.23, 0.46]
Most Suitable Mixed Content (Harmony)	35.89	4	< 0.001	0.39	[0.27, 0.50]
Most Suitable Mixed Style (Balance)	33.47	4	< 0.001	0.37	[0.25, 0.48]
Overall Rating	112.36	4	< 0.001	0.79	[0.71, 0.85]

The Holm correction results are shown in Tables 11,12. For the redrawing amplitude, there is no significant difference between the scores of 0.5 and 0.7, but both are significantly higher than 0 (original image) and 1.0 (complete redrawing). For CFG parameters, there was no significant difference between 6.5 and 8, but these two values were significantly higher than 2 (the lowest parameter value) and 10 (the highest parameter value). In conclusion, adjusting the redrawing amplitude and CFG parameters in most cases significantly impacts the overall score, indicating that these parameters play an important regulatory role in the score results. However, changes in specific intervals (such as redrawing the range of 0.5–0.7, CFG parameter of 6.5–8) did not cause significant changes in scores. This suggests that, in practical applications, when fine-tuning parameters, if they fall within these intervals, they may not yield statistically significant differences. This provides a reference range for parameter optimization, simplifying the parameter adjustment process while ensuring effectiveness.

**Table 11:** Holm correction of redrawn amplitude.

Redrawing Amplitude Comparison	Mean Difference of Overall Ratings	Standard Error	z-value	Adjusted p-value
0 vs 0.5	−1.87	0.21	−8.90	< 0.001
0 vs 0.7	−1.93	0.23	−8.39	< 0.001
0.3 vs 0.5	−0.92	0.18	−5.11	< 0.001
0.3 vs 0.7	−0.98	0.20	−4.90	< 0.001
0.5 vs 1.0	1.05	0.19	5.53	< 0.001
0.7 vs 1.0	1.11	0.22	5.05	< 0.001
0.5 vs 0.7	−0.06	0.15	−0.40	0.690

**Table 12:** Holm correction of CFG parameters.

CFG Parameter Comparison	Mean Difference of Overall Ratings	Standard Error	z-value	Adjusted p-value
2 vs 6.5	−1.52	0.19	−7.99	< 0.001
2 vs 8	−1.61	0.21	−7.67	< 0.001
5 vs 6.5	−0.83	0.17	−4.88	< 0.001
5 vs 8	−0.92	0.18	−5.11	< 0.001
6.5 vs 10	0.76	0.16	4.75	< 0.001
8 vs 10	0.85	0.18	4.72	< 0.001
6.5 vs 8	−0.09	0.14	−0.64	0.523

#### 5.3.3. Validation results of optimal parameter combination.

It can be seen from Table 13 that the redrawing range of 0.5–0.7 and the CFG parameter of 6.5–8 are the optimal parameter range, and there is no significant difference in the score within this range (p > 0.05), which is significantly higher than other combinations (p < 0.001). Under this parameter combination, the generated image performs best in “mixed content coordination” and “traditional modern style balance,” which meets the style inheritance and innovation needs of Zhuxian Town New Year pictures. These results show that AIGC technology can generate works that balance traditional artistic style and modern aesthetics through precise parameter adjustments, offering a new possibility for artistic creation.

**Table 13:** Verification of optimal parameter combination.

Redrawing Amplitude	CFG Parameter	Mean ± Standard Deviation of Overall Ratings	Ranking
0.5	6.5	6.2 ± 0.5	1
0.7	8	6.3 ± 0.4	2
0.5	8	6.1 ± 0.6	3
0.7	6.5	6.0 ± 0.5	4
0.3	8	5.8 ± 0.7	5

### 5.4. Cross-domain hybrid pattern generation

**Fig 17:**
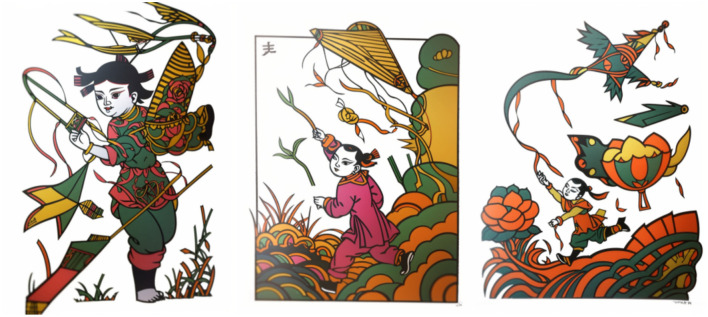
Wen Shengtu pattern series II.

Determining the optimal LoRA model and its best hyper-parameter mix furnishes a verifiable roadmap for digitising Zhuxian Town New-Year prints. The approach preserves traditional traits—auspicious meanings and vibrant colours [36]—while weaving folk symbols together with contemporary motifs [37], and opens a pathway to global reach and commercial viability. (a), (b) and (c) in Fig 17 respectively show the wenshengtu patterns generated by combining traditional symbols such as kites. To preserve the auspicious connotations, bright colors, and vivid characters of the Chinese Zhuxian Town figure New Year pictures, these patterns integrate the folk element of the kite, forming an innovative design with a sense of the times and distinct characteristics. This innovation not only enables the digital inheritance of Chinese New Year pictures in Zhuxian Town, but also promotes the innovative development and international dissemination of traditional culture. During the generation process, we paid special attention to the cultural symbols and artistic features of the Chinese New Year pictures in Zhuxian Town. By adding the element of the kite, the pattern not only retained the traditional cultural connotation but also gained new vitality in the transformation of modern digital technology, so that it can better meet the contemporary aesthetic and social needs.

**Fig 18:**
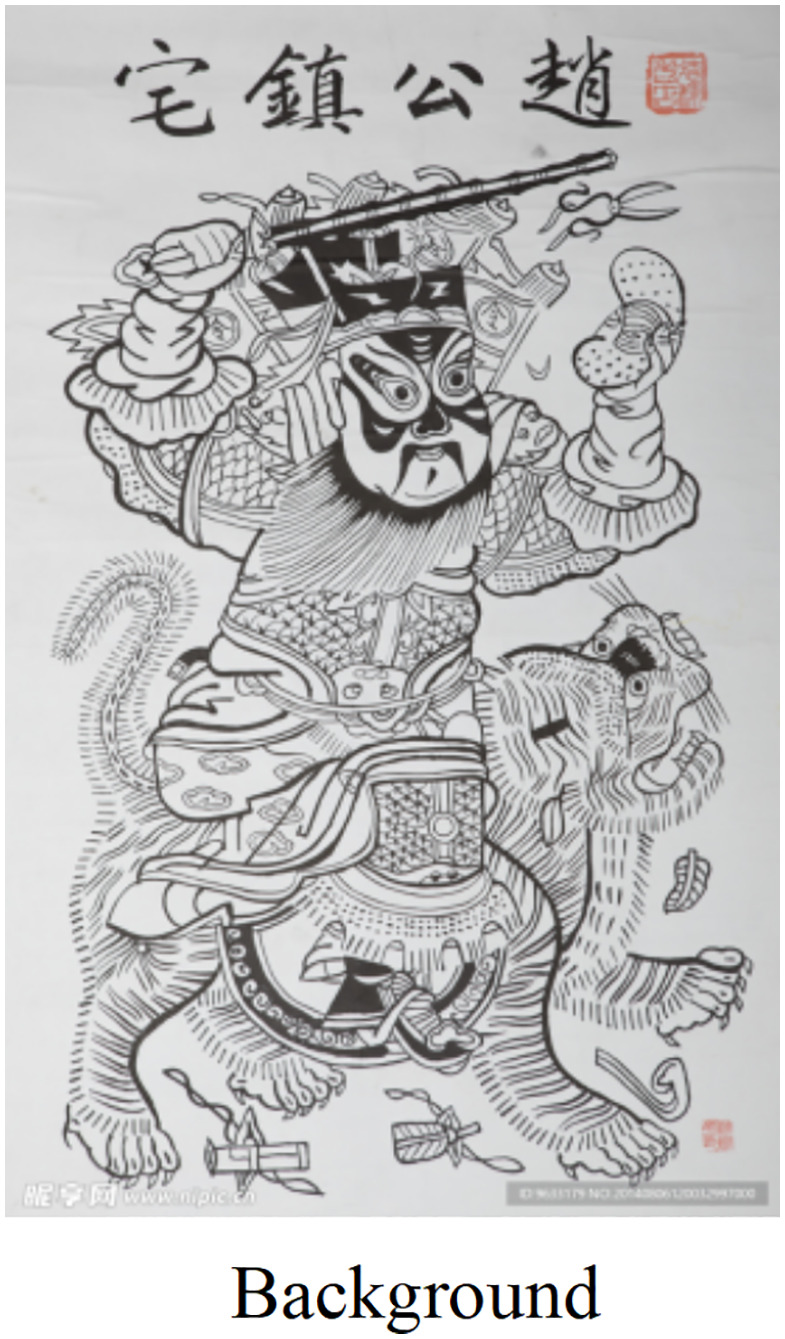
Pad chart.

**Fig 19:**
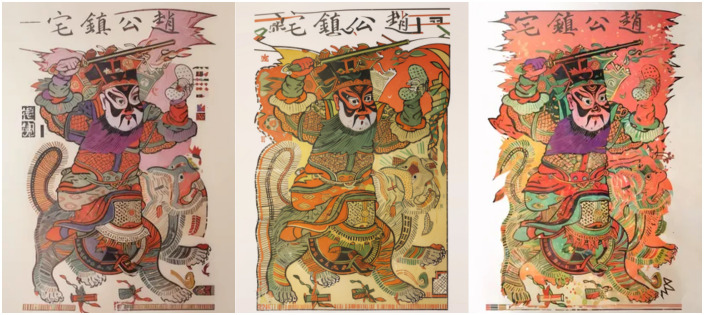
Figure pattern series 3.

Fig 18,19 (a), (b), and (c) show the Tusheng picture pattern combined with traditional auspicious symbols such as “fortune, longevity” and “five blessings at the door.” Based on retaining the Chinese New Year pictures of Zhuxian Town figures, these patterns also add symbolic elements such as the Chinese Zodiac pattern, the dragon horse spirit and the rich bamboo, forming an innovative design with a sense of the times and characteristics. This design not only retains the traditional cultural connotation of the New Year but also gains new vitality through the transformation of modern digital technology, enabling it to better meet contemporary aesthetic and social needs. The combination of digital technology and traditional folk art provides a new path for recreating traditional art [38], and as globalization and digitization deepen, it enhances its global communication capabilities [39].

Cross-domain hybrid pattern generation not only meets the needs of modern design but also provides a feasible path for commercial applications [40]. The combination of traditional cultural symbols and modern design sense can be widely used in home furnishing, fashion, brand packaging, and other fields, with huge market potential. This integration of traditional art and modern design is not only artistic recreation but also promotes the exploration of cultural marketization. It not only inherits the Chinese New Year picture of Zhuxian Town in the digital age, but also enhances its global communication power, providing new ideas for the innovation and commercialization of traditional folk art [41].

### 5.5. Application scenarios

As a typical representative of Chinese traditional folk art, Zhuxian Town figure New Year pictures provide unique cultural resources for the development of local tourism commodities with their historical and cultural connotations and folk symbolic significance [42]. Under the background of cultural self-confidence improvement and cultural tourism integration, the function of tourism commodities has shifted from simple goods consumption to cultural experience and emotional carrier, and the elements of New Year pictures can just strengthen tourists’ sense of identity and belonging to local culture through traditional aesthetics, folk stories, and beautiful meanings.

The construction of this cultural identity depends on systematic design transformation. The designer needs to deeply integrate auspicious symbols, folk characters, and traditional stories into the New Year pictures, along with local customs, historical background, and natural landscape, to create a commodity form that retains traditional genes and conforms to modern aesthetics. For example, the door god image can be refined into simplified graphical symbols, which can be applied to daily necessities such as pillows, canvas bags, mugs, etc., so that the traditional patterns can be transformed from the single function of plane decoration into a touchable and portable cultural media [8].

**Fig 20:**
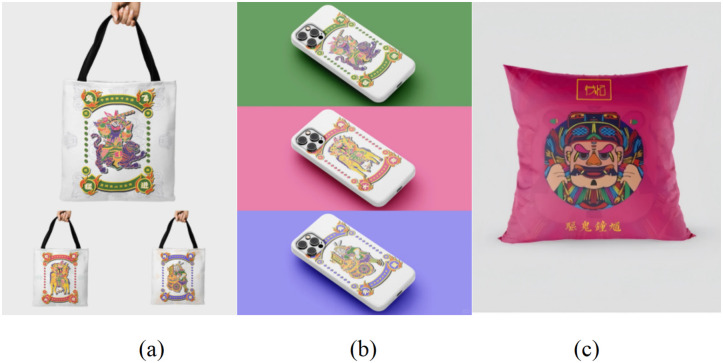
Product design scheme.

The effectiveness of this transformation strategy has been verified in the cultural and creative industries. For the cross-domain application of New Year pictures, some research systems have tested its feasibility in home, digital accessories, office supplies, and other categories, and confirmed that through theme customization and symbol redesign, the local representativeness and commemorative value of products can be significantly enhanced. As shown in Fig 20, the use of New Year pictures in the design of mobile phone cases and canvas bags not only expands the expressive dimension of traditional art but also provides a large-scale business model for the inheritance of cultural activities.

## 6. Conclusion and implications

### 6.1. Conclusion

Based on the collaborative technical framework of “Lblib platform+LoRA model,” this study systematically explores the cross-domain innovation path of traditional art digitization through the closed-loop process of “data preparation – model training – pattern generation – Application landing.” The core conclusions are as follows: The Liblib platform + LoRA model, trained in a two-stage manner, captures the core features of New Year pictures, such as rough lines and flat overprint, and the generated patterns retain cultural recognition and conform to modern aesthetics, verifying the feasibility of AIGC in the digital transformation of traditional art. The 210 images+keywords database established in this study can serve as a reference for other folk art digitization projects. The patterns have been applied to cultural and creative products and tourism commodities. Preliminary feedback indicates they are conducive to cultural heritage, but the long-term effects need to be continuously monitored.

### 6.2. Discussion

The “LIBLIB+LoRA” framework constructed in this study applies this technology to the narrative New Year pictures of Zhuxian Town for the first time, which significantly expands the application boundary of AIGC. Different from the research on geometric patterns conducted by Wang et al. (2025) [43], this framework realizes the controllable generation of complex cultural semantics through a closed-loop process. Compared with Zhang’s research on painted pottery restoration (2024) [44], this framework converts tacit knowledge (e.g., the “big head and small body” shape) into feature vectors, and solves the problem of “cultural symbol generation distortion” pointed out by Dai et al. (2024) [45].

In terms of the training mechanism, the “pre-training and fine-tuning” two-stage method adopted in this study is designed with dynamic optimization according to the characteristics of New Year pictures, i.e., “coexistence of stylization and flexibility,” which is different from the single-stage fine-tuning method proposed by Hou et al. (2024) [46]. This method achieves better performance in few-shot learning, using only 210 images, and its data scale is smaller than that of other relevant studies. In terms of generation controllability, multi-dimensional conditional control is realized through dual-path generation and platform encapsulation, establishing an adjustable space between cultural identification and modern aesthetics. The risk of “style homogeneity” warned by Fareed et al. (2024) [47] is avoided through expert review.

In practice, this research realizes the closed loop of technology value. First, the experience of inheritors (e.g., color matching formulas) is transformed into reproducible digital features, and the digital transformation framework proposed by Huang et al. (2024) [48] is operationalized, providing a reproducible technological paradigm. Second, this work addresses the ethical initiatives of Ghaith (2024) [49], balances technical efficiency and humanistic value, and avoids “Cultural Aphasia.”

### 6.3. Research limitations

Despite the significant achievements of AIGC technology in image generation, it still faces several challenges and limitations. Existing generative models struggle to capture the details and complexity of traditional art styles, especially in highly refined pattern designs, often requiring multiple iterations and extensive model training to achieve the desired results. Furthermore, in terms of cultural characteristics, AIGC may transform the traditional Zhuxianzhen New Year paintings, which are based on woodblock printing and handcrafted textures, into pixel-level smooth vectors, weakening the sensory authenticity of intangible cultural heritage techniques such as “knife marks” and “paper textures.” Confusing the labels “house-guarding tiger” and “wealth-bringing tiger” during model training may lead to a misinterpretation of the auspicious meanings of cultural symbols. AI-driven mass design may crowd out the customized market of local handicraft workshops, triggering community anxiety about technology replacing manual labor.

## Supporting information

S1 Appendix Research on the core artistic features of Zhuxian Town Figure New Year pictures. (DOCX)

S2 Appendix Survey questionnaire on the preference of core artistic features of Zhuxian Town Figure New Year pictures. (DOCX)

S3 Appendix User informed consent. (DOCX)

S4 Appendix Author’s joint ethical compliance commitment. (DOCX)

S5 Appendix Data Use Consent Forms. (PDF)

S6 Appendix External expert statement. (PDF)

S7 Appendix PLOSOne_Human_Subjects_Research_Checklist. (DOCX)

S8 Appendix Data Anonymization Instructions. (DOCX)

S9 Appendix Contents of Article 24 of the copyright law. (DOCX)

S10 Appendix User informed consent form. (PDF)

S11 Appendix Training Model Evolution. (DOCX)

S12 Appendix Supplementary Information. (DOC)

S13 Appendix Recruitment process for specialists. (DOCX)

S14 Appendix Experts evaluate the raw data. (PDF)

S15 Appendix Summary of expert evaluation data. (XLSX)
