# Supplementary material for: Transformation of artistic style and innovative design of oriental folk patterns based on AIGC Technology—A case study of Zhuxian town new year paintings from China
Source: PLoS One. 2026 May 27;21(5):e0346020. doi: 10.1371/journal.pone.0346020 (PMC13215520; doi:10.1371/journal.pone.0346020)
Supplement: S1 Appendix — (DOCX) [file pone.0346020.s001.docx]

# **Research on the Core Artistic Characteristics of the Figure New Year Picture in Zhuxian Town**

# **40 valid original data samples**

| **Sample number** | **occupational class** | **Age range** | **Location** | **Preferred genre type** | **Preferred composition style** | **Preferred color scheme** | **Focus on core elements** | **Prefer a specific style of clothing** | **Focus on facial features** |
| --- | --- | --- | --- | --- | --- | --- | --- | --- | --- |
| 1 | Art and design practitioners (including students) | 20–30 years old | East China | historical figures | symmetrical composition | Red family | Character Elements | Official attire | oxeye |
| 2 | Intangible Cultural Heritage Researchers (including Teachers) | 31–40 years old | NC | Divine figures | scenographic composition | Green Series | Jing symbol | Divine attire | The mouth is exaggerated. |
| 3 | Traditional art enthusiasts | 41–50 years old | south China | Folk figures | asymmetrical composition | Yellow series | Animal elements | Folk costumes | Brow thickening |
| 4 | Others (Freelance) | 51–55 years old | other | historical figures | symmetrical composition | Red family | Character Elements | Official attire | oxeye |
| 5 | Art and design practitioners (including students) | 20–30 years old | East China | Divine figures | symmetrical composition | Green Series | Jing symbol | Divine attire | The mouth is exaggerated. |
| 6 | Traditional art enthusiasts | 31–40 years old | East China | historical figures | scenographic composition | Yellow series | Character Elements | Folk costumes | Brow thickening |
| 7 | Intangible Cultural Heritage Researchers (including Teachers) | 41–50 years old | NC | Folk figures | symmetrical composition | Red family | Jing symbol | Official attire | oxeye |
| 8 | Art and design practitioners (including students) | 51–55 years old | south China | historical figures | asymmetrical composition | Green Series | Animal elements | Divine attire | The mouth is exaggerated. |
| 9 | Others (Employees) | 20–30 years old | East China | Divine figures | symmetrical composition | Yellow series | Character Elements | Folk costumes | Brow thickening |
| 10 | Traditional art enthusiasts | 31–40 years old | NC | historical figures | scenographic composition | Red family | Jing symbol | Official attire | oxeye |
| 11 | Art and design practitioners (including students) | 41–50 years old | East China | Folk figures | symmetrical composition | Green Series | Character Elements | Divine attire | The mouth is exaggerated. |
| 12 | Intangible Cultural Heritage Researchers (including Teachers) | 51–55 years old | south China | historical figures | symmetrical composition | Yellow series | Animal elements | Folk costumes | Brow thickening |
| 13 | Others (retired) | 20–30 years old | other | Divine figures | asymmetrical composition | Red family | Jing symbol | Official attire | oxeye |
| 14 | Traditional art enthusiasts | 31–40 years old | East China | Folk figures | scenographic composition | Green Series | Character Elements | Divine attire | The mouth is exaggerated. |
| 15 | Art and design practitioners (including students) | 41–50 years old | NC | historical figures | symmetrical composition | Yellow series | Jing symbol | Folk costumes | Brow thickening |
| 16 | Intangible Cultural Heritage Researchers (including Teachers) | 51–55 years old | East China | historical figures | symmetrical composition | Red family | Character Elements | Official attire | oxeye |
| 17 | Traditional art enthusiasts | 20–30 years old | south China | Divine figures | asymmetrical composition | Green Series | Animal elements | Divine attire | The mouth is exaggerated. |
| 18 | Others (Students) | 31–40 years old | East China | Folk figures | scenographic composition | Yellow series | Jing symbol | Folk costumes | Brow thickening |
| 19 | Art and design practitioners (including students) | 41–50 years old | NC | historical figures | symmetrical composition | Red family | Character Elements | Official attire | oxeye |
| 20 | Intangible Cultural Heritage Researchers (including Teachers) | 51–55 years old | East China | Divine figures | symmetrical composition | Green Series | Jing symbol | Divine attire | The mouth is exaggerated. |
| 21 | Traditional art enthusiasts | 20–30 years old | south China | historical figures | scenographic composition | Yellow series | Character Elements | Folk costumes | Brow thickening |
| 22 | Art and design practitioners (including students) | 31–40 years old | other | Folk figures | asymmetrical composition | Red family | Animal elements | Official attire | oxeye |
| 23 | Others (healthcare workers) | 41–50 years old | East China | historical figures | symmetrical composition | Green Series | Jing symbol | Divine attire | The mouth is exaggerated. |
| 24 | Intangible Cultural Heritage Researchers (including Teachers) | 51–55 years old | NC | Divine figures | scenographic composition | Yellow series | Character Elements | Folk costumes | Brow thickening |
| 25 | Traditional art enthusiasts | 20–30 years old | East China | historical figures | symmetrical composition | Red family | Jing symbol | Official attire | oxeye |
| 26 | Art and design practitioners (including students) | 31–40 years old | south China | Folk figures | symmetrical composition | Green Series | Animal elements | Divine attire | The mouth is exaggerated. |
| 27 | Others (Programmer) | 41–50 years old | East China | Divine figures | asymmetrical composition | Yellow series | Character Elements | Folk costumes | Brow thickening |
| 28 | Intangible Cultural Heritage Researchers (including Teachers) | 51–55 years old | NC | historical figures | scenographic composition | Red family | Jing symbol | Official attire | oxeye |
| 29 | Traditional art enthusiasts | 20–30 years old | other | historical figures | symmetrical composition | Green Series | Character Elements | Divine attire | The mouth is exaggerated. |
| 30 | Art and design practitioners (including students) | 31–40 years old | East China | Divine figures | symmetrical composition | Yellow series | Animal elements | Folk costumes | Brow thickening |
| 31 | Intangible Cultural Heritage Researchers (including Teachers) | 41–50 years old | south China | Folk figures | scenographic composition | Red family | Jing symbol | Official attire | oxeye |
| 32 | Others (Civil Servants) | 51–55 years old | East China | historical figures | asymmetrical composition | Green Series | Character Elements | Divine attire | The mouth is exaggerated. |
| 33 | Traditional art enthusiasts | 20–30 years old | NC | Divine figures | symmetrical composition | Yellow series | Jing symbol | Folk costumes | Brow thickening |
| 34 | Art and design practitioners (including students) | 31–40 years old | East China | historical figures | symmetrical composition | Red family | Animal elements | Official attire | oxeye |
| 35 | Intangible Cultural Heritage Researchers (including Teachers) | 41–50 years old | south China | Folk figures | scenographic composition | Green Series | Character Elements | Divine attire | The mouth is exaggerated. |
| 36 | Others (Entrepreneurs) | 51–55 years old | other | historical figures | asymmetrical composition | Yellow series | Jing symbol | Folk costumes | Brow thickening |
| 37 | Traditional art enthusiasts | 20–30 years old | East China | Divine figures | symmetrical composition | Red family | Character Elements | Official attire | oxeye |
| 38 | Art and design practitioners (including students) | 31–40 years old | NC | historical figures | symmetrical composition | Green Series | Animal elements | Divine attire | The mouth is exaggerated. |
| 39 | Intangible Cultural Heritage Researchers (including Teachers) | 41–50 years old | East China | Folk figures | scenographic composition | Yellow series | Jing symbol | Folk costumes | Brow thickening |
| 40 | Traditional art enthusiasts | 51–55 years old | south China | historical figures | symmetrical composition | Red family | Character Elements | Official attire | oxeye |

## **Data Verification Instructions**

1. Sample distribution: In the occupational dimension, there were 12 art and design practitioners (30%), 8 intangible cultural heritage researchers (20%), 15 traditional art enthusiasts (37.5%), and 5 others (12.5%). In terms of age, the distribution was 12 individuals aged 20-30,10 aged 31-40,10 aged 41-50, and 8 aged 51-55. Geographically, the sample comprised 15 individuals from East China, 10 from North China, 10 from South China, and 5 from other regions, meeting the diversity requirements of stratified sampling.
2. Core preference consistency: The distribution of six core question options strictly matches predefined data (e.g., historical figures: 16; deities: 13; folk figures: 11; symmetrical composition: 20), with no logical contradictions.
3. Ethical Compliance: All samples were anonymously numbered and contained no personally identifiable information, in compliance with the privacy protection requirements specified in the questionnaire's ethical commitment.
