# Supplementary material for: Transformation of artistic style and innovative design of oriental folk patterns based on AIGC Technology—A case study of Zhuxian town new year paintings from China
Source: PLoS One. 2026 May 27;21(5):e0346020. doi: 10.1371/journal.pone.0346020 (PMC13215520; doi:10.1371/journal.pone.0346020)
Supplement: S2 Appendix — (DOCX) [file pone.0346020.s002.docx]

# **A Questionnaire on the Preference of the Core Artistic Features of the Figure New Year Picture in Zhuxian Town**

## **Questionnaire Instructions**

1. The purpose of this research is to understand the public's preference for the core artistic features of the figure New Year paintings in Zhuxian Town, and to provide academic references for the digital inheritance and innovation of traditional folk art.
2. Ethical Commitment: The questionnaire was collected anonymously, and all data were used solely for academic research without commercial purposes. The raw data were stored in encrypted form, and personal information was strictly confidential. Only aggregated statistical results were presented.
3. Participation rights: You may join voluntarily and exit at any time during the form-filling process. Your data will not be saved after exiting. After completing the form, you can reserve an email to receive a summary of the survey results. If you have any objections to the use of your data, you may request deletion.
4. Time required: Approximately 3 minutes. Choose based on your actual experience. All questions are single-choice.

## **1. Informed Consent**

□ I have read and understood the above instructions, voluntarily participate in this survey, and consent to the use of questionnaire data for academic research.

## **II. Basic Information (Anonymously collected, for sample representativeness analysis only)**

1. Your profession:

□ Art and design practitioners (including students)

□ Researchers of intangible cultural heritage (including teachers and scholars)

□ Enthusiasts of traditional arts

□ Others (Please indicate: ________)

1. Your age:

□ 20–30 years old

□ 31–40 years old

□ 41–50 years old

□ 51–55 years old

1. Your location:

□ North China (Beijing, Tianjin, Hebei, Shanxi, Inner Mongolia)

□ East China (Shanghai, Jiangsu, Zhejiang, Anhui, Fujian, Jiangxi, Shandong)

□ South China (Guangdong, Guangxi, Hainan)

□ Others (Please indicate: ________)

## **III. Core Preference Survey**

1. Which type of theme do you prefer in Zhuxian Town New Year paintings?

□ Historical figures (such as generals, ministers, and celebrities)

□ Immortal figures (such as the God of Wealth, the God of Happiness, and the God of Longevity)

□ Folk figures (such as farmers, craftsmen)

1. Which composition style of Zhuxian Town New Year paintings do you prefer?

□ Symmetrical composition (balance between left and right or top and bottom)

□ Scene-based composition (including complete story background)

□ Asymmetric composition (clear distinction between primary and secondary elements, staggered layout)

1. Which color system of Zhuxian Town New Year paintings do you prefer?

□ Red color scheme (with red as the main color)

□ Green palette (with green as the main color)

□ Yellow tones (with yellow as the dominant color)

1. Which core elements in Zhuxian Town New Year paintings do you pay more attention to?

□ Character elements (face, posture)

□ Auspicious symbols (such as clouds and flowers)

□ Animal elements (such as auspicious beasts, poultry)

1. Which style of clothing do you prefer in the figures of Zhuxian Town New Year paintings?

□ Official attire (such as official robes and jade belts)

□ Immortal attire (such as the rosy-hued robe and feathered garments)

□ Folk clothing (such as short shirts, plain clothes)

1. Which exaggerated features of Zhu Xianzhen's facial expressions do you pay more attention to?

□ Large eyes (protruding and enlarged)

□ Exaggerated lip movements (overly shaped lips)

□ Thick eyebrows (bold and prominent)

## **IV. Supplementary Recommendations (Optional)**

Do you have any suggestions on the artistic features or digital innovation of Zhuxian Town New Year paintings?

________ (Please fill in. Leave blank if no suggestion is available)

## **Epilogue**

Thank you for participating. To get a summary of the survey results, please leave your email (optional): ________

We will send you a summary of the results within one month after the survey. Thank you again for your support in preserving traditional folk arts!
