# Supplementary material for: Transformation of artistic style and innovative design of oriental folk patterns based on AIGC Technology—A case study of Zhuxian town new year paintings from China
Source: PLoS One. 2026 May 27;21(5):e0346020. doi: 10.1371/journal.pone.0346020 (PMC13215520; doi:10.1371/journal.pone.0346020)
Supplement: S3 Appendix — (DOCX) [file pone.0346020.s003.docx]

# **User informed consent**

## **Research Title: Transformation of Artistic Style and Innovative Design of Oriental Folk Patterns Based on AIGC Technology — A Case Study of Zhuxian Town New Year Paintings from China**

### **I. Research Description**

Dear participants:

Hello! We are the Zhu Xian Town New Year Picture Design Research Team, currently conducting research on "Artistic Style Transformation Based on AIGC Technology and Innovative Design of Oriental Folk Patterns—Taking China Zhu Xian Town Figure New Year Pictures as an Example." This study aims to optimize the application of AIGC technology in the digital innovation of folk art by collecting your subjective ratings on the design of related patterns. The research process only requires you to complete a set of rating tasks (about 5-8 minutes), with no additional burden.

### **II. Your Rights and Related Explanations**

1. Voluntary Participation: Your participation is entirely voluntary, and you may withdraw from the study at any stage without conditions. Upon withdrawal, all submitted data will be immediately deleted by us, and no adverse effects will be incurred to you.
2. Data Usage Statement: All scoring data provided by you will be used solely for academic analysis and publication of this study. The data will undergo full anonymization (marked only as 'Participant XX' without any personal identification information), with no commercial purposes and will not be disclosed to third parties.
3. Privacy Commitment: This study does not collect any personally identifiable information such as your name, age, contact details, or ID number. The data is stored on an AES-256 encrypted cloud server, with access restricted to only two core researchers.
4. Data retention period: For 3 years after publication, we will permanently delete the original scoring data and retain only the aggregated statistical results for academic archiving.

### **III. Informed Consent Confirmation**

I have carefully read all the above instructions, fully understand the purpose, procedures, data usage, and my rights in this study, voluntarily participate in this research, and agree to the research team using my scoring data as described above.

□ I have read and understood all the content, agree to participate in this study, and consent to the use of the interview content for academic publication after all personally identifiable information has been removed. (Checking this box means I have signed and consented).

□ Disagree to participate in this study (marked to discontinue scoring)
