# Supplementary material for: Transformation of artistic style and innovative design of oriental folk patterns based on AIGC Technology—A case study of Zhuxian town new year paintings from China
Source: PLoS One. 2026 May 27;21(5):e0346020. doi: 10.1371/journal.pone.0346020 (PMC13215520; doi:10.1371/journal.pone.0346020)

# **Author's Joint Ethics and Compliance Commitment Letter**

As the author of the study titled "A Study on the Style Evaluation of Innovative Design of Zhu Xian Town New Year Picture Patterns under AIGC Technology" (hereinafter referred to as "this study"), I hereby solemnly undertake the following ethical compliance commitments:

1. This study involved the collection of scoring data from human subjects (5 experts and 40 general participants). The research process strictly adhered to international and domestic academic ethical standards, including the Declaration of Helsinki and the Ethical Review of Research Involving Human Subjects, and complied with the ethical requirements for human subject research set by PLOS ONE.
2. All participants were fully informed of the study objectives, data usage, participation rights, and privacy protection measures. Informed consent was obtained through either paper signing or online confirmation, with the consent process being authentic, complete, and valid, without any concealment or inducement.
3. This study did not collect any personally identifiable information (PII). Data storage, usage, and retention complied with privacy protection regulations and were exclusively used for academic analysis and publication of this study, with no commercial purposes or other non-compliant usage scenarios.
4. As the institution where this study is conducted has not yet established a dedicated IRB approval mechanism, multiple measures such as "data encryption storage, ethical self-inspection, and full-process anonymization" have been implemented to ensure ethical compliance. All relevant supporting documents (scanned copies of informed consent forms, signing records, data security statements, etc.) are authentic and verifiable.
5. In the event of any disputes arising from ethical compliance issues in this study (including but not limited to privacy breaches or infringement of participants' rights), all authors shall be jointly and severally liable, and the journal and related institutions shall not be held responsible.

We hereby commit.


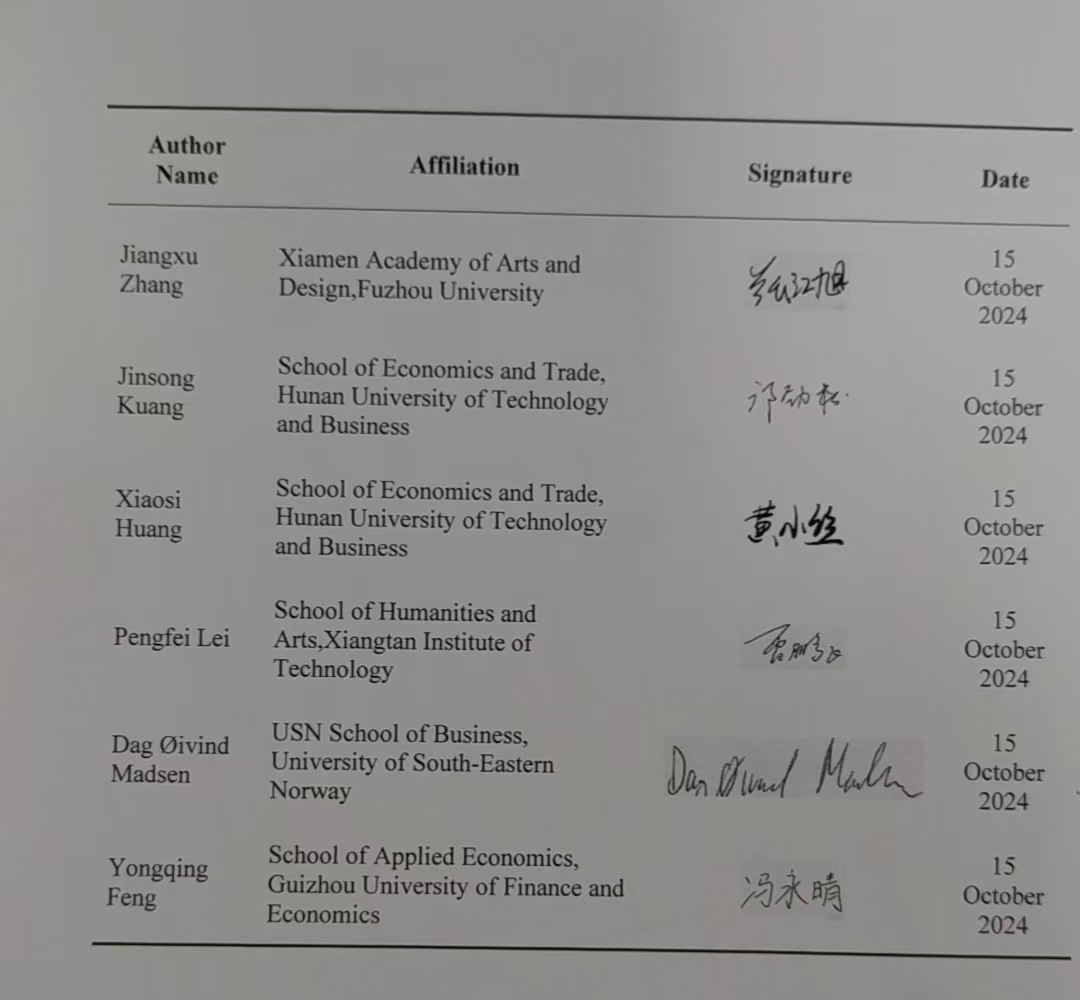

Supplement: S4 Appendix — (DOCX) [file pone.0346020.s004.docx]
