# Supplementary material for: Transformation of artistic style and innovative design of oriental folk patterns based on AIGC Technology—A case study of Zhuxian town new year paintings from China
Source: PLoS One. 2026 May 27;21(5):e0346020. doi: 10.1371/journal.pone.0346020 (PMC13215520; doi:10.1371/journal.pone.0346020)
Supplement: S5 Appendix — (PDF) [file pone.0346020.s005.pdf]

Chang'e Kuang, Associate Professor in Art Design, School of Art, Hunan University of Science and Technology
Xiaotong Li, Lecturer in Art Design, Hunan University Of Technology and Business
Ziwei Luo, Lecturer in Film and Television Animation, Xiangtan Institute of Technology
Qinglei Du, Lecturer in Fine Arts, Xiangtan Institute of Technology
Zizheng Liu, Lecturer in Art Design, Xiangtan Institute of Technology

CONSENT LETTER FOR DATA USE

In the project "Transformation of Artistic Style and Innovative Design of Oriental Folk Patterns Based on AIGC Technology-A Case Study of Zhuxian Town New Year Paintings from China", we (including Chang'e Kuang, Xiaotong Li, Ziwei Luo, Qinglei Du, and Zizheng Liu) were invited to make a subjective evaluation on the New Year paintings of Zhuxian Town from China generated by AIGC technology to determine the optimal remaking amplitude and keyword guidance coefficient.

We hereby grant Mr. Jinsong Kuang exclusive rights to use my evaluation data on the related academic research.

Granted by:

Chang'e Kuang: 邝嫦娥 2024.12.10.

Xiaotong Li: 李晓童 2024.12.10

Ziwei Luo: 罗紫薇 2024.12.10

Qinglei Du: 杜庆磊 2024.12.10

Zizheng Liu: 刘子政 2024.12.10

Accepted by:

Jinsong Kuang: 邝劲松 2024.12.10
