# Supplementary material for: Transformation of artistic style and innovative design of oriental folk patterns based on AIGC Technology—A case study of Zhuxian town new year paintings from China
Source: PLoS One. 2026 May 27;21(5):e0346020. doi: 10.1371/journal.pone.0346020 (PMC13215520; doi:10.1371/journal.pone.0346020)
Supplement: S6 Appendix — (PDF) [file pone.0346020.s006.pdf]

EXTERNAL EXPERT STATEMENT

Project name:
Style Transformation and Innovative Design of Oriental Folk Pattern Art Based on AIGC Technology —— Taking Chinese Zhuxian Town New Year Picture as an example

Chang'e Kuang; Xiaotong Li; Ziwei Luo; Qinglei Du; Zizheng Liu solemn declaration:

Independence: did not participate in any technical development such as algorithm design, data training or parameter adjustment.

Conflict of interest: I and my institution have no teacher-student relationship, project cooperation or economic interest with the research team.

Confidentiality: Strict confidentiality of unpublished technical details and research data encountered in the review process.

Evaluation criteria: the commitment to objective scoring is not affected by external factors.

Signature: 邝嫦娥 李晓童 罗紫薇 杜庆磊 [illegible]
Date: 2024.12.10
