# Supplementary material for: Transformation of artistic style and innovative design of oriental folk patterns based on AIGC Technology—A case study of Zhuxian town new year paintings from China
Source: PLoS One. 2026 May 27;21(5):e0346020. doi: 10.1371/journal.pone.0346020 (PMC13215520; doi:10.1371/journal.pone.0346020)
Supplement: S8 Appendix — (DOCX) [file pone.0346020.s008.docx]

# **Data Anonymization Instructions**

To protect the privacy and security of survey participants, ensure data use complies with academic ethics, and maintain the analytical value and integrity of the data, the data anonymization work for this survey on the core artistic characteristics preferences of Zhuxian Town figure New Year paintings strictly adhered to the principles of "minimum necessity, traceability, and no association." Multi-dimensional technical methods were employed to process the original data. The specific steps are as follows:

# **I. Anonymization of Personal Identification Information**

1. Name Substitution: In the original data collected from the survey questionnaires, any name information voluntarily provided by the respondents was replaced using anonymous coding. The coding rule was "sample number + random check digit," meaning a unique number (1-40) was assigned sequentially according to the order of questionnaire collection, without any association with the individual's real identity, completely severing the correspondence between the number and the name.

2. Contact Information Anonymization: For the optional "Reserved Email Address" section in the questionnaire, a dual anonymization strategy is employed: First, the complete email address is directly hidden, with only the "Yes/No" indicator remaining in the data storage for statistical purposes regarding feedback intentions. Second, pre-reserved email addresses undergo partial character masking (e.g., "xxx@xxx.com" is masked to "xx@x*.com"), used only for subsequent targeted sending of survey result summaries, and not included in any public data or academic analysis.

3. Removal of Specific Occupational Details: For the specific occupational names indicated by respondents in the "Other" category (e.g., freelancer, corporate employee, retiree, student, medical professional, programmer, civil servant, entrepreneur, etc.), only the "Other" category label is retained in public data and analysis reports, removing detailed occupational descriptions to avoid identifying individuals based on occupational characteristics.

# **II. Generalized Processing of Demographic Information**

1. Age Information Interval Conversion: Precise age data was not collected in the original questionnaire. During the design phase, interval options (20-30 years, 31-40 years, 41-50 years, 51-55 years) were used for data collection, preventing the leakage of precise age information from the outset. During the data processing phase, it was further confirmed that no additional precise age records were found, and the interval data was directly used, ensuring both privacy and meeting the statistical analysis needs of the age dimension.

1. Regional Information Aggregation: The geographical location of the survey participants was simplified, aggregating specific province, city, and autonomous region information into four major regional categories (North China, East China, South China, and Other). The specific regional information under the "Other" category in the original questionnaire was removed, retaining only the "Other" regional identifier, avoiding narrowing the individual's scope through geographic location.

# **III. Data Correlation Severing Process**

1. Independent Presentation of Single-Dimensional Data: In the publicly available raw data tables, only the correspondence between sample numbers and each survey dimension (occupation category, age range, region, preference characteristics, etc.) is retained. No additional auxiliary information that could be used to link individuals (such as submission time, IP address, device information, etc.) is added, ensuring that each dimension's data serves only statistical analysis and cannot be used to locate specific survey subjects through multi-dimensional cross-referencing.

2. Meaningless Sample Numbering: Sample numbers use only a continuous numerical sequence (1-40), without embedding any feature codes related to the survey subjects (such as age range abbreviations, region codes, etc.). The numbers themselves do not have any identity-related meaning and serve only as temporary identifiers for data processing and analysis.

IV. Data Storage and Dissemination Anonymization Enhancement

1. Encrypted Storage of Raw Data: Raw data containing some not fully disclosed information (such as masked email addresses) is stored using the AES-256 encryption algorithm. Only authorized researchers can access it via a key, preventing the risk of data leakage.

2. Desensitization and Screening of Public Data: For data used in public settings such as academic papers and research reports, only anonymized sample numbers, categorized demographic information, and core preference data are retained. All redundant information that may involve personal privacy is completely removed (e.g., personalized descriptions in supplementary suggestions that contain identity clues have been anonymized or deleted).

3. Data Usage Access Control: The scope of use of the anonymized data is clearly defined as limited to academic activities related to "Research on the Artistic Characteristics of Zhuxian Town Figure New Year Paintings," and is prohibited for commercial use or other non-research scenarios. If data sharing is required, the completeness of data anonymization must be reconfirmed, and a data security and confidentiality agreement must be signed with the recipient.

V. Verification of Anonymization Effectiveness

1. Logical Verification: Through cross-comparison of the anonymized data across various dimensions, it is confirmed that no single dimension or combination of dimensions can pinpoint a specific individual, and data correlations are completely severed.

2. Completeness Verification: After anonymization, core survey data (preference themes, composition, color, etc.) are complete and without distortion, and the sample distribution characteristics (occupation, age, regional proportion) are consistent with the original data, meeting the needs of academic analysis.

3. Compliance verification: The anonymization process complies with the requirements of relevant laws and regulations such as the Personal Information Protection Law and the Measures for the Management of Data Security in Scientific Research Activities, ensuring the right to know and the right to privacy of the survey participants.
