# Supplementary material for: Transformation of artistic style and innovative design of oriental folk patterns based on AIGC Technology—A case study of Zhuxian town new year paintings from China
Source: PLoS One. 2026 May 27;21(5):e0346020. doi: 10.1371/journal.pone.0346020 (PMC13215520; doi:10.1371/journal.pone.0346020)
Supplement: S9 Appendix — (DOCX) [file pone.0346020.s009.docx]

# **Text of article 24 of copyright law**

Article 24. In the following circumstances, the use of a work may be made without the copyright owner's permission and without payment of remuneration, provided that the author's name or title and the work's title are indicated, and that the normal use of the work is not affected or the copyright owner's legitimate rights and interests are not unreasonably infringed:

(1) Using published works of others for personal study, research, or appreciation;

(2) To appropriately quote published works of others in a work for the purpose of introducing or commenting on a particular work or explaining a certain issue;

(3) To report news, it is inevitable to reproduce or quote published works in newspapers, periodicals, radio stations, television stations and other media;

(4) The publication or broadcasting of current affairs articles on political, economic or religious issues by newspapers, periodicals, radio or television stations, which have already been published by other media, is permitted unless the copyright owner expressly prohibits such publication or broadcasting.

(5) Speeches delivered at public gatherings published in newspapers, periodicals, radio or television programs, unless the author expressly declines such publication or broadcasting.

(6) For the purpose of classroom teaching or scientific research, the translation, adaptation, compilation, broadcasting, or limited reproduction of published works may be used by teaching or research personnel, but such works shall not be published or distributed;

(7) State organs may use published works within reasonable limits for official purposes;

(8) Libraries, archives, memorial halls, museums, art galleries, and cultural centers may reproduce works in their collections for exhibition or preservation purposes.

(9) Free performance of published works, where no fees are charged to the public, no remuneration is paid to the performers, and the performance is not for profit;

(10) Copying, painting, photographing, or filming of artworks installed or displayed in public places;

(11) To translate works created in the national common language and script that have already been published by China citizens, legal persons or non-legal person organizations into works in minority languages and scripts for domestic publication and distribution;

(12) Provide published works to individuals with dyslexia in accessible formats that they can perceive;

(13) Other circumstances prescribed by laws and administrative regulations.

The provisions of the preceding paragraph shall apply to the limitation of the rights related to copyright.
