# Supplementary material for: Transformation of artistic style and innovative design of oriental folk patterns based on AIGC Technology—A case study of Zhuxian town new year paintings from China
Source: PLoS One. 2026 May 27;21(5):e0346020. doi: 10.1371/journal.pone.0346020 (PMC13215520; doi:10.1371/journal.pone.0346020)
Supplement: S10 Appendix — (PDF) [file pone.0346020.s010.pdf]

INFORMED CONSENT

1. Research information notification

The research you will be involved in involves:

The style compliance of 50 sets of AIGC-generated New Year pictures was evaluated

The results of the review will be used to determine the optimal redrawing amplitude and keyword guidance coefficient, which will be cited in academic papers

2. Participation rights and risks

right:

Unconditional withdrawal at any time without assuming any responsibility

Request to delete personal review data (before submission)

Risks: No direct physical risk

3.Confidentiality and data use

Your identity will be treated anonymously

The original scoring results are only available to the research team

4. Consent statement

☑ I have read and understood the above

☑ I volunteer to participate in this study

☑ I agree to use my review data anonymously in the paper

Signature: 祁[illegible] 李晓蕾 罗紫薇 杜庆[illegible] 刘[illegible]

Date: 2024.12.10
