# Supplementary material for: Transformation of artistic style and innovative design of oriental folk patterns based on AIGC Technology—A case study of Zhuxian town new year paintings from China
Source: PLoS One. 2026 May 27;21(5):e0346020. doi: 10.1371/journal.pone.0346020 (PMC13215520; doi:10.1371/journal.pone.0346020)
Supplement: S11 Appendix — (DOCX) [file pone.0346020.s011.docx]

Appendix 4: Training Model Evolution

Table 1 Parameter Settings of the first LORA training model

| parameter | Parameter values |
| --- | --- |
| bottom die | SD1.5_TuCiv_CNMale_1.0 |
| Number of single training sessions | 8 |
| Number of training rounds | 10 |
| batch size | 3 |
| Train the hybrid precision | fp16 |
| Sample resolution | 512x512 |
| Random seed count | 1000000001 |
| sample mode | DPM++ 2M Karras |
| call-word |  |
| Negative prompts | (worst quality, low quality:1.4),(depth of field, blurry:1.2),(greyscale, monochrome:1.1),3D face,cropped,lowres,text,(nsfw:1.3),(worst quality:2),(low quality:2),(normal quality:2),normal quality,((grayscale)),skin spots,acnes,skin blemishes,age spot,(ugly:1.331),(duplicate:1.331),(morbid:1.21),(mutilated:1.21),(tranny:1.331),mutated hands,(poorly drawn hands:1.5),blurry,(bad anatomy:1.21),(bad proportions:1.331),extra limbs,(disfigured:1.331),(missing arms:1.331),(extra legs:1.331),(fused fingers:1.61051),(too many fingers:1.61051),(unclear eyes:1.331),lowers,bad hands,missing fingers,extra digit,bad hands,missing fingers,(((extra arms and legs))) |
| Save a LoRA every N rounds | 2 |
| LoRA preserves accuracy | fp16 |
| Total learning rate | 1e-4 |
| Unet learning rate | 0.0001 |
| Text encoder learning rate | 0.00001 |
| Learning rate scheduler | cosine_with_restarts |
| optimizer | AdamW8bit |
| Restart count | 1 |
| Network size | 102 |
| network Alpha | 64 |
| Keep n tokens | 18 |
| Maximum token length | 75 |
| Noise offset | 0.1 |
| Random seed count | -1 |
| clip skip | 2 |


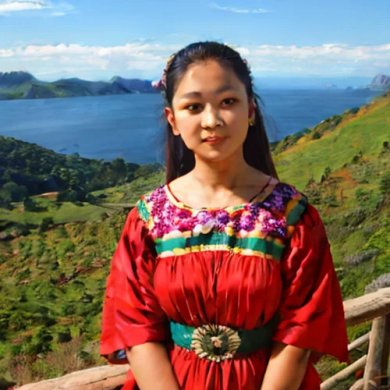

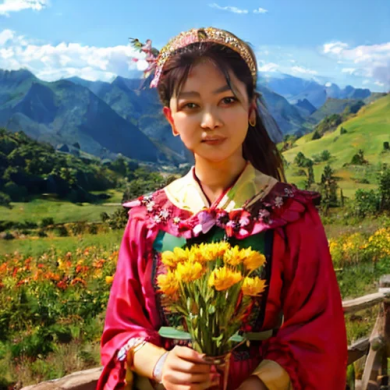

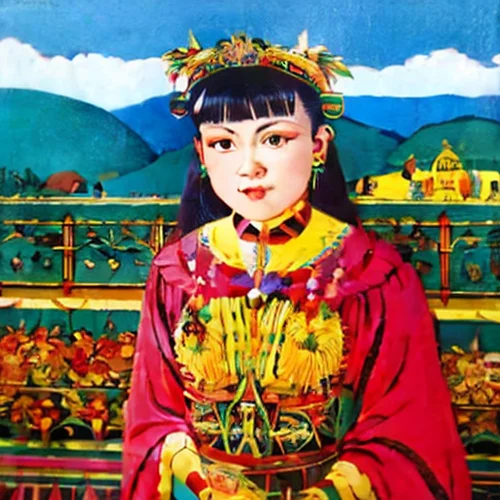

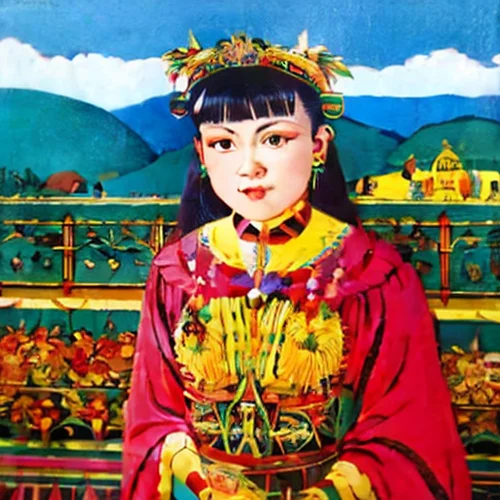

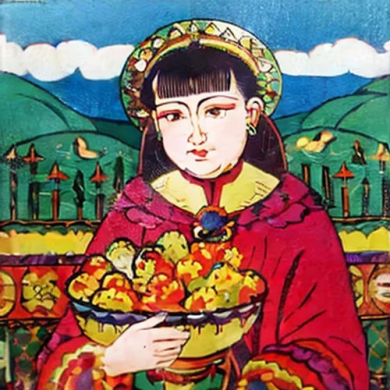


Figure 1. The first LORA training model

Table 2 Parameter Settings of the second LORA training model

| parameter | Parameter values |
| --- | --- |
| bottom die | Mianzhu New Year paintings, Sichuan province 2024NewYear |
| Number of single training sessions | 12 |
| Number of training rounds | 10 |
| batch size | 2 |
| Train the hybrid precision | fp16 |
| Sample resolution | 512x512 |
| Random seed count | 1000000001 |
| sample mode | DPM++ 2M Karras |
| call-word | the lines are rough, thick and fine，exaggerated image, big head and small body，the composition is full, symmetrical left and right， the color is gorgeous and the contrast is strong，the door god God code, serious and dignified， |
| Negative prompts | (worst quality, low quality:1.4),(depth of field, blurry:1.2),(greyscale, monochrome:1.1),3D face,cropped,lowres,text,(nsfw:1.3),(worst quality:2),(low quality:2),(normal quality:2),normal quality,((grayscale)),skin spots,acnes,skin blemishes,age spot,(ugly:1.331),(duplicate:1.331),(morbid:1.21),(mutilated:1.21),(tranny:1.331),mutated hands,(poorly drawn hands:1.5),blurry,(bad anatomy:1.21),(bad proportions:1.331),extra limbs,(disfigured:1.331),(missing arms:1.331),(extra legs:1.331),(fused fingers:1.61051),(too many fingers:1.61051),(unclear eyes:1.331),lowers,bad hands,missing fingers,extra digit,bad hands,missing fingers,(((extra arms and legs))) |
| Save a LoRA every N rounds | 2 |
| LoRA preserves accuracy | fp16 |
| Total learning rate | 1e-4 |
| Unet learning rate | 0.0001 |
| Text encoder learning rate | 0.00001 |
| Learning rate scheduler | cosine_with_restarts |
| optimizer | AdamW8bit |
| Restart count | 1 |
| Network size | 128 |
| network Alpha | 64 |
| Keep n tokens | 1 |
| Maximum token length | 75 |
| Noise offset | 0.1 |
| Random seed count | -1 |
| clip skip | 1 |


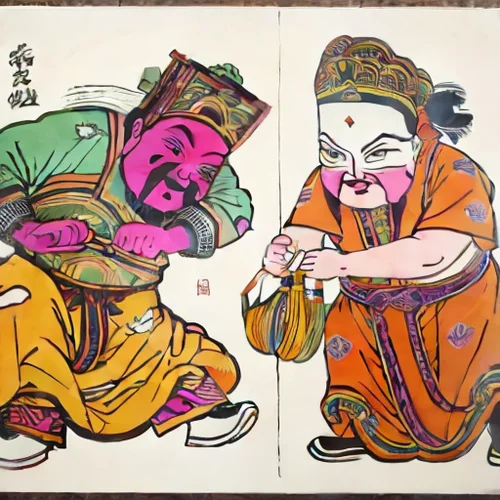

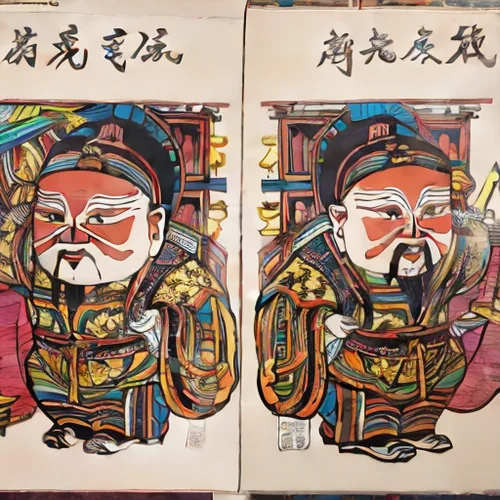

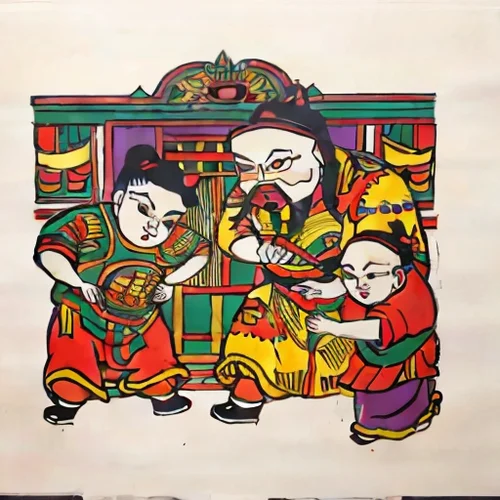

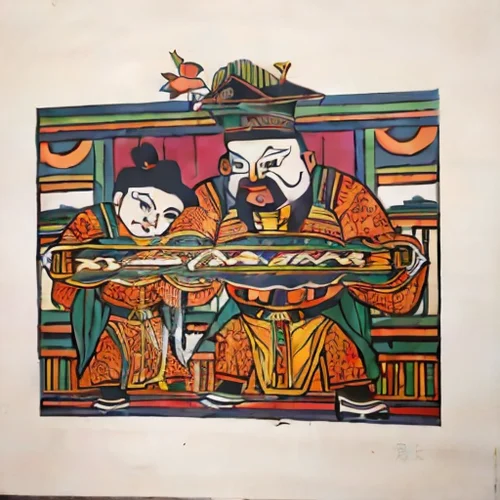

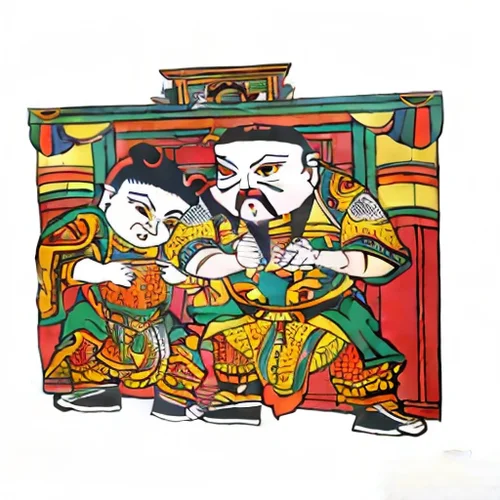


Figure 2. The second LORA training model

Table 3 Parameter Settings of the third LORA training model

| parameter | Parameter values |
| --- | --- |
| bottom die | Sichuan Mianzhu New Year painting _2024NewYear |
| Number of single training sessions | 12 |
| Number of training rounds | 10 |
| batch size | 2 |
| Train the hybrid precision | fp16 |
| Sample resolution | 512x512 |
| Random seed count | 1000000001 |
| sample mode | DPM++ 2M Karras |
| call-word | the lines are rough, thick and fine，exaggerated image, big head and small body，the composition is full, symmetrical left and right， the color is gorgeous and the contrast is strong，the door god God code, serious and dignified， |
| Negative prompts | (worst quality, low quality:1.4),(depth of field, blurry:1.2),(greyscale, monochrome:1.1),3D face,cropped,lowres,text,(nsfw:1.3),(worst quality:2),(low quality:2),(normal quality:2),normal quality,((grayscale)),skin spots,acnes,skin blemishes,age spot,(ugly:1.331),(duplicate:1.331),(morbid:1.21),(mutilated:1.21),(tranny:1.331),mutated hands,(poorly drawn hands:1.5),blurry,(bad anatomy:1.21),(bad proportions:1.331),extra limbs,(disfigured:1.331),(missing arms:1.331),(extra legs:1.331),(fused fingers:1.61051),(too many fingers:1.61051),(unclear eyes:1.331),lowers,(((bad hands))),missing fingers,extra digit,bad hands,missing fingers,(((extra arms and legs))),(((malformed hands))),(((cross-eyed))) |
| Save a LoRA every N rounds | 2 |
| LoRA preserves accuracy | fp16 |
| Total learning rate | 1e-4 |
| Unet learning rate | 0.0001 |
| Text encoder learning rate | 0.00001 |
| Learning rate scheduler | cosine_with_restarts |
| optimizer | AdamW8bit |
| Restart count | 1 |
| Network size | 128 |
| network Alpha | 64 |
| Keep n tokens | 1 |
| Maximum token length | 75 |
| Noise offset | 0.1 |
| Random seed count | -1 |
| clip skip | 1 |


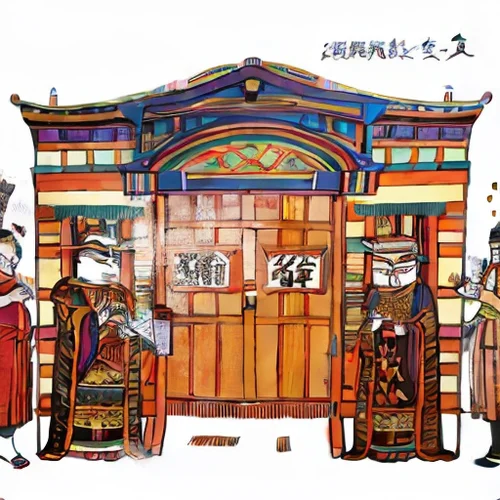

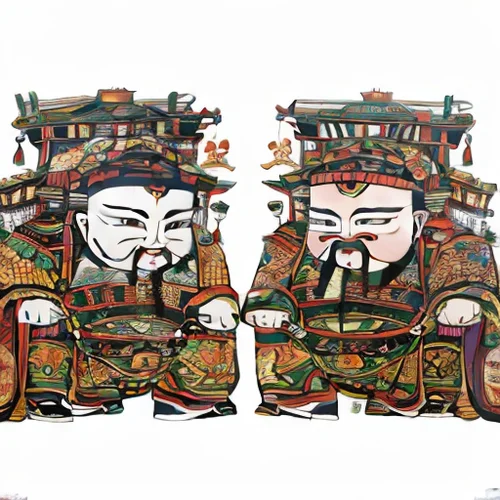

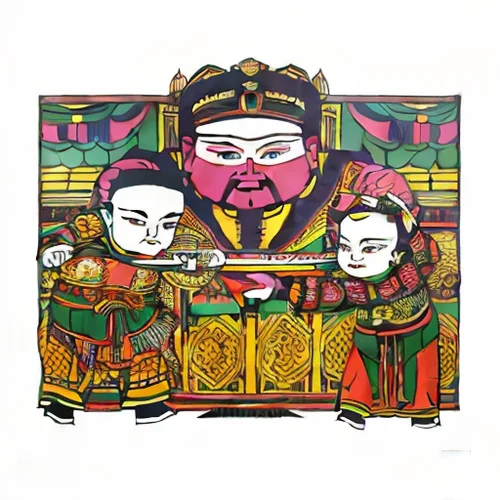

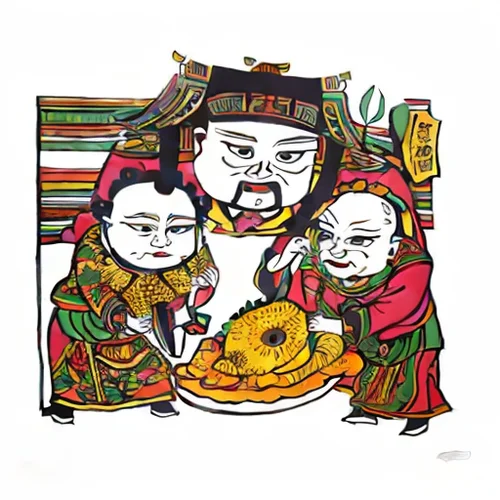

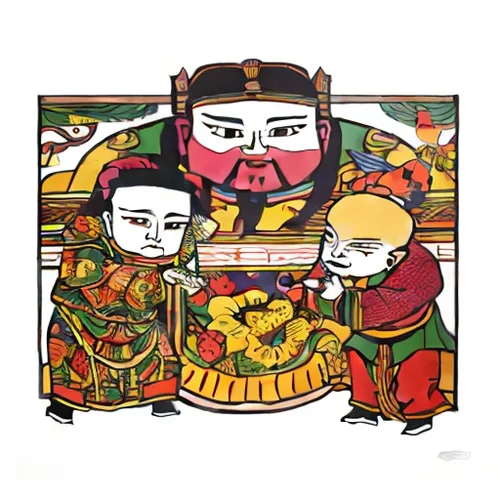


Figure 3 Third LORA training model

Table 4 Parameter Settings of LORA Training Model for the fourth time

| parameter | Parameter values |
| --- | --- |
| bottom die | Sichuan Mianzhu New Year painting _2024NewYear |
| Number of single training sessions | 16 |
| Number of training rounds | 9 |
| batch size | 2 |
| Train the hybrid precision | fp16 |
| Sample resolution | 512x512 |
| Random seed count | 1000000001 |
| sample mode | DPM++ 2M Karras |
| call-word | DPM++ 2M Karras |
| Negative prompts | (worst quality, low quality:1.4),(depth of field, blurry:1.2),(greyscale, monochrome:1.1),3D face,cropped,lowres,text,(nsfw:1.3),(worst quality:2),(low quality:2),(normal quality:2),normal quality,((grayscale)),skin spots,acnes,skin blemishes,age spot,(ugly:1.331),(duplicate:1.331),(morbid:1.21),(mutilated:1.21),(tranny:1.331),mutated hands,(poorly drawn hands:1.5),blurry,(bad anatomy:1.21),(bad proportions:1.331),extra limbs,(disfigured:1.331),(missing arms:1.331),(extra legs:1.331),(fused fingers:1.61051),(too many fingers:1.61051),(unclear eyes:1.331),lowers,bad hands,missing fingers,extra digit,bad hands,missing fingers,(((extra arms and legs))) |
| Save a LoRA every N rounds | 2 |
| LoRA preserves accuracy | fp16 |
| Total learning rate | 1e-4 |
| Unet learning rate | 0.0001 |
| Text encoder learning rate | 0.00001 |
| Learning rate scheduler | cosine_with_restarts |
| optimizer | AdamW8bit |
| Restart count | 1 |
| Network size | 128 |
| network Alpha | 64 |
| Keep n tokens | 64 |
| Maximum token length | 75 |
| Noise offset | 75 |
| Random seed count | -1 |
| clip skip | 1 |


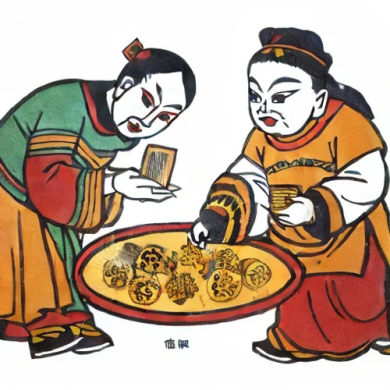

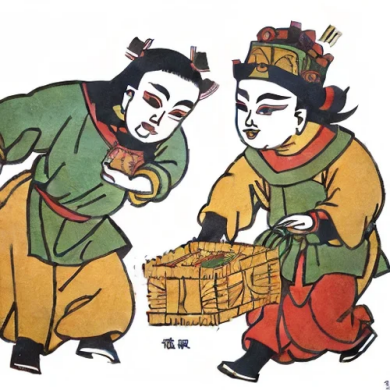

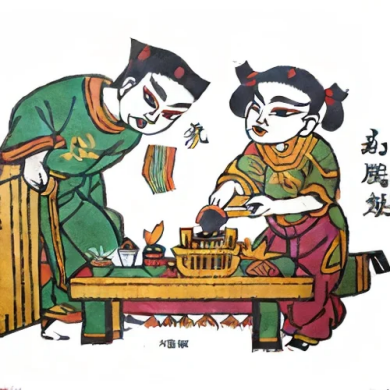


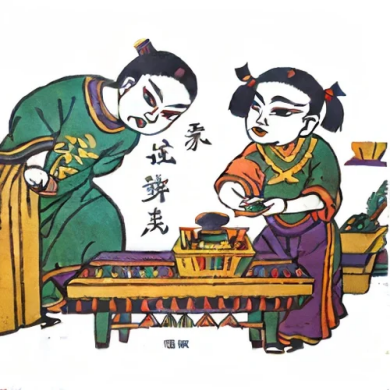

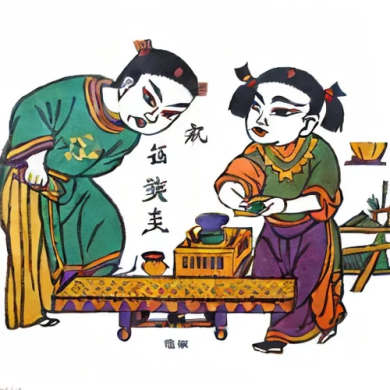


Figure 4 LORA optimal model
