# Supplementary material for: Transformation of artistic style and innovative design of oriental folk patterns based on AIGC Technology—A case study of Zhuxian town new year paintings from China
Source: PLoS One. 2026 May 27;21(5):e0346020. doi: 10.1371/journal.pone.0346020 (PMC13215520; doi:10.1371/journal.pone.0346020)
Supplement: S12 Appendix — (DOC) [file pone.0346020.s012.doc]

1、Fig 11-12 Generation parameters for image types under the LORA optimal model


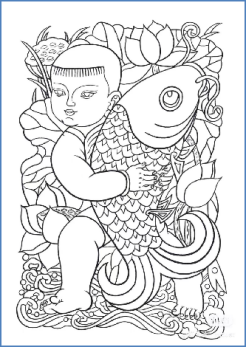


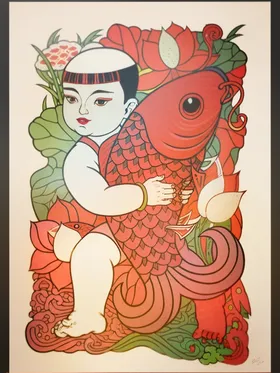

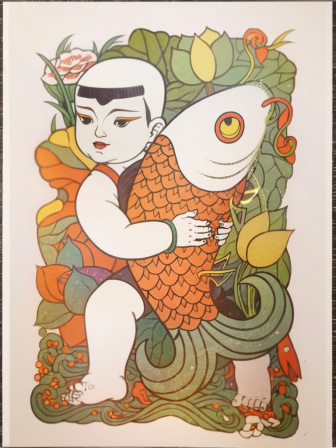

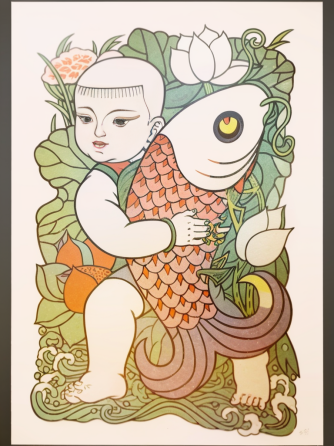


| 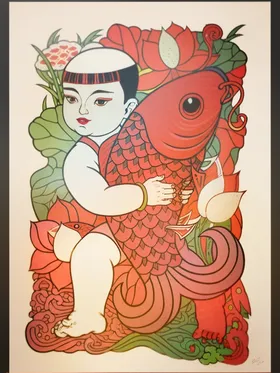 | Prompt: 1boy,red flower,black hair,(((red fish))),male focus,white background,simple background,barefoot,traditional media,jewelry,plant,black eyes,(white face),((((black hair)))),(red lipstick),(((red fish head))),  Negative prompt: ng_deepnegative_v1_75t,(badhandv4:1.2),EasyNegative,(worst quality:2),lowres,half-closed eyes,bad hands,bad feet,goldfish too big,  Steps: 25,  Size: 768x1024,  Seed: 3297603495,  Model: 四川绵竹年画,20250103-1735889476394-0005,  Sampler: 15,  CFG scale: 7 |
| --- | --- |
| 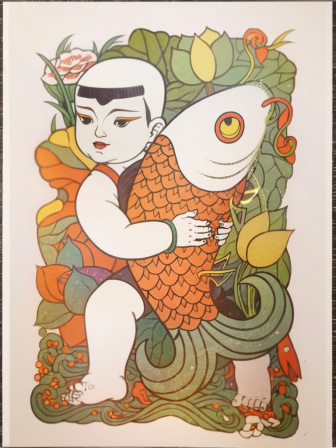 | Prompt: Chinese traditional New Year picture style,traditional medie,solo,white background,full body,jewelry,black eyes,male focus,black hair,barefoot,leaf,flower,plant,bracelet,makeup,holding,1boy,goldfish,  Negative prompt: ng_deepnegative_v1_75t,(badhandv4:1.2),EasyNegative,(worst quality:2),lowres,half-closed eyes,bad hands,bad feet,goldfish too big,  Steps: 25,  Size: 768x1024,  Seed: 1445577645,  Model: 四川绵竹年画,20250103-1735889487738-0003,  Sampler: 15,  CFG scale: 7 |
| 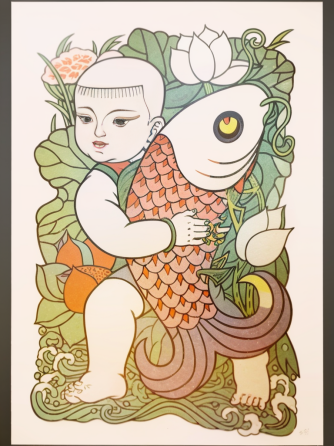 | Prompt: Chinese traditional New Year picture style,traditional medie,solo,white background,full body,jewelry,black eyes,male focus,black hair,barefoot,leaf,flower,plant,bracelet,makeup,holding,1boy,goldfish,  Negative prompt: ng_deepnegative_v1_75t,(badhandv4:1.2),EasyNegative,(worst quality:2),lowres,half-closed eyes,bad hands,bad feet,goldfish too big,  Steps: 25,  Size: 768x1024,  Seed: 2525980124,  Model: 四川绵竹年画,20250103-1735889487738-0003,  Sampler: 15,  CFG scale: 7 |

2、Fig 15 Parameter adjustment effects (1): generation parameters

| 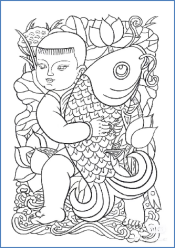LORA weight 1.3 | Redraw amplitude 0 | Redraw amplitude 0.3 | Redraw amplitude 0.5 | Redraw amplitude 0.7 | Redraw amplitude 1.0 |
| --- | --- | --- | --- | --- | --- |
| CFG2 | 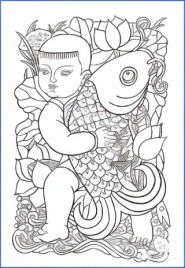  ,1boy,red flower,black hair,(((red fish))),male focus,white background,simple background,barefoot,traditional media,jewelry,plant,black eyes,white face,  Negative prompt: ng_deepnegative_v1_75t,(badhandv4:1.2),EasyNegative,(worst quality:2),lowres,half-closed eyes,bad hands,bad feet,goldfish too big,  Steps: 20, Size: 320x464, Seed: 3771120027, Model: 四川绵竹年画,20250103-1735889476394-0005, Sampler: 1, CFG scale: 2 | 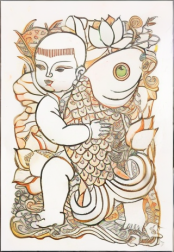  ,1boy,red flower,black hair,(((red fish))),male focus,white background,simple background,barefoot,traditional media,jewelry,plant,black eyes,white face,  Negative prompt: ng_deepnegative_v1_75t,(badhandv4:1.2),EasyNegative,(worst quality:2),lowres,half-closed eyes,bad hands,bad feet,goldfish too big,  Steps: 20, Size: 320x464, Seed: 3394676361, Model: 四川绵竹年画,20250103-1735889476394-0005, Sampler: 1, CFG scale: 2 | 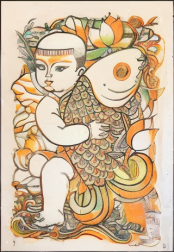  ,1boy,red flower,black hair,(((red fish))),male focus,white background,simple background,barefoot,traditional media,jewelry,plant,black eyes,white face,  Negative prompt: ng_deepnegative_v1_75t,(badhandv4:1.2),EasyNegative,(worst quality:2),lowres,half-closed eyes,bad hands,bad feet,goldfish too big,  Steps: 20, Size: 320x464, Seed: 613191992, Model: 四川绵竹年画,20250103-1735889476394-0005, Sampler: 1, CFG scale: 2 | 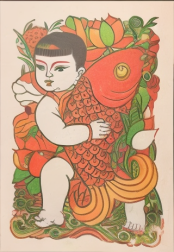  ,1boy,red flower,black hair,(((red fish))),male focus,white background,simple background,barefoot,traditional media,jewelry,plant,black eyes,white face,  Negative prompt: ng_deepnegative_v1_75t,(badhandv4:1.2),EasyNegative,(worst quality:2),lowres,half-closed eyes,bad hands,bad feet,goldfish too big,  Steps: 20, Size: 320x464, Seed: 3473204131, Model: 四川绵竹年画,20250103-1735889476394-0005, Sampler: 1, CFG scale: 2 | 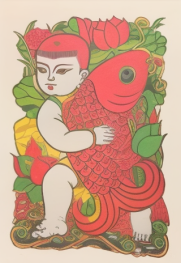  ,1boy,red flower,black hair,(((red fish))),male focus,white background,simple background,barefoot,traditional media,jewelry,plant,black eyes,white face,  Negative prompt: ng_deepnegative_v1_75t,(badhandv4:1.2),EasyNegative,(worst quality:2),lowres,half-closed eyes,bad hands,bad feet,goldfish too big,  Steps: 20, Size: 320x464, Seed: 2015116440, Model: 四川绵竹年画,20250103-1735889476394-0005, Sampler: 1, CFG scale: 2 |
| CFG5 | 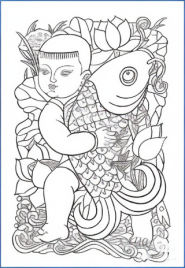  ,1boy,red flower,black hair,(((red fish))),male focus,white background,simple background,barefoot,traditional media,jewelry,plant,black eyes,white face,  Negative prompt: ng_deepnegative_v1_75t,(badhandv4:1.2),EasyNegative,(worst quality:2),lowres,half-closed eyes,bad hands,bad feet,goldfish too big,  Steps: 20, Size: 320x464, Seed: 3345200337, Model: 四川绵竹年画,20250103-1735889476394-0005, Sampler: 1, CFG scale: 5 | 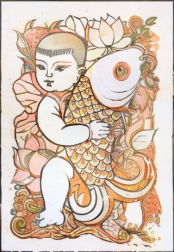  ,1boy,red flower,black hair,(((red fish))),male focus,white background,simple background,barefoot,traditional media,jewelry,plant,black eyes,white face,  Negative prompt: ng_deepnegative_v1_75t,(badhandv4:1.2),EasyNegative,(worst quality:2),lowres,half-closed eyes,bad hands,bad feet,goldfish too big,  Steps: 20, Size: 320x464, Seed: 1380337541, Model: 四川绵竹年画,20250103-1735889476394-0005, Sampler: 1, CFG scale: 5 | 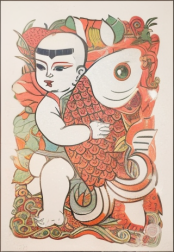  ,1boy,red flower,black hair,(((red fish))),male focus,white background,simple background,barefoot,traditional media,jewelry,plant,black eyes,white face,  Negative prompt: ng_deepnegative_v1_75t,(badhandv4:1.2),EasyNegative,(worst quality:2),lowres,half-closed eyes,bad hands,bad feet,goldfish too big,  Steps: 20, Size: 320x464, Seed: 506647831, Model: 四川绵竹年画,20250103-1735889476394-0005, Sampler: 1, CFG scale: 5 | 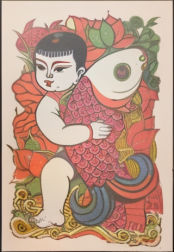  ,1boy,red flower,black hair,(((red fish))),male focus,white background,simple background,barefoot,traditional media,jewelry,plant,black eyes,white face,  Negative prompt: ng_deepnegative_v1_75t,(badhandv4:1.2),EasyNegative,(worst quality:2),lowres,half-closed eyes,bad hands,bad feet,goldfish too big,  Steps: 20, Size: 320x464, Seed: 506647831, Model: 四川绵竹年画,20250103-1735889476394-0005, Sampler: 1, CFG scale: 5 | 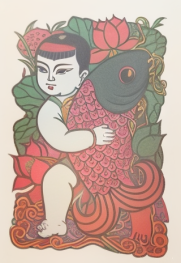  ,1boy,red flower,black hair,(((red fish))),male focus,white background,simple background,barefoot,traditional media,jewelry,plant,black eyes,white face,  Negative prompt: ng_deepnegative_v1_75t,(badhandv4:1.2),EasyNegative,(worst quality:2),lowres,half-closed eyes,bad hands,bad feet,goldfish too big,  Steps: 20, Size: 320x464, Seed: 1637609886, Model: 四川绵竹年画,20250103-1735889476394-0005, Sampler: 1, CFG scale: 5 |
| CFG6.5 | 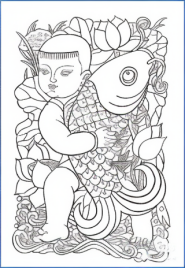  ,1boy,red flower,black hair,(((red fish))),male focus,white background,simple background,barefoot,traditional media,jewelry,plant,black eyes,white face,  Negative prompt: ng_deepnegative_v1_75t,(badhandv4:1.2),EasyNegative,(worst quality:2),lowres,half-closed eyes,bad hands,bad feet,goldfish too big,  Steps: 20, Size: 320x464, Seed: 2696791887, Model: 四川绵竹年画,20250103-1735889476394-0005, Sampler: 1, CFG scale: 6.5 | 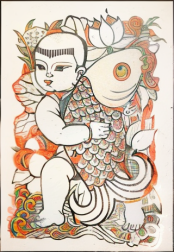  ,1boy,red flower,black hair,(((red fish))),male focus,white background,simple background,barefoot,traditional media,jewelry,plant,black eyes,white face,  Negative prompt: ng_deepnegative_v1_75t,(badhandv4:1.2),EasyNegative,(worst quality:2),lowres,half-closed eyes,bad hands,bad feet,goldfish too big,  Steps: 20, Size: 320x464, Seed: 20059307, Model: 四川绵竹年画,20250103-1735889476394-0005, Sampler: 1, CFG scale: 6.5 | 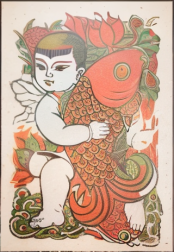  ,1boy,red flower,black hair,(((red fish))),male focus,white background,simple background,barefoot,traditional media,jewelry,plant,black eyes,white face,  Negative prompt: ng_deepnegative_v1_75t,(badhandv4:1.2),EasyNegative,(worst quality:2),lowres,half-closed eyes,bad hands,bad feet,goldfish too big,  Steps: 20, Size: 320x464, Seed: 4113800043, Model: 四川绵竹年画,20250103-1735889476394-0005, Sampler: 1, CFG scale: 6.5 | 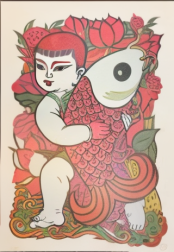  ,1boy,red flower,black hair,(((red fish))),male focus,white background,simple background,barefoot,traditional media,jewelry,plant,black eyes,white face,  Negative prompt: ng_deepnegative_v1_75t,(badhandv4:1.2),EasyNegative,(worst quality:2),lowres,half-closed eyes,bad hands,bad feet,goldfish too big,  Steps: 20, Size: 320x464, Seed: 1244409490, Model: 四川绵竹年画,20250103-1735889476394-0005, Sampler: 1, CFG scale: 6.5 | 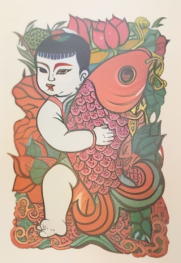  ,1boy,red flower,black hair,(((red fish))),male focus,white background,simple background,barefoot,traditional media,jewelry,plant,black eyes,white face,  Negative prompt: ng_deepnegative_v1_75t,(badhandv4:1.2),EasyNegative,(worst quality:2),lowres,half-closed eyes,bad hands,bad feet,goldfish too big,  Steps: 20, Size: 320x464, Seed: 3023548796, Model: 四川绵竹年画,20250103-1735889476394-0005, Sampler: 1, CFG scale: 6.5 |
| CFG8 | 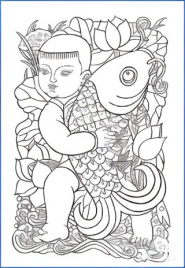  ,1boy,red flower,black hair,(((red fish))),male focus,white background,simple background,barefoot,traditional media,jewelry,plant,black eyes,white face,  Negative prompt: ng_deepnegative_v1_75t,(badhandv4:1.2),EasyNegative,(worst quality:2),lowres,half-closed eyes,bad hands,bad feet,goldfish too big,  Steps: 20, Size: 320x464, Seed: 3106353578, Model: 四川绵竹年画,20250103-1735889476394-0005, Sampler: 1, CFG scale: 8 | 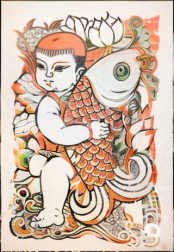  ,1boy,red flower,black hair,(((red fish))),male focus,white background,simple background,barefoot,traditional media,jewelry,plant,black eyes,white face,  Negative prompt: ng_deepnegative_v1_75t,(badhandv4:1.2),EasyNegative,(worst quality:2),lowres,half-closed eyes,bad hands,bad feet,goldfish too big,  Steps: 20, Size: 320x464, Seed: 1657528694, Model: 四川绵竹年画,20250103-1735889476394-0005, Sampler: 1, CFG scale: 8 | 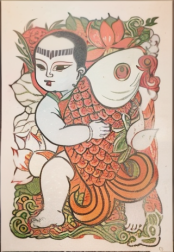  ,1boy,red flower,black hair,(((red fish))),male focus,white background,simple background,barefoot,traditional media,jewelry,plant,black eyes,white face,  Negative prompt: ng_deepnegative_v1_75t,(badhandv4:1.2),EasyNegative,(worst quality:2),lowres,half-closed eyes,bad hands,bad feet,goldfish too big,  Steps: 20, Size: 320x464, Seed: 3061609018, Model: 四川绵竹年画,20250103-1735889476394-0005, Sampler: 1, CFG scale: 8 | 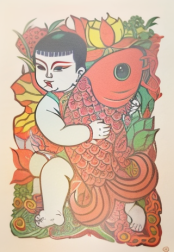  ,1boy,red flower,black hair,(((red fish))),male focus,white background,simple background,barefoot,traditional media,jewelry,plant,black eyes,white face,  Negative prompt: ng_deepnegative_v1_75t,(badhandv4:1.2),EasyNegative,(worst quality:2),lowres,half-closed eyes,bad hands,bad feet,goldfish too big,  Steps: 20, Size: 320x464, Seed: 2601090009, Model: 四川绵竹年画,20250103-1735889476394-0005, Sampler: 1, CFG scale: 8 | 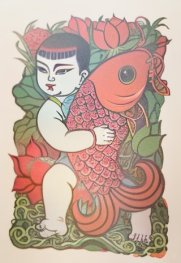  ,1boy,red flower,black hair,(((red fish))),male focus,white background,simple background,barefoot,traditional media,jewelry,plant,black eyes,white face,  Negative prompt: ng_deepnegative_v1_75t,(badhandv4:1.2),EasyNegative,(worst quality:2),lowres,half-closed eyes,bad hands,bad feet,goldfish too big,  Steps: 20, Size: 320x464, Seed: 916191467, Model: 四川绵竹年画,20250103-1735889476394-0005, Sampler: 1, CFG scale: 8 |
| CFG10 | 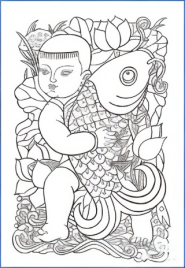  ,1boy,red flower,black hair,(((red fish))),male focus,white background,simple background,barefoot,traditional media,jewelry,plant,black eyes,white face,  Negative prompt: ng_deepnegative_v1_75t,(badhandv4:1.2),EasyNegative,(worst quality:2),lowres,half-closed eyes,bad hands,bad feet,goldfish too big,  Steps: 20, Size: 320x464, Seed: 2036687629, Model: 四川绵竹年画,20250103-1735889476394-0005, Sampler: 1, CFG scale: 10 | 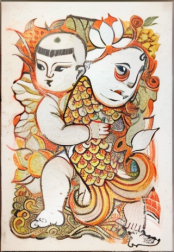  ,1boy,red flower,black hair,(((red fish))),male focus,white background,simple background,barefoot,traditional media,jewelry,plant,black eyes,white face,  Negative prompt: ng_deepnegative_v1_75t,(badhandv4:1.2),EasyNegative,(worst quality:2),lowres,half-closed eyes,bad hands,bad feet,goldfish too big,  Steps: 20, Size: 320x464, Seed: 2368114719, Model: 四川绵竹年画,20250103-1735889476394-0005, Sampler: 1, CFG scale: 10 | 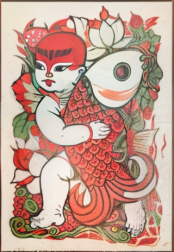  ,1boy,red flower,black hair,(((red fish))),male focus,white background,simple background,barefoot,traditional media,jewelry,plant,black eyes,white face,  Negative prompt: ng_deepnegative_v1_75t,(badhandv4:1.2),EasyNegative,(worst quality:2),lowres,half-closed eyes,bad hands,bad feet,goldfish too big,  Steps: 20, Size: 320x464, Seed: 2014466691, Model: 四川绵竹年画,20250103-1735889476394-0005, Sampler: 1, CFG scale: 10 | 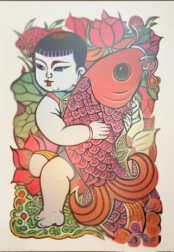  ,1boy,red flower,black hair,(((red fish))),male focus,white background,simple background,barefoot,traditional media,jewelry,plant,black eyes,white face,  Negative prompt: ng_deepnegative_v1_75t,(badhandv4:1.2),EasyNegative,(worst quality:2),lowres,half-closed eyes,bad hands,bad feet,goldfish too big,  Steps: 20, Size: 320x464, Seed: 4100907047, Model: 四川绵竹年画,20250103-1735889476394-0005, Sampler: 1, CFG scale: 10 | 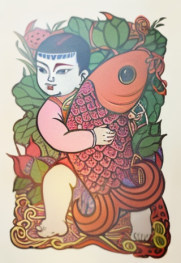  ,1boy,red flower,black hair,(((red fish))),male focus,white background,simple background,barefoot,traditional media,jewelry,plant,black eyes,white face,  Negative prompt: ng_deepnegative_v1_75t,(badhandv4:1.2),EasyNegative,(worst quality:2),lowres,half-closed eyes,bad hands,bad feet,goldfish too big,  Steps: 20, Size: 320x464, Seed: 4100907047, Model: 四川绵竹年画,20250103-1735889476394-0005, Sampler: 1, CFG scale: 10 |

3、Fig 16 Parameter adjustment effects (2)

| 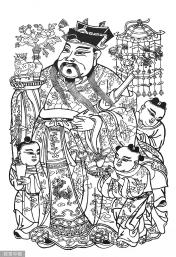  LORA weight 1.3 | Redraw amplitude 0 | Redraw amplitude 0.3 | Redraw amplitude 0.5 | Redraw amplitude 0.7 | Redraw amplitude 1.0 |
| --- | --- | --- | --- | --- | --- |
| CFG2 | 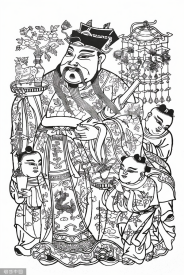  ,((((red clothes))))),black eyeliner,black beard,holding,black hair,white background,long sleeves,hat,2boys,simple background,chinese clothes,red mask:1.3,wide sleeves,black eyes,standing,1girl,((((white vase)))),((((black cup)))),((black eyeliner)),(((red flowers))),  Negative prompt: ng_deepnegative_v1_75t,(badhandv4:1.2),EasyNegative,(worst quality:2),(((negative_hand))),(((red eyeliner))),((red hand)),  Steps: 25, Size: 688x1024, Seed: 537539749, Model: 四川绵竹年画,20250103-1735889487738-0003, Sampler: 15, CFG scale: 2 | 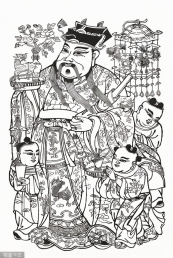  ,(red clothes)),black eyeliner,black beard,holding,black hair,white background,long sleeves,hat,2boys,simple background,chinese clothes,red mask:1.3,wide sleeves,black eyes,standing,1girl,(((((((white vase))))))),((black cup)),((black eyeliner)),(((red flowers))),  Negative prompt: ng_deepnegative_v1_75t,(badhandv4:1.2),EasyNegative,(worst quality:2),(((negative_hand))),(((red eyeliner))),((red hand)),  Steps: 25, Size: 688x1024, Seed: 3557264530, Model: 四川绵竹年画,20250103-1735889487738-0003, Sampler: 15, CFG scale: 2 | 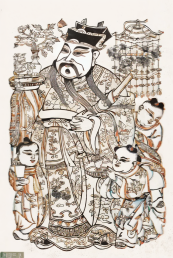  ,((((red clothes))))),black eyeliner,black beard,holding,black hair,white background,long sleeves,hat,2boys,simple background,chinese clothes,red mask:1.3,wide sleeves,black eyes,standing,1girl,((((white vase)))),((((black cup)))),((black eyeliner)),(((red flowers))),  Negative prompt: ng_deepnegative_v1_75t,(badhandv4:1.2),EasyNegative,(worst quality:2),(((negative_hand))),(((red eyeliner))),((red hand)),  Steps: 25, Size: 688x1024, Seed: 3412534876, Model: 四川绵竹年画,20250103-1735889487738-0003, Sampler: 15, CFG scale: 2 | 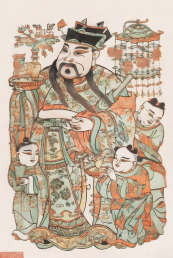  ,((((red clothes))))),black eyeliner,black beard,holding,black hair,white background,long sleeves,hat,2boys,simple background,chinese clothes,red mask:1.3,wide sleeves,black eyes,standing,1girl,((((white vase)))),((((black cup)))),((black eyeliner)),(((red flowers))),  Negative prompt: ng_deepnegative_v1_75t,(badhandv4:1.2),EasyNegative,(worst quality:2),(((negative_hand))),(((red eyeliner))),((red hand)),  Steps: 25, Size: 688x1024, Seed: 3637352211, Model: 四川绵竹年画,20250103-1735889487738-0003, Sampler: 15, CFG scale: 2 | 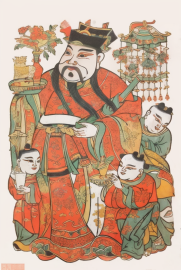  ,((((red clothes))))),black eyeliner,black beard,holding,black hair,white background,long sleeves,hat,2boys,simple background,chinese clothes,red mask:1.3,wide sleeves,black eyes,standing,1girl,((((white vase)))),((((black cup)))),((black eyeliner)),(((red flowers))),  Negative prompt: ng_deepnegative_v1_75t,(badhandv4:1.2),EasyNegative,(worst quality:2),(((negative_hand))),(((red eyeliner))),((red hand)),  Steps: 25, Size: 688x1024, Seed: 1678460594, Model: 四川绵竹年画,20250103-1735889487738-0003, Sampler: 15, CFG scale: 2 |
| CFG5 | 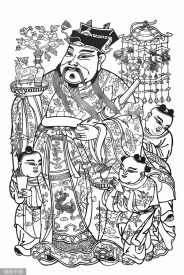  ,((((red clothes))))),black eyeliner,black beard,holding,black hair,white background,long sleeves,hat,2boys,simple background,chinese clothes,red mask:1.3,wide sleeves,black eyes,standing,1girl,((((white vase)))),((((black cup)))),((black eyeliner)),(((red flowers))),  Negative prompt: ng_deepnegative_v1_75t,(badhandv4:1.2),EasyNegative,(worst quality:2),(((negative_hand))),(((red eyeliner))),((red hand)),  Steps: 25, Size: 688x1024, Seed: 970494626, Model: 四川绵竹年画,20250103-1735889487738-0003, Sampler: 15, CFG scale: 5 | 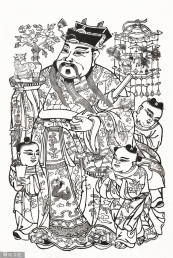  ,((((red clothes))))),black eyeliner,black beard,holding,black hair,white background,long sleeves,hat,2boys,simple background,chinese clothes,red mask:1.3,wide sleeves,black eyes,standing,1girl,((((white vase)))),((((black cup)))),((black eyeliner)),(((red flowers))),  Negative prompt: ng_deepnegative_v1_75t,(badhandv4:1.2),EasyNegative,(worst quality:2),(((negative_hand))),(((red eyeliner))),((red hand)),  Steps: 25, Size: 688x1024, Seed: 1051525785, Model: 四川绵竹年画,20250103-1735889487738-0003, Sampler: 15, CFG scale: 5 | 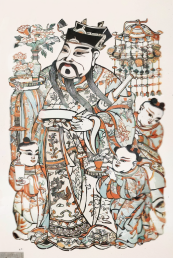  ,((((red clothes))))),black eyeliner,black beard,holding,black hair,white background,long sleeves,hat,2boys,simple background,chinese clothes,red mask:1.3,wide sleeves,black eyes,standing,1girl,((((white vase)))),((((black cup)))),((black eyeliner)),(((red flowers))),  Negative prompt: ng_deepnegative_v1_75t,(badhandv4:1.2),EasyNegative,(worst quality:2),(((negative_hand))),(((red eyeliner))),((red hand)),  Steps: 25, Size: 688x1024, Seed: 2386947089, Model: 四川绵竹年画,20250103-1735889487738-0003, Sampler: 15, CFG scale: 5 | 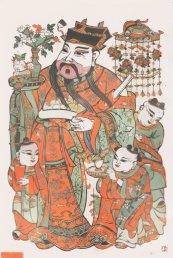  ,((((red clothes))))),black eyeliner,black beard,holding,black hair,white background,long sleeves,hat,2boys,simple background,chinese clothes,red mask:1.3,wide sleeves,black eyes,standing,1girl,((((white vase)))),((((black cup)))),((black eyeliner)),(((red flowers))),  Negative prompt: ng_deepnegative_v1_75t,(badhandv4:1.2),EasyNegative,(worst quality:2),(((negative_hand))),(((red eyeliner))),((red hand)),  Steps: 25, Size: 688x1024, Seed: 828178261, Model: 四川绵竹年画,20250103-1735889487738-0003, Sampler: 15, CFG scale: 5 | 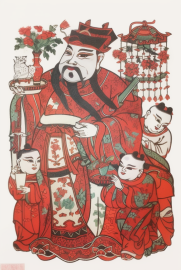,((((red clothes))))),black eyeliner,black beard,holding,black hair,white background,long sleeves,hat,2boys,simple background,chinese clothes,red mask:1.3,wide sleeves,black eyes,standing,1girl,((((white vase)))),((((black cup)))),((black eyeliner)),(((red flowers))),  Negative prompt: ng_deepnegative_v1_75t,(badhandv4:1.2),EasyNegative,(worst quality:2),(((negative_hand))),(((red eyeliner))),((red hand)),  Steps: 25, Size: 688x1024, Seed: 217242751, Model: 四川绵竹年画,20250103-1735889487738-0003, Sampler: 15, CFG scale: 5 |
| CFG6.5 | 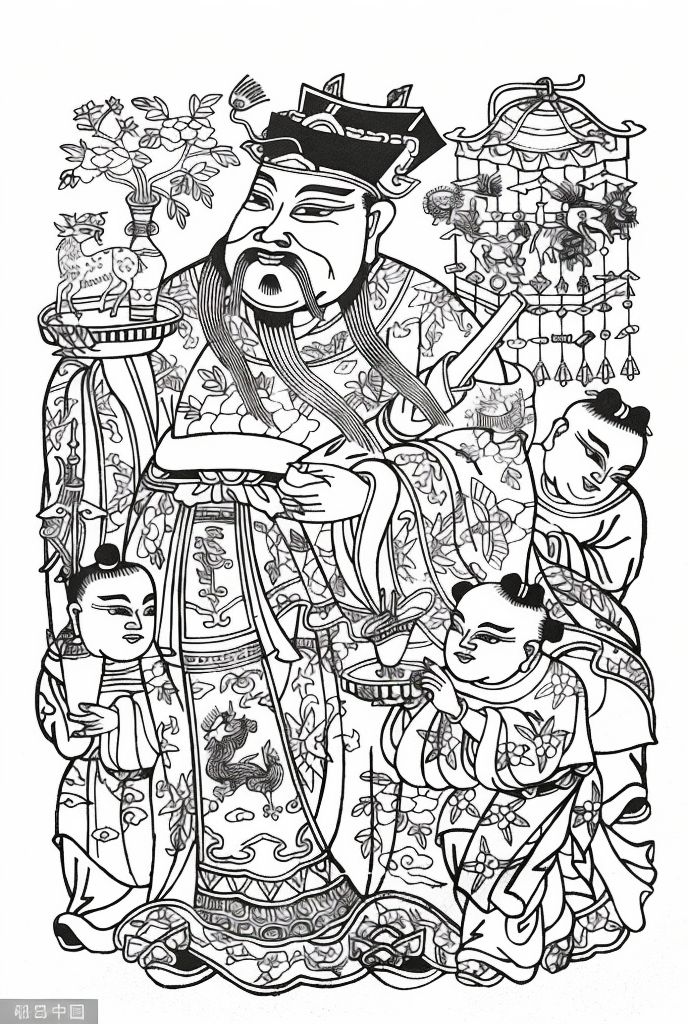  ,((((red clothes))))),black eyeliner,black beard,holding,black hair,white background,long sleeves,hat,2boys,simple background,chinese clothes,red mask:1.3,wide sleeves,black eyes,standing,1girl,((((white vase)))),((((black cup)))),((black eyeliner)),(((red flowers))),  Negative prompt: ng_deepnegative_v1_75t,(badhandv4:1.2),EasyNegative,(worst quality:2),(((negative_hand))),(((red eyeliner))),((red hand)),  Steps: 25, Size: 688x1024, Seed: 2240968183, Model: 四川绵竹年画,20250103-1735889487738-0003, Sampler: 15, CFG scale: 6.5 | 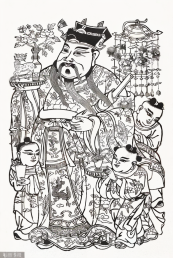  ,((((red clothes))))),black eyeliner,black beard,holding,black hair,white background,long sleeves,hat,2boys,simple background,chinese clothes,red mask:1.3,wide sleeves,black eyes,standing,1girl,((((white vase)))),((((black cup)))),((black eyeliner)),(((red flowers))),  Negative prompt: ng_deepnegative_v1_75t,(badhandv4:1.2),EasyNegative,(worst quality:2),(((negative_hand))),(((red eyeliner))),((red hand)),  Steps: 25, Size: 688x1024, Seed: 748132195, Model: 四川绵竹年画,20250103-1735889487738-0003, Sampler: 15, CFG scale: 6.5 | 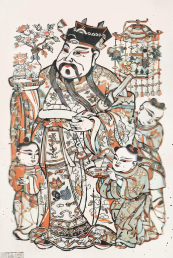  ,((((red clothes))))),black eyeliner,black beard,holding,black hair,white background,long sleeves,hat,2boys,simple background,chinese clothes,red mask:1.3,wide sleeves,black eyes,standing,1girl,((((white vase)))),((((black cup)))),((black eyeliner)),(((red flowers))),  Negative prompt: ng_deepnegative_v1_75t,(badhandv4:1.2),EasyNegative,(worst quality:2),(((negative_hand))),(((red eyeliner))),((red hand)),  Steps: 25, Size: 688x1024, Seed: 1525297788, Model: 四川绵竹年画,20250103-1735889487738-0003, Sampler: 15, CFG scale: 6.5 | 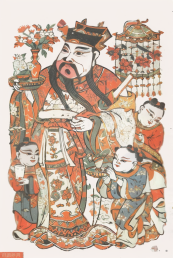  ,((((red clothes))))),black eyeliner,black beard,holding,black hair,white background,long sleeves,hat,2boys,simple background,chinese clothes,red mask:1.3,wide sleeves,black eyes,standing,1girl,((((white vase)))),((((black cup)))),((black eyeliner)),(((red flowers))),  Negative prompt: ng_deepnegative_v1_75t,(badhandv4:1.2),EasyNegative,(worst quality:2),(((negative_hand))),(((red eyeliner))),((red hand)),  Steps: 25, Size: 688x1024, Seed: 1315654546, Model: 四川绵竹年画,20250103-1735889487738-0003, Sampler: 15, CFG scale: 6.5 | 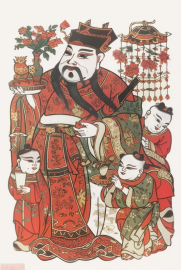,((((red clothes))))),black eyeliner,black beard,holding,black hair,white background,long sleeves,hat,2boys,simple background,chinese clothes,red mask:1.3,wide sleeves,black eyes,standing,1girl,((((white vase)))),((((black cup)))),((black eyeliner)),(((red flowers))),  Negative prompt: ng_deepnegative_v1_75t,(badhandv4:1.2),EasyNegative,(worst quality:2),(((negative_hand))),(((red eyeliner))),((red hand)),  Steps: 25, Size: 688x1024, Seed: 858461653, Model: 四川绵竹年画,20250103-1735889487738-0003, Sampler: 15, CFG scale: 6.5 |
| CFG8 | 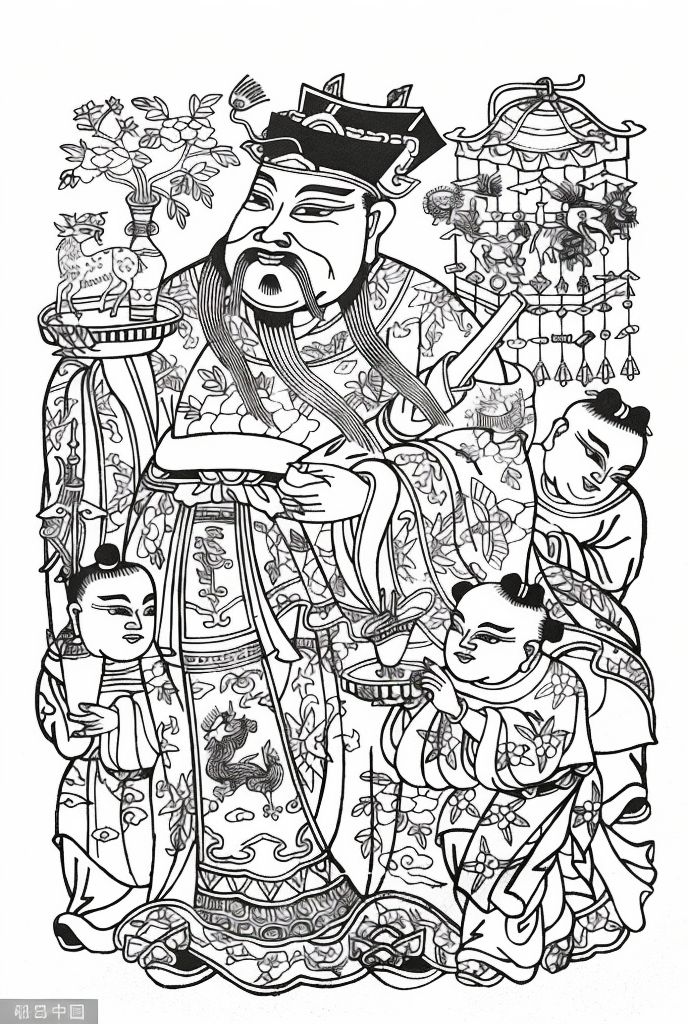  ,((((red clothes))))),black eyeliner,black beard,holding,black hair,white background,long sleeves,hat,2boys,simple background,chinese clothes,red mask:1.3,wide sleeves,black eyes,standing,1girl,((((white vase)))),((((black cup)))),((black eyeliner)),(((red flowers))),  Negative prompt: ng_deepnegative_v1_75t,(badhandv4:1.2),EasyNegative,(worst quality:2),(((negative_hand))),(((red eyeliner))),((red hand)),  Steps: 25, Size: 688x1024, Seed: 2406320252, Model: 四川绵竹年画,20250103-1735889487738-0003, Sampler: 15, CFG scale: 8 | 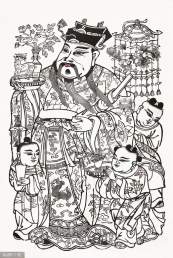  ,((((red clothes))))),black eyeliner,black beard,holding,black hair,white background,long sleeves,hat,2boys,simple background,chinese clothes,red mask:1.3,wide sleeves,black eyes,standing,1girl,((((white vase)))),((((black cup)))),((black eyeliner)),(((red flowers))),  Negative prompt: ng_deepnegative_v1_75t,(badhandv4:1.2),EasyNegative,(worst quality:2),(((negative_hand))),(((red eyeliner))),((red hand)),  Steps: 25, Size: 688x1024, Seed: 2710211718, Model: 四川绵竹年画,20250103-1735889487738-0003, Sampler: 15, CFG scale: 8 | 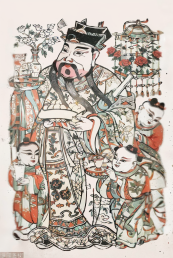  ,((((red clothes))))),black eyeliner,black beard,holding,black hair,white background,long sleeves,hat,2boys,simple background,chinese clothes,red mask:1.3,wide sleeves,black eyes,standing,1girl,((((white vase)))),((((black cup)))),((black eyeliner)),(((red flowers))),  Negative prompt: ng_deepnegative_v1_75t,(badhandv4:1.2),EasyNegative,(worst quality:2),(((negative_hand))),(((red eyeliner))),((red hand)),  Steps: 25, Size: 688x1024, Seed: 3042801021, Model: 四川绵竹年画,20250103-1735889487738-0003, Sampler: 15, CFG scale: 8 | 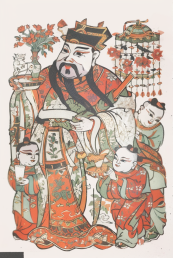  ,((((red clothes))))),black eyeliner,black beard,holding,black hair,white background,long sleeves,hat,2boys,simple background,chinese clothes,red mask:1.3,wide sleeves,black eyes,standing,1girl,((((white vase)))),((((black cup)))),((black eyeliner)),(((red flowers))),  Negative prompt: ng_deepnegative_v1_75t,(badhandv4:1.2),EasyNegative,(worst quality:2),(((negative_hand))),(((red eyeliner))),((red hand)),  Steps: 25, Size: 688x1024, Seed: 3716902206, Model: 四川绵竹年画,20250103-1735889487738-0003, Sampler: 15, CFG scale: 8 | 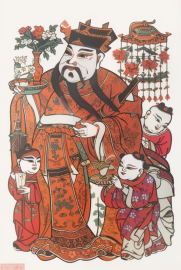,((((red clothes))))),black eyeliner,black beard,holding,black hair,white background,long sleeves,hat,2boys,simple background,chinese clothes,red mask:1.3,wide sleeves,black eyes,standing,1girl,((((white vase)))),((((black cup)))),((black eyeliner)),(((red flowers))),  Negative prompt: ng_deepnegative_v1_75t,(badhandv4:1.2),EasyNegative,(worst quality:2),(((negative_hand))),(((red eyeliner))),((red hand)),  Steps: 25, Size: 688x1024, Seed: 1895168055, Model: 四川绵竹年画,20250103-1735889487738-0003, Sampler: 15, CFG scale: 8 |
| CFG10 | 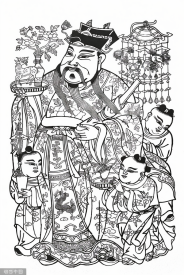  ,((((red clothes))))),black eyeliner,black beard,holding,black hair,white background,long sleeves,hat,2boys,simple background,chinese clothes,red mask:1.3,wide sleeves,black eyes,standing,1girl,((((white vase)))),((((black cup)))),((black eyeliner)),(((red flowers))),  Negative prompt: ng_deepnegative_v1_75t,(badhandv4:1.2),EasyNegative,(worst quality:2),(((negative_hand))),(((red eyeliner))),((red hand)),  Steps: 25, Size: 688x1024, Seed: 190952777, Model: 四川绵竹年画,20250103-1735889487738-0003, Sampler: 15, CFG scale: 10 | 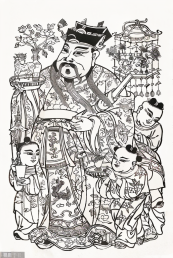  ,((((red clothes))))),black eyeliner,black beard,holding,black hair,white background,long sleeves,hat,2boys,simple background,chinese clothes,red mask:1.3,wide sleeves,black eyes,standing,1girl,((((white vase)))),((((black cup)))),((black eyeliner)),(((red flowers))),  Negative prompt: ng_deepnegative_v1_75t,(badhandv4:1.2),EasyNegative,(worst quality:2),(((negative_hand))),(((red eyeliner))),((red hand)),  Steps: 25, Size: 688x1024, Seed: 2623027433, Model: 四川绵竹年画,20250103-1735889487738-0003, Sampler: 15, CFG scale: 10 | 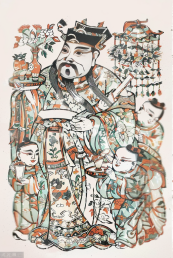  ,((((red clothes))))),black eyeliner,black beard,holding,black hair,white background,long sleeves,hat,2boys,simple background,chinese clothes,red mask:1.3,wide sleeves,black eyes,standing,1girl,((((white vase)))),((((black cup)))),((black eyeliner)),(((red flowers))),  Negative prompt: ng_deepnegative_v1_75t,(badhandv4:1.2),EasyNegative,(worst quality:2),(((negative_hand))),(((red eyeliner))),((red hand)),  Steps: 25, Size: 688x1024, Seed: 497527061, Model: 四川绵竹年画,20250103-1735889487738-0003, Sampler: 15, CFG scale: 10 | 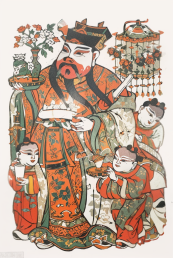  ,((((red clothes))))),black eyeliner,black beard,holding,black hair,white background,long sleeves,hat,2boys,simple background,chinese clothes,red mask:1.3,wide sleeves,black eyes,standing,1girl,((((white vase)))),((((black cup)))),((black eyeliner)),(((red flowers))),  Negative prompt: ng_deepnegative_v1_75t,(badhandv4:1.2),EasyNegative,(worst quality:2),(((negative_hand))),(((red eyeliner))),((red hand)),  Steps: 25, Size: 688x1024, Seed: 1588153726, Model: 四川绵竹年画,20250103-1735889487738-0003, Sampler: 15, CFG scale: 10 | 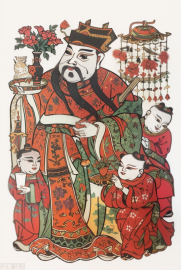  ,((((red clothes))))),black eyeliner,black beard,holding,black hair,white background,long sleeves,hat,2boys,simple background,chinese clothes,red mask:1.3,wide sleeves,black eyes,standing,1girl,((((white vase)))),((((black cup)))),((black eyeliner)),(((red flowers))),  Negative prompt: ng_deepnegative_v1_75t,(badhandv4:1.2),EasyNegative,(worst quality:2),(((negative_hand))),(((red eyeliner))),((red hand)),  Steps: 25, Size: 688x1024, Seed: 3085747129, Model: 四川绵竹年画,20250103-1735889487738-0003, Sampler: 15, CFG scale: 10 |

3、Fig 17 Text-to-image pattern series 2 generation parameters


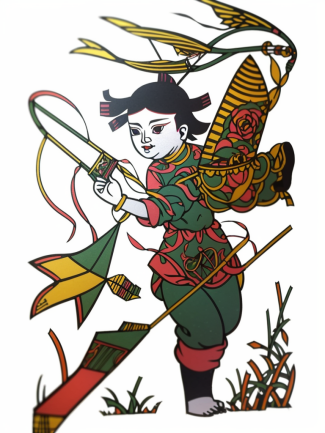

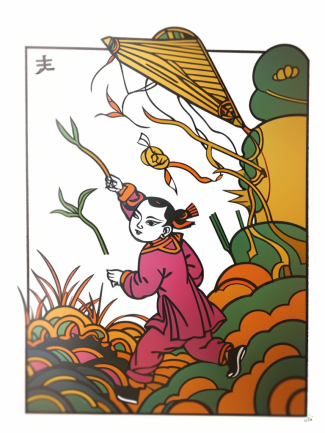

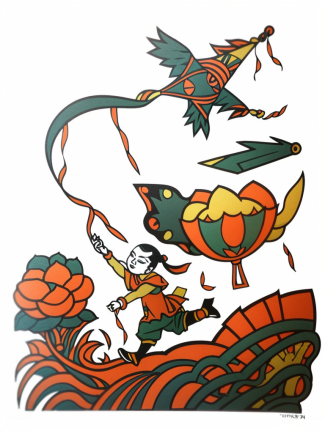


| 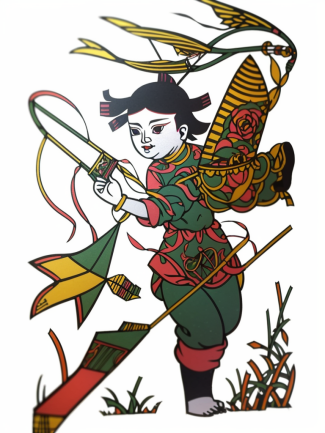 | 1 girl,((kite:1.3)),running,in spring,day,nature,full_shot,wide_shot,(turn_one's_back),  Negative prompt: ng_deepnegative_v1_75t,(badhandv4:1.2),EasyNegative,(worst quality:2),  Steps: 25,  Size: 768x1024,  Seed: 1051988756,  Model: 四川绵竹年画,20250103-1735889487738-0003,  Sampler: 15,  CFG scale: 7 |
| --- | --- |
| 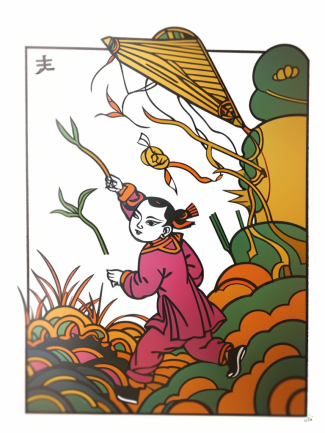 | 1 girl,((kite:1.3)),running,in spring,day,nature,full_shot,wide_shot,(turn_one's_back),  Negative prompt: ng_deepnegative_v1_75t,(badhandv4:1.2),EasyNegative,(worst quality:2),  Steps: 25,  Size: 768x1024,  Seed: 1051988756,  Model: 四川绵竹年画,20250103-1735889487738-0003,  Sampler: 15,  CFG scale: 7 |
| 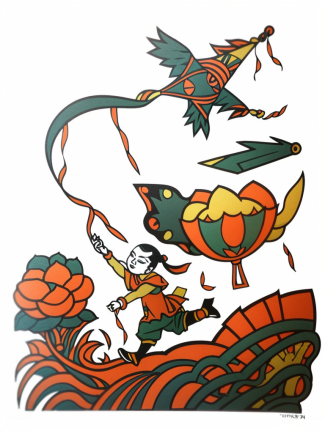 | 1 girl,((kite:1.3)),running,in spring,day,nature,full_shot,wide_shot,(turn_one's_back),  Negative prompt: ng_deepnegative_v1_75t,(badhandv4:1.2),EasyNegative,(worst quality:2),  Steps: 25,  Size: 768x1024,  Seed: 1051988756,  Model: 四川绵竹年画,20250103-1735889487738-0003,  Sampler: 15,  CFG scale: 7 |

4、Fig 18-19 Image-to-image pattern series 3 generation parameters

垫图：
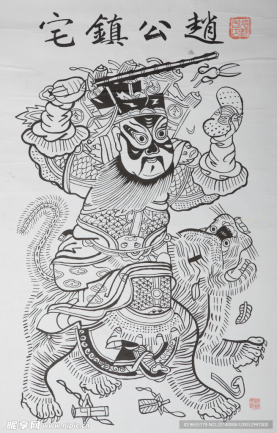


**→**
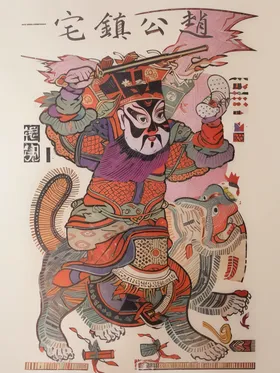

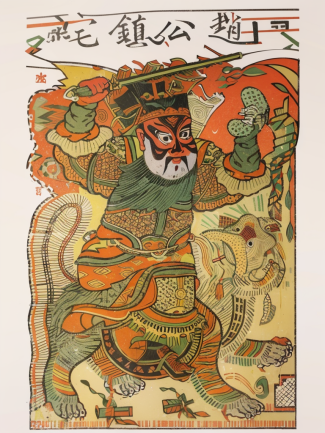

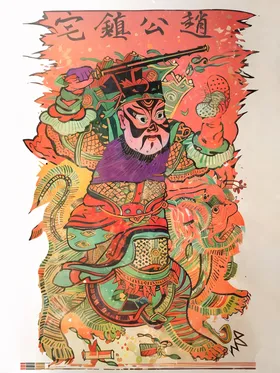


| 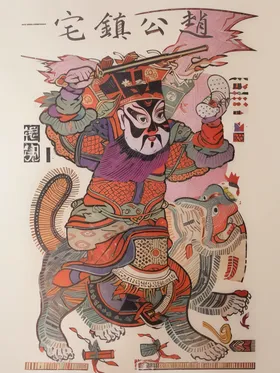 | ,Red,Purple,Black,  Negative prompt: ng_deepnegative_v1_75t,(badhandv4:1.2),EasyNegative,(worst quality:2),  Steps: 25,  Size: 768x1024,  Seed: 2315658976,  Model: 四川绵竹年画,20250103-1735889487738-0003,  Sampler: 15,  CFG scale: 7 |
| --- | --- |
| 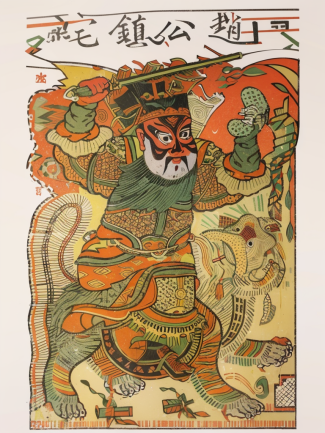 | ,Red,Purple,Black,  Negative prompt: ng_deepnegative_v1_75t,(badhandv4:1.2),EasyNegative,(worst quality:2),  Steps: 25,  Size: 768x1024,  Seed: 2315658976,  Model: 四川绵竹年画,20250103-1735889487738-0003,  Sampler: 15,  CFG scale: 7 |
| 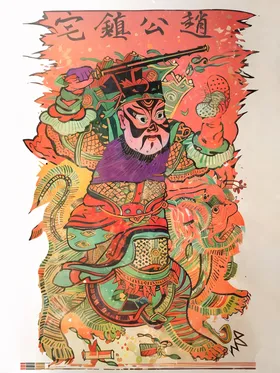 | ,Red,purple,green,  Negative prompt: ng_deepnegative_v1_75t,(badhandv4:1.2),EasyNegative,(worst quality:2),((blurry)),((lowres)),  Steps: 25,  Size: 768x1024,  Seed: 1894528264,  Model: 四川绵竹年画,20250103-1735889487738-0003,  Sampler: 15,  CFG scale: 7 |
