# Supplementary material for: Transformation of artistic style and innovative design of oriental folk patterns based on AIGC Technology—A case study of Zhuxian town new year paintings from China
Source: PLoS One. 2026 May 27;21(5):e0346020. doi: 10.1371/journal.pone.0346020 (PMC13215520; doi:10.1371/journal.pone.0346020)
Supplement: S13 Appendix — (DOCX) [file pone.0346020.s013.docx]

1. Who the recruited experts were?

R: Chang'e Kuang, Associate Professor in Art Design, School of Art, Hunan University of Science ondTechnology

Xiaotong Li, Lecturer in Art Design, Hunan University Of Technology ond BusinessZiwei Luo, Lecturer in Film and Television Animation, Xiangton Institute of Technology

Qinglei Du, Lecturer in Fine Arts, Xiangtan institute of Technology

Zizheng Liu, lecturer in Art Design, Xiangtan Institute of Technology

2. How they were recruited, what criteria did they need to meet, what was the exclusion criteria?

R: The specific recruitment process and criteria are as follows:

Recruitment channel: 5 experts are selected from the members of China Arts and Crafts Association, and the dual-track system of “organization recommendation + qualification review” is adopted.

Admission criteria (to be met simultaneously): ≥10 years of professional experience in the field, participation in the evaluation of national yearbook exhibitions, and no commercial interest in AIGC technology.

Exclusion Criteria (eliminated if any of them are met): participation in the development of AI painting tools in the past 3 years, academic cooperation with research team members, age > 70 years old.

3. Are these external to the the study?

R: All experts are independent of the study team:

R: All experts are independent of the research team: they have not participated in the design or training of the AIGC model for the study, they have signed the Declaration of External Experts prior to the review, and they have not received any financial benefits related to the study.

4. How consent was documented to take part?

R: Electronic process through double authentication:

Step 1: Send the Informed Consent Form containing the study description to the expert's email address and complete the signature.

Step 2: Sign the Consent Authorization Form and keep the paper signed document at the same time.

5. When you recruited these individuals (day/month/year)

R: Specifically as follows:

First contact: October 1, 2024 (initial communication by email)

Formal Confirmation: Complete signing of all agreements by December 13, 2024

First contact: October 1, 2024 (initial communication by email)

Formal confirmation: Signature of all agreements by December 13, 2024

6.What information the experts were provided with prior to engaging with the study and how consent was recorded.

R: Specify the following:

Information provided: Summary of the purpose of the study (in Chinese and English), Evaluation Indicator Specification (50 AIGC-generated images with different redrawing magnitudes and keyword guidance coefficients), Authorization for Consent

Record of consent: signed document of Informed Consent, signed document of Authorization for Consent
