# Supplementary material for: Transformation of artistic style and innovative design of oriental folk patterns based on AIGC Technology—A case study of Zhuxian town new year paintings from China
Source: PLoS One. 2026 May 27;21(5):e0346020. doi: 10.1371/journal.pone.0346020 (PMC13215520; doi:10.1371/journal.pone.0346020)
Supplement: S14 Appendix — (PDF) [file pone.0346020.s014.pdf]

Expert 1

Redraw amplitude / CFG	Redraw amplitude 0	Redraw amplitude 0.3	Redraw amplitude 0.5	Redraw amplitude 0.7	Redraw amplitude 1.0
CFG=2	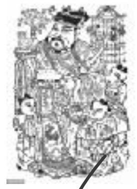<br>① □1 □2 □3 □4 □5 □6 □7<br>② □1 □2 □3 □4 □5 □6 □7<br>③ □1 □2 □3 □4 □5 □6 □7<br>④ □1 □2 □3 □4 □5 □6 □7<br>⑤ □1 □2 □3 □4 □5 □6 □7	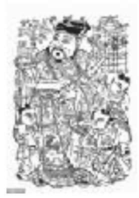<br>① □1 □2 □3 □4 □5 □6 □7<br>② □1 □2 □3 □4 □5 □6 □7<br>③ □1 □2 □3 □4 □5 □6 □7<br>④ □1 □2 □3 □4 □5 □6 □7<br>⑤ □1 □2 □3 □4 □5 □6 □7	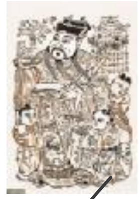<br>① □1 □2 □3 □4 □5 □6 □7<br>② □1 □2 □3 □4 □5 □6 □7<br>③ □1 □2 □3 □4 □5 □6 □7<br>④ □1 □2 □3 □4 □5 □6 □7<br>⑤ □1 □2 □3 □4 □5 □6 □7	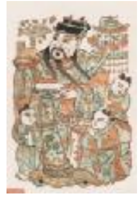<br>① □1 □2 □3 □4 □5 □6 □7<br>② □1 □2 □3 □4 □5 □6 □7<br>③ □1 □2 □3 □4 □5 □6 □7<br>④ □1 □2 □3 □4 □5 □6 □7<br>⑤ □1 □2 □3 □4 □5 □6 □7	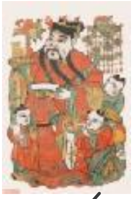<br>① □1 □2 □3 □4 □5 □6 □7<br>② □1 □2 □3 □4 □5 □6 □7<br>③ □1 □2 □3 □4 □5 □6 □7<br>④ □1 □2 □3 □4 □5 □6 □7<br>⑤ □1 □2 □3 □4 □5 □6 □7
CFG=5	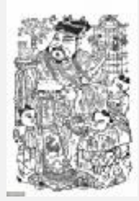<br>① □1 □2 □3 □4 □5 □6 □7<br>② □1 □2 □3 □4 □5 □6 □7<br>③ □1 □2 □3 □4 □5 □6 □7<br>④ □1 □2 □3 □4 □5 □6 □7<br>⑤ □1 □2 □3 □4 □5 □6 □7	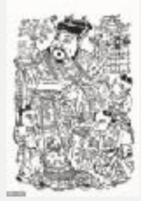<br>① □1 □2 □3 □4 □5 □6 □7<br>② □1 □2 □3 □4 □5 □6 □7<br>③ □1 □2 □3 □4 □5 □6 □7<br>④ □1 □2 □3 □4 □5 □6 □7<br>⑤ □1 □2 □3 □4 □5 □6 □7	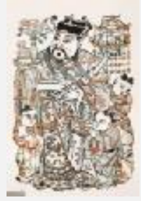<br>① □1 □2 □3 □4 □5 □6 □7<br>② □1 □2 □3 □4 □5 □6 □7<br>③ □1 □2 □3 □4 □5 □6 □7<br>④ □1 □2 □3 □4 □5 □6 □7<br>⑤ □1 □2 □3 □4 □5 □6 □7	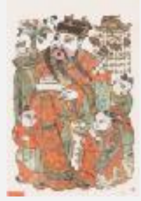<br>① □1 □2 □3 □4 □5 □6 □7<br>② □1 □2 □3 □4 □5 □6 □7<br>③ □1 □2 □3 □4 □5 □6 □7<br>④ □1 □2 □3 □4 □5 □6 □7<br>⑤ □1 □2 □3 □4 □5 □6 □7	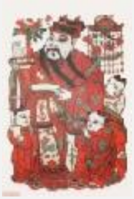<br>① □1 □2 □3 □4 □5 □6 □7<br>② □1 □2 □3 □4 □5 □6 □7<br>③ □1 □2 □3 □4 □5 □6 □7<br>④ □1 □2 □3 □4 □5 □6 □7<br>⑤ □1 □2 □3 □4 □5 □6 □7
CFG=6.5	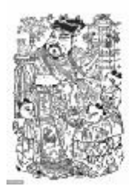<br>① □1 □2 □3 □4 □5 □6 □7<br>② □1 □2 □3 □4 □5 □6 □7<br>③ □1 □2 □3 □4 □5 □6 □7<br>④ □1 □2 □3 □4 □5 □6 □7<br>⑤ □1 □2 □3 □4 □5 □6 □7	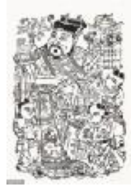<br>① □1 □2 □3 □4 □5 □6 □7<br>② □1 □2 □3 □4 □5 □6 □7<br>③ □1 □2 □3 □4 □5 □6 □7<br>④ □1 □2 □3 □4 □5 □6 □7<br>⑤ □1 □2 □3 □4 □5 □6 □7	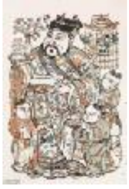<br>① □1 □2 □3 □4 □5 □6 □7<br>② □1 □2 □3 □4 □5 □6 □7<br>③ □1 □2 □3 □4 □5 □6 □7<br>④ □1 □2 □3 □4 □5 □6 □7<br>⑤ □1 □2 □3 □4 □5 □6 □7	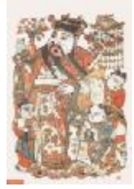<br>① □1 □2 □3 □4 □5 □6 □7<br>② □1 □2 □3 □4 □5 □6 □7<br>③ □1 □2 □3 □4 □5 □6 □7<br>④ □1 □2 □3 □4 □5 □6 □7<br>⑤ □1 □2 □3 □4 □5 □6 □7	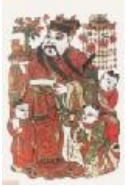<br>① □1 □2 □3 □4 □5 □6 □7<br>② □1 □2 □3 □4 □5 □6 □7<br>③ □1 □2 □3 □4 □5 □6 □7<br>④ □1 □2 □3 □4 □5 □6 □7<br>⑤ □1 □2 □3 □4 □5 □6 □7

CFG=8	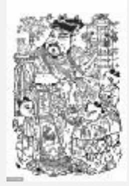<br>① □1 □2 □3 □4 □5 ☑6 □7<br>② ☑1 □2 □3 □4 □5 □6 □7<br>③ □1 ☑2 □3 □4 □5 □6 □7<br>④ □1 ☑2 □3 □4 □5 □6 □7<br>⑤ □1 □2 □3 □4 ☑5 □6 □7	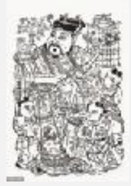<br>① □1 □2 □3 ☑4 □5 □6 □7<br>② □1 ☑2 □3 □4 □5 □6 □7<br>③ ☑1 □2 □3 □4 □5 □6 □7<br>④ □1 □2 □3 ☑4 □5 □6 □7<br>⑤ □1 □2 □3 □4 □5 ☑6 □7	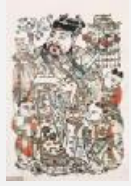<br>① □1 □2 □3 □4 ☑5 □6 □7<br>② □1 □2 ☑3 □4 □5 □6 □7<br>③ □1 □2 ☑3 □4 □5 □6 □7<br>④ □1 □2 □3 □4 □5 ☑6 □7<br>⑤ □1 □2 □3 ☑4 □5 □6 □7	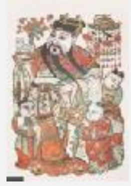<br>① □1 □2 □3 ☑4 □5 □6 □7<br>② □1 □2 ☑3 □4 □5 □6 □7<br>③ □1 □2 □3 ☑4 □5 □6 □7<br>④ □1 □2 □3 ☑4 □5 □6 □7<br>⑤ □1 □2 □3 □4 ☑5 □6 □7	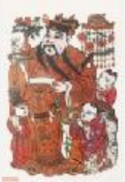<br>① □1 □2 □3 □4 ☑5 □6 □7<br>② □1 □2 □3 ☑4 □5 □6 □7<br>③ □1 □2 □3 □4 □5 ☑6 □7<br>④ ☑1 □2 □3 □4 □5 □6 □7<br>⑤ □1 □2 ☑3 □4 □5 □6 □7
CFG=10	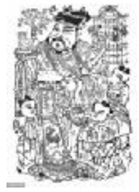<br>① □1 □2 □3 □4 □5 □6 ☑7<br>② □1 ☑2 □3 □4 □5 □6 □7<br>③ ☑1 □2 □3 □4 □5 □6 □7<br>④ □1 □2 ☑3 □4 □5 □6 □7<br>⑤ □1 ☑2 □3 □4 □5 □6 □7	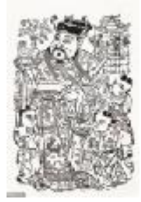<br>① □1 □2 □3 □4 □5 ☑6 □7<br>② □1 ☑2 □3 □4 □5 □6 □7<br>③ ☑1 □2 □3 □4 □5 □6 □7<br>④ □1 □2 □3 □4 ☑5 □6 □7<br>⑤ □1 □2 □3 ☑4 □5 □6 □7	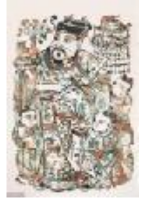<br>① □1 □2 □3 □4 □5 ☑6 □7<br>② □1 □2 ☑3 □4 □5 □6 □7<br>③ □1 □2 □3 □4 ☑5 □6 □7<br>④ □1 □2 □3 □4 □5 ☑6 □7<br>⑤ □1 □2 ☑3 □4 □5 □6 □7	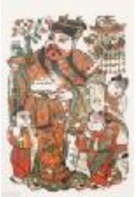<br>① □1 □2 □3 □4 ☑5 □6 □7<br>② □1 □2 ☑3 □4 □5 □6 □7<br>③ □1 □2 ☑3 □4 □5 □6 □7<br>④ □1 □2 ☑3 □4 □5 □6 □7<br>⑤ □1 ☑2 □3 □4 □5 □6 □7	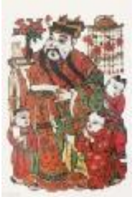<br>① □1 □2 □3 □4 ☑5 □6 □7<br>② □1 □2 □3 ☑4 □5 □6 □7<br>③ □1 □2 □3 ☑4 □5 □6 □7<br>④ □1 ☑2 □3 □4 □5 □6 □7<br>⑤ ☑1 □2 □3 □4 □5 □6 □7

Expert 1

CFG \ Redraw amplitude	Redraw amplitude 0	Redraw amplitude 0.3	Redraw amplitude 0.5	Redraw amplitude 0.7	Redraw amplitude 1.0
CFG=2	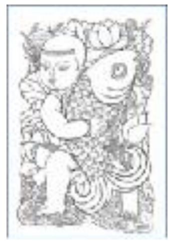	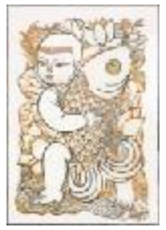	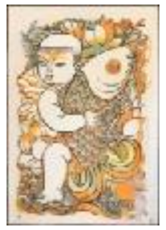	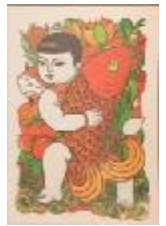	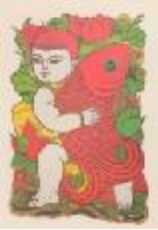
	① □1 □2 □3 □4 □5 □6 □7	① □1 □2 □3 □4 □5 □6 □7	① □1 □2 □3 □4 □5 □6 □7	① □1 □2 □3 □4 □5 □6 □7	① □1 □2 □3 □4 □5 □6 □7
	② □1 □2 □3 □4 □5 □6 □7	② □1 □2 □3 □4 □5 □6 □7	② □1 □2 □3 □4 □5 □6 □7	② □1 □2 □3 □4 □5 □6 □7	② □1 □2 □3 □4 □5 □6 □7
	③ □1 □2 □3 □4 □5 □6 □7	③ □1 □2 □3 □4 □5 □6 □7	③ □1 □2 □3 □4 □5 □6 □7	③ □1 □2 □3 □4 □5 □6 □7	③ □1 □2 □3 □4 □5 □6 □7
	④ □1 □2 □3 □4 □5 □6 □7	④ □1 □2 □3 □4 □5 □6 □7	④ □1 □2 □3 □4 □5 □6 □7	④ □1 □2 □3 □4 □5 □6 □7	④ □1 □2 □3 □4 □5 □6 □7
	⑤ □1 □2 □3 □4 □5 □6 □7	⑤ □1 □2 □3 □4 □5 □6 □7	⑤ □1 □2 □3 □4 □5 □6 □7	⑤ □1 □2 □3 □4 □5 □6 □7	⑤ □1 □2 □3 □4 □5 □6 □7
CFG=5	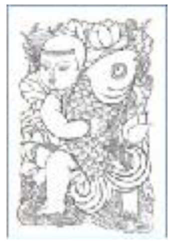	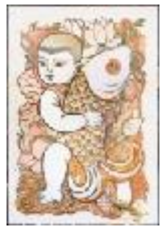	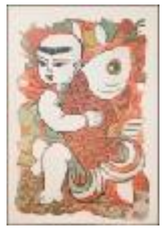	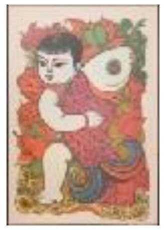	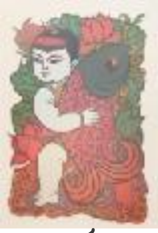
	① □1 □2 □3 □4 □5 □6 □7	① □1 □2 □3 □4 □5 □6 □7	① □1 □2 □3 □4 □5 □6 □7	① □1 □2 □3 □4 □5 □6 □7	① □1 □2 □3 □4 □5 □6 □7
	② □1 □2 □3 □4 □5 □6 □7	② □1 □2 □3 □4 □5 □6 □7	② □1 □2 □3 □4 □5 □6 □7	② □1 □2 □3 □4 □5 □6 □7	② □1 □2 □3 □4 □5 □6 □7
	③ □1 □2 □3 □4 □5 □6 □7	③ □1 □2 □3 □4 □5 □6 □7	③ □1 □2 □3 □4 □5 □6 □7	③ □1 □2 □3 □4 □5 □6 □7	③ □1 □2 □3 □4 □5 □6 □7
	④ □1 □2 □3 □4 □5 □6 □7	④ □1 □2 □3 □4 □5 □6 □7	④ □1 □2 □3 □4 □5 □6 □7	④ □1 □2 □3 □4 □5 □6 □7	④ □1 □2 □3 □4 □5 □6 □7
	⑤ □1 □2 □3 □4 □5 □6 □7	⑤ □1 □2 □3 □4 □5 □6 □7	⑤ □1 □2 □3 □4 □5 □6 □7	⑤ □1 □2 □3 □4 □5 □6 □7	⑤ □1 □2 □3 □4 □5 □6 □7
CFG=6.5	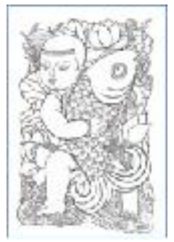	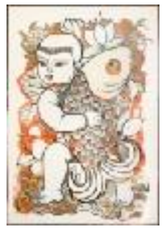	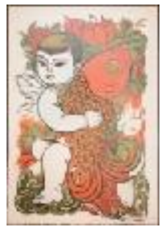	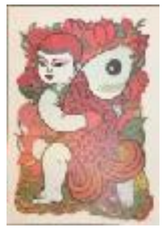	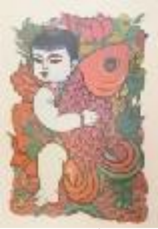
	① □1 □2 □3 □4 □5 □6 □7	① □1 □2 □3 □4 □5 □6 □7	① □1 □2 □3 □4 □5 □6 □7	① □1 □2 □3 □4 □5 □6 □7	① □1 □2 □3 □4 □5 □6 □7
	② □1 □2 □3 □4 □5 □6 □7	② □1 □2 □3 □4 □5 □6 □7	② □1 □2 □3 □4 □5 □6 □7	② □1 □2 □3 □4 □5 □6 □7	② □1 □2 □3 □4 □5 □6 □7
	③ □1 □2 □3 □4 □5 □6 □7	③ □1 □2 □3 □4 □5 □6 □7	③ □1 □2 □3 □4 □5 □6 □7	③ □1 □2 □3 □4 □5 □6 □7	③ □1 □2 □3 □4 □5 □6 □7
	④ □1 □2 □3 □4 □5 □6 □7	④ □1 □2 □3 □4 □5 □6 □7	④ □1 □2 □3 □4 □5 □6 □7	④ □1 □2 □3 □4 □5 □6 □7	④ □1 □2 □3 □4 □5 □6 □7
	⑤ □1 □2 □3 □4 □5 □6 □7	⑤ □1 □2 □3 □4 □5 □6 □7	⑤ □1 □2 □3 □4 □5 □6 □7	⑤ □1 □2 □3 □4 □5 □6 □7	⑤ □1 □2 □3 □4 □5 □6 □7

	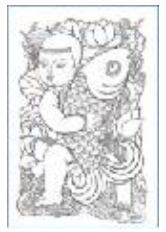	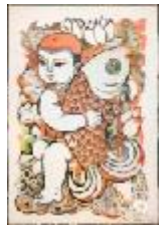	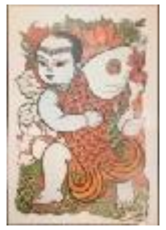	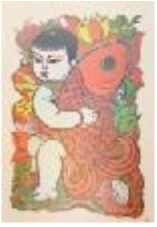	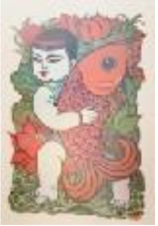
CFG=8	① □1 □2 □3 □4 □5 □6 □7<br>② □1 □2 □3 □4 □5 □6 □7<br>③ □1 □2 □3 □4 □5 □6 □7<br>④ □1 □2 □3 □4 □5 □6 □7<br>⑤ □1 □2 □3 □4 □5 □6 □7	① □1 □2 □3 □4 □5 □6 □7<br>② □1 □2 □3 □4 □5 □6 □7<br>③ □1 □2 □3 □4 □5 □6 □7<br>④ □1 □2 □3 □4 □5 □6 □7<br>⑤ □1 □2 □3 □4 □5 □6 □7	① □1 □2 □3 □4 □5 □6 □7<br>② □1 □2 □3 □4 □5 □6 □7<br>③ □1 □2 □3 □4 □5 □6 □7<br>④ □1 □2 □3 □4 □5 □6 □7<br>⑤ □1 □2 □3 □4 □5 □6 □7	① □1 □2 □3 □4 □5 □6 □7<br>② □1 □2 □3 □4 □5 □6 □7<br>③ □1 □2 □3 □4 □5 □6 □7<br>④ □1 □2 □3 □4 □5 □6 □7<br>⑤ □1 □2 □3 □4 □5 □6 □7	① □1 □2 □3 □4 □5 □6 □7<br>② □1 □2 □3 □4 □5 □6 □7<br>③ □1 □2 □3 □4 □5 □6 □7<br>④ □1 □2 □3 □4 □5 □6 □7<br>⑤ □1 □2 □3 □4 □5 □6 □7
	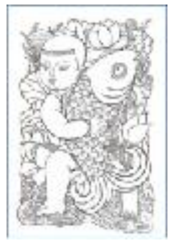	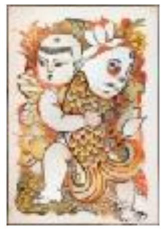	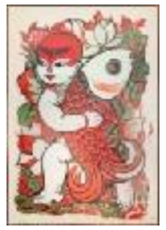	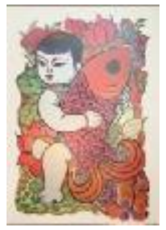	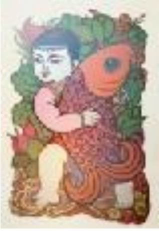
CFG=10	① □1 □2 □3 □4 □5 □6 □7<br>② □1 □2 □3 □4 □5 □6 □7<br>③ □1 □2 □3 □4 □5 □6 □7<br>④ □1 □2 □3 □4 □5 □6 □7<br>⑤ □1 □2 □3 □4 □5 □6 □7	① □1 □2 □3 □4 □5 □6 □7<br>② □1 □2 □3 □4 □5 □6 □7<br>③ □1 □2 □3 □4 □5 □6 □7<br>④ □1 □2 □3 □4 □5 □6 □7<br>⑤ □1 □2 □3 □4 □5 □6 □7	① □1 □2 □3 □4 □5 □6 □7<br>② □1 □2 □3 □4 □5 □6 □7<br>③ □1 □2 □3 □4 □5 □6 □7<br>④ □1 □2 □3 □4 □5 □6 □7<br>⑤ □1 □2 □3 □4 □5 □6 □7	① □1 □2 □3 □4 □5 □6 □7<br>② □1 □2 □3 □4 □5 □6 □7<br>③ □1 □2 □3 □4 □5 □6 □7<br>④ □1 □2 □3 □4 □5 □6 □7<br>⑤ □1 □2 □3 □4 □5 □6 □7	① □1 □2 □3 □4 □5 □6 □7<br>② □1 □2 □3 □4 □5 □6 □7<br>③ □1 □2 □3 □4 □5 □6 □7<br>④ □1 □2 □3 □4 □5 □6 □7<br>⑤ □1 □2 □3 □4 □5 □6 □7

Expert 2

Redraw amplitude / CFG	Redraw amplitude 0	Redraw amplitude 0.3	Redraw amplitude 0.5	Redraw amplitude 0.7	Redraw amplitude 1.0
CFG=2	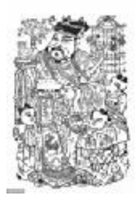<br>① □1 □2 □3 □4 □5 □6 □7<br>② □1 □2 □3 □4 □5 □6 □7<br>③ □1 □2 □3 □4 □5 □6 □7<br>④ □1 □2 □3 □4 □5 □6 □7<br>⑤ □1 □2 □3 □4 □5 □6 □7	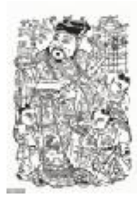<br>① □1 □2 □3 □4 □5 □6 □7<br>② □1 □2 □3 □4 □5 □6 □7<br>③ □1 □2 □3 □4 □5 □6 □7<br>④ □1 □2 □3 □4 □5 □6 □7<br>⑤ □1 □2 □3 □4 □5 □6 □7	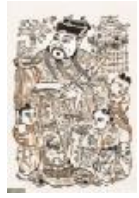<br>① □1 □2 □3 □4 □5 □6 □7<br>② □1 □2 □3 □4 □5 □6 □7<br>③ □1 □2 □3 □4 □5 □6 □7<br>④ □1 □2 □3 □4 □5 □6 □7<br>⑤ □1 □2 □3 □4 □5 □6 □7	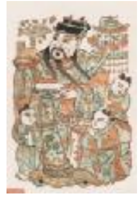<br>① □1 □2 □3 □4 □5 □6 □7<br>② □1 □2 □3 □4 □5 □6 □7<br>③ □1 □2 □3 □4 □5 □6 □7<br>④ □1 □2 □3 □4 □5 □6 □7<br>⑤ □1 □2 □3 □4 □5 □6 □7	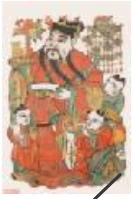<br>① □1 □2 □3 □4 □5 □6 □7<br>② □1 □2 □3 □4 □5 □6 □7<br>③ □1 □2 □3 □4 □5 □6 □7<br>④ □1 □2 □3 □4 □5 □6 □7<br>⑤ □1 □2 □3 □4 □5 □6 □7
CFG=5	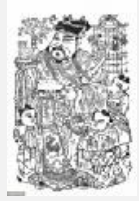<br>① □1 □2 □3 □4 □5 □6 □7<br>② □1 □2 □3 □4 □5 □6 □7<br>③ □1 □2 □3 □4 □5 □6 □7<br>④ □1 □2 □3 □4 □5 □6 □7<br>⑤ □1 □2 □3 □4 □5 □6 □7	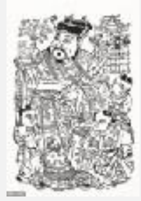<br>① □1 □2 □3 □4 □5 □6 □7<br>② □1 □2 □3 □4 □5 □6 □7<br>③ □1 □2 □3 □4 □5 □6 □7<br>④ □1 □2 □3 □4 □5 □6 □7<br>⑤ □1 □2 □3 □4 □5 □6 □7	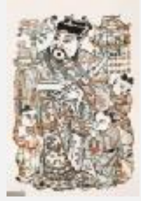<br>① □1 □2 □3 □4 □5 □6 □7<br>② □1 □2 □3 □4 □5 □6 □7<br>③ □1 □2 □3 □4 □5 □6 □7<br>④ □1 □2 □3 □4 □5 □6 □7<br>⑤ □1 □2 □3 □4 □5 □6 □7	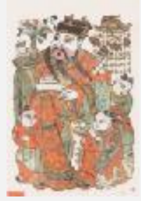<br>① □1 □2 □3 □4 □5 □6 □7<br>② □1 □2 □3 □4 □5 □6 □7<br>③ □1 □2 □3 □4 □5 □6 □7<br>④ □1 □2 □3 □4 □5 □6 □7<br>⑤ □1 □2 □3 □4 □5 □6 □7	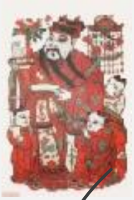<br>① □1 □2 □3 □4 □5 □6 □7<br>② □1 □2 □3 □4 □5 □6 □7<br>③ □1 □2 □3 □4 □5 □6 □7<br>④ □1 □2 □3 □4 □5 □6 □7<br>⑤ □1 □2 □3 □4 □5 □6 □7
CFG=6.5	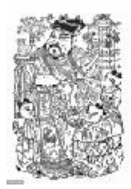<br>① □1 □2 □3 □4 □5 □6 □7<br>② □1 □2 □3 □4 □5 □6 □7<br>③ □1 □2 □3 □4 □5 □6 □7<br>④ □1 □2 □3 □4 □5 □6 □7<br>⑤ □1 □2 □3 □4 □5 □6 □7	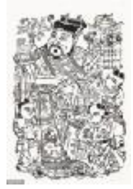<br>① □1 □2 □3 □4 □5 □6 □7<br>② □1 □2 □3 □4 □5 □6 □7<br>③ □1 □2 □3 □4 □5 □6 □7<br>④ □1 □2 □3 □4 □5 □6 □7<br>⑤ □1 □2 □3 □4 □5 □6 □7	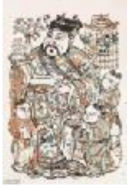<br>① □1 □2 □3 □4 □5 □6 □7<br>② □1 □2 □3 □4 □5 □6 □7<br>③ □1 □2 □3 □4 □5 □6 □7<br>④ □1 □2 □3 □4 □5 □6 □7<br>⑤ □1 □2 □3 □4 □5 □6 □7	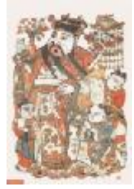<br>① □1 □2 □3 □4 □5 □6 □7<br>② □1 □2 □3 □4 □5 □6 □7<br>③ □1 □2 □3 □4 □5 □6 □7<br>④ □1 □2 □3 □4 □5 □6 □7<br>⑤ □1 □2 □3 □4 □5 □6 □7	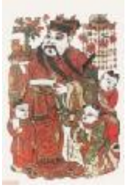<br>① □1 □2 □3 □4 □5 □6 □7<br>② □1 □2 □3 □4 □5 □6 □7<br>③ □1 □2 □3 □4 □5 □6 □7<br>④ □1 □2 □3 □4 □5 □6 □7<br>⑤ □1 □2 □3 □4 □5 □6 □7

CFG=8	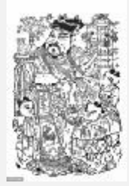<br>① □1 □2 □3 □4 □5 □6 □7<br>② □1 □2 □3 □4 □5 □6 □7<br>③ □1 □2 □3 □4 □5 □6 □7<br>④ □1 □2 □3 □4 □5 □6 □7<br>⑤ □1 □2 □3 □4 □5 □6 □7	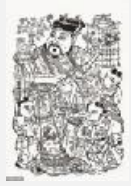<br>① □1 □2 □3 □4 □5 □6 □7<br>② □1 □2 □3 □4 □5 □6 □7<br>③ □1 □2 □3 □4 □5 □6 □7<br>④ □1 □2 □3 □4 □5 □6 □7<br>⑤ □1 □2 □3 □4 □5 □6 □7	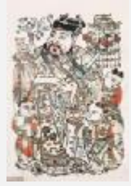<br>① □1 □2 □3 □4 □5 □6 □7<br>② □1 □2 □3 □4 □5 □6 □7<br>③ □1 □2 □3 □4 □5 □6 □7<br>④ □1 □2 □3 □4 □5 □6 □7<br>⑤ □1 □2 □3 □4 □5 □6 □7	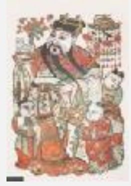<br>① □1 □2 □3 □4 □5 □6 □7<br>② □1 □2 □3 □4 □5 □6 □7<br>③ □1 □2 □3 □4 □5 □6 □7<br>④ □1 □2 □3 □4 □5 □6 □7<br>⑤ □1 □2 □3 □4 □5 □6 □7	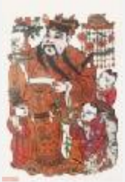<br>① □1 □2 □3 □4 □5 □6 □7<br>② □1 □2 □3 □4 □5 □6 □7<br>③ □1 □2 □3 □4 □5 □6 □7<br>④ □1 □2 □3 □4 □5 □6 □7<br>⑤ □1 □2 □3 □4 □5 □6 □7
CFG=10	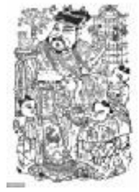<br>① □1 □2 □3 □4 □5 □6 □7<br>② □1 □2 □3 □4 □5 □6 □7<br>③ □1 □2 □3 □4 □5 □6 □7<br>④ □1 □2 □3 □4 □5 □6 □7<br>⑤ □1 □2 □3 □4 □5 □6 □7	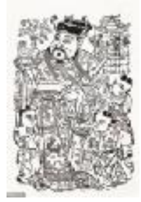<br>① □1 □2 □3 □4 □5 □6 □7<br>② □1 □2 □3 □4 □5 □6 □7<br>③ □1 □2 □3 □4 □5 □6 □7<br>④ □1 □2 □3 □4 □5 □6 □7<br>⑤ □1 □2 □3 □4 □5 □6 □7	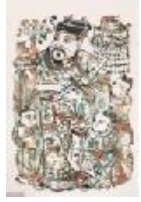<br>① □1 □2 □3 □4 □5 □6 □7<br>② □1 □2 □3 □4 □5 □6 □7<br>③ □1 □2 □3 □4 □5 □6 □7<br>④ □1 □2 □3 □4 □5 □6 □7<br>⑤ □1 □2 □3 □4 □5 □6 □7	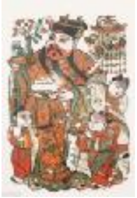<br>① □1 □2 □3 □4 □5 □6 □7<br>② □1 □2 □3 □4 □5 □6 □7<br>③ □1 □2 □3 □4 □5 □6 □7<br>④ □1 □2 □3 □4 □5 □6 □7<br>⑤ □1 □2 □3 □4 □5 □6 □7	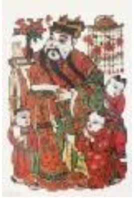<br>① □1 □2 □3 □4 □5 □6 □7<br>② □1 □2 □3 □4 □5 □6 □7<br>③ □1 □2 □3 □4 □5 □6 □7<br>④ □1 □2 □3 □4 □5 □6 □7<br>⑤ □1 □2 □3 □4 □5 □6 □7

Expert 2

Redraw amplitude / CFG	Redraw amplitude 0	Redraw amplitude 0.3	Redraw amplitude 0.5	Redraw amplitude 0.7	Redraw amplitude 1.0
CFG=2	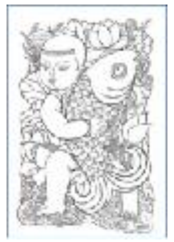<br>① □1 □2 □3 □4 □5 □6 □7<br>② □1 □2 □3 □4 □5 □6 □7<br>③ □1 □2 □3 □4 □5 □6 □7<br>④ □1 □2 □3 □4 □5 □6 □7<br>⑤ □1 □2 □3 □4 □5 □6 □7	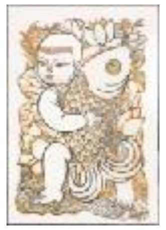<br>① □1 □2 □3 □4 □5 □6 □7<br>② □1 □2 □3 □4 □5 □6 □7<br>③ □1 □2 □3 □4 □5 □6 □7<br>④ □1 □2 □3 □4 □5 □6 □7<br>⑤ □1 □2 □3 □4 □5 □6 □7	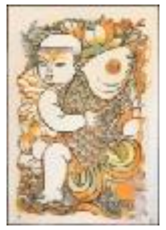<br>① □1 □2 □3 □4 □5 □6 □7<br>② □1 □2 □3 □4 □5 □6 □7<br>③ □1 □2 □3 □4 □5 □6 □7<br>④ □1 □2 □3 □4 □5 □6 □7<br>⑤ □1 □2 □3 □4 □5 □6 □7	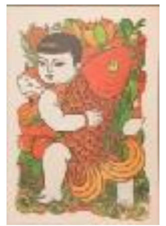<br>① □1 □2 □3 □4 □5 □6 □7<br>② □1 □2 □3 □4 □5 □6 □7<br>③ □1 □2 □3 □4 □5 □6 □7<br>④ □1 □2 □3 □4 □5 □6 □7<br>⑤ □1 □2 □3 □4 □5 □6 □7	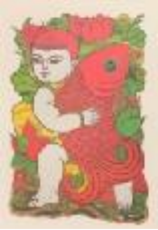<br>① □1 □2 □3 □4 □5 □6 □7<br>② □1 □2 □3 □4 □5 □6 □7<br>③ □1 □2 □3 □4 □5 □6 □7<br>④ □1 □2 □3 □4 □5 □6 □7<br>⑤ □1 □2 □3 □4 □5 □6 □7
CFG=5	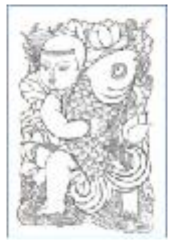<br>① □1 □2 □3 □4 □5 □6 □7<br>② □1 □2 □3 □4 □5 □6 □7<br>③ □1 □2 □3 □4 □5 □6 □7<br>④ □1 □2 □3 □4 □5 □6 □7<br>⑤ □1 □2 □3 □4 □5 □6 □7	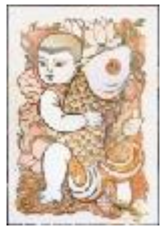<br>① □1 □2 □3 □4 □5 □6 □7<br>② □1 □2 □3 □4 □5 □6 □7<br>③ □1 □2 □3 □4 □5 □6 □7<br>④ □1 □2 □3 □4 □5 □6 □7<br>⑤ □1 □2 □3 □4 □5 □6 □7	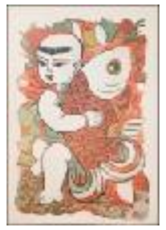<br>① □1 □2 □3 □4 □5 □6 □7<br>② □1 □2 □3 □4 □5 □6 □7<br>③ □1 □2 □3 □4 □5 □6 □7<br>④ □1 □2 □3 □4 □5 □6 □7<br>⑤ □1 □2 □3 □4 □5 □6 □7	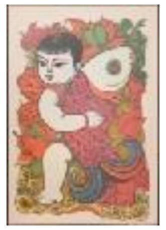<br>① □1 □2 □3 □4 □5 □6 □7<br>② □1 □2 □3 □4 □5 □6 □7<br>③ □1 □2 □3 □4 □5 □6 □7<br>④ □1 □2 □3 □4 □5 □6 □7<br>⑤ □1 □2 □3 □4 □5 □6 □7	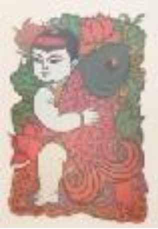<br>① □1 □2 □3 □4 □5 □6 □7<br>② □1 □2 □3 □4 □5 □6 □7<br>③ □1 □2 □3 □4 □5 □6 □7<br>④ □1 □2 □3 □4 □5 □6 □7<br>⑤ □1 □2 □3 □4 □5 □6 □7
CFG=6.5	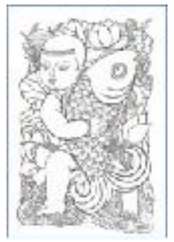<br>① □1 □2 □3 □4 □5 □6 □7<br>② □1 □2 □3 □4 □5 □6 □7<br>③ □1 □2 □3 □4 □5 □6 □7<br>④ □1 □2 □3 □4 □5 □6 □7<br>⑤ □1 □2 □3 □4 □5 □6 □7	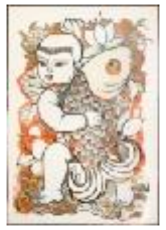<br>① □1 □2 □3 □4 □5 □6 □7<br>② □1 □2 □3 □4 □5 □6 □7<br>③ □1 □2 □3 □4 □5 □6 □7<br>④ □1 □2 □3 □4 □5 □6 □7<br>⑤ □1 □2 □3 □4 □5 □6 □7	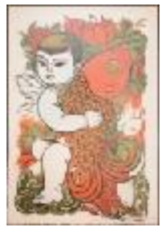<br>① □1 □2 □3 □4 □5 □6 □7<br>② □1 □2 □3 □4 □5 □6 □7<br>③ □1 □2 □3 □4 □5 □6 □7<br>④ □1 □2 □3 □4 □5 □6 □7<br>⑤ □1 □2 □3 □4 □5 □6 □7	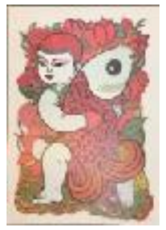<br>① □1 □2 □3 □4 □5 □6 □7<br>② □1 □2 □3 □4 □5 □6 □7<br>③ □1 □2 □3 □4 □5 □6 □7<br>④ □1 □2 □3 □4 □5 □6 □7<br>⑤ □1 □2 □3 □4 □5 □6 □7	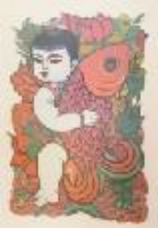<br>① □1 □2 □3 □4 □5 □6 □7<br>② □1 □2 □3 □4 □5 □6 □7<br>③ □1 □2 □3 □4 □5 □6 □7<br>④ □1 □2 □3 □4 □5 □6 □7<br>⑤ □1 □2 □3 □4 □5 □6 □7

	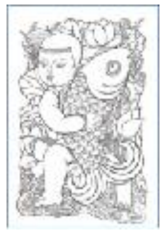	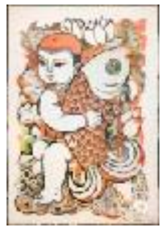	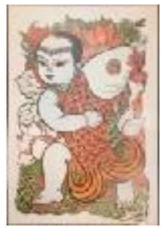	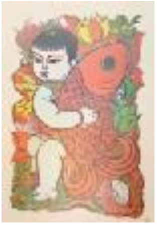	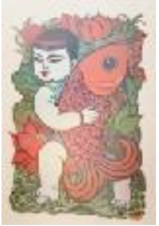
CFG=8	① □1 □2 □3 □4 □5 □6 □7	① □1 □2 □3 □4 □5 □6 □7	① □1 □2 □3 □4 □5 □6 □7	① □1 □2 □3 □4 □5 □6 □7	① □1 □2 □3 □4 □5 □6 □7
	② □1 □2 □3 □4 □5 □6 □7	② □1 □2 □3 □4 □5 □6 □7	② □1 □2 □3 □4 □5 □6 □7	② □1 □2 □3 □4 □5 □6 □7	② □1 □2 □3 □4 □5 □6 □7
	③ □1 □2 □3 □4 □5 □6 □7	③ □1 □2 □3 □4 □5 □6 □7	③ □1 □2 □3 □4 □5 □6 □7	③ □1 □2 □3 □4 □5 □6 □7	③ □1 □2 □3 □4 □5 □6 □7
	④ □1 □2 □3 □4 □5 □6 □7	④ □1 □2 □3 □4 □5 □6 □7	④ □1 □2 □3 □4 □5 □6 □7	④ □1 □2 □3 □4 □5 □6 □7	④ □1 □2 □3 □4 □5 □6 □7
	⑤ □1 □2 □3 □4 □5 □6 □7	⑤ □1 □2 □3 □4 □5 □6 □7	⑤ □1 □2 □3 □4 □5 □6 □7	⑤ □1 □2 □3 □4 □5 □6 □7	⑤ □1 □2 □3 □4 □5 □6 □7
	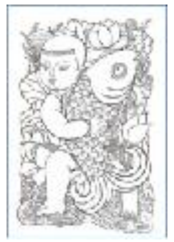	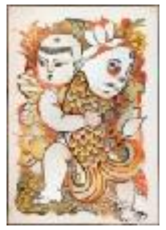	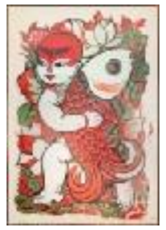	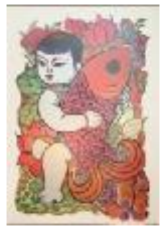	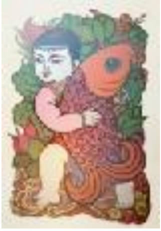
CFG=10	① □1 □2 □3 □4 □5 □6 □7	① □1 □2 □3 □4 □5 □6 □7	① □1 □2 □3 □4 □5 □6 □7	① □1 □2 □3 □4 □5 □6 □7	① □1 □2 □3 □4 □5 □6 □7
	② □1 □2 □3 □4 □5 □6 □7	② □1 □2 □3 □4 □5 □6 □7	② □1 □2 □3 □4 □5 □6 □7	② □1 □2 □3 □4 □5 □6 □7	② □1 □2 □3 □4 □5 □6 □7
	③ □1 □2 □3 □4 □5 □6 □7	③ □1 □2 □3 □4 □5 □6 □7	③ □1 □2 □3 □4 □5 □6 □7	③ □1 □2 □3 □4 □5 □6 □7	③ □1 □2 □3 □4 □5 □6 □7
	④ □1 □2 □3 □4 □5 □6 □7	④ □1 □2 □3 □4 □5 □6 □7	④ □1 □2 □3 □4 □5 □6 □7	④ □1 □2 □3 □4 □5 □6 □7	④ □1 □2 □3 □4 □5 □6 □7
	⑤ □1 □2 □3 □4 □5 □6 □7	⑤ □1 □2 □3 □4 □5 □6 □7	⑤ □1 □2 □3 □4 □5 □6 □7	⑤ □1 □2 □3 □4 □5 □6 □7	⑤ □1 □2 □3 □4 □5 □6 □7

Expert 3

Redraw amplitude / CFG	Redraw amplitude 0	Redraw amplitude 0.3	Redraw amplitude 0.5	Redraw amplitude 0.7	Redraw amplitude 1.0
CFG=2	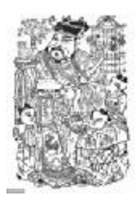<br>① □1 □2 □3 □4 □5 □6 □7<br>② □1 □2 □3 □4 □5 □6 □7<br>③ □1 □2 □3 □4 □5 □6 □7<br>④ □1 □2 □3 □4 □5 □6 □7<br>⑤ □1 □2 □3 □4 □5 □6 □7	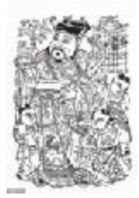<br>① □1 □2 □3 □4 □5 □6 □7<br>② □1 □2 □3 □4 □5 □6 □7<br>③ □1 □2 □3 □4 □5 □6 □7<br>④ □1 □2 □3 □4 □5 □6 □7<br>⑤ □1 □2 □3 □4 □5 □6 □7	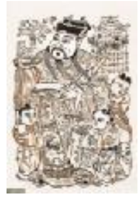<br>① □1 □2 □3 □4 □5 □6 □7<br>② □1 □2 □3 □4 □5 □6 □7<br>③ □1 □2 □3 □4 □5 □6 □7<br>④ □1 □2 □3 □4 □5 □6 □7<br>⑤ □1 □2 □3 □4 □5 □6 □7	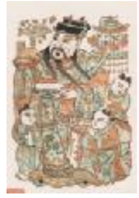<br>① □1 □2 □3 □4 □5 □6 □7<br>② □1 □2 □3 □4 □5 □6 □7<br>③ □1 □2 □3 □4 □5 □6 □7<br>④ □1 □2 □3 □4 □5 □6 □7<br>⑤ □1 □2 □3 □4 □5 □6 □7	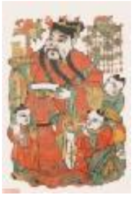<br>① □1 □2 □3 □4 □5 □6 □7<br>② □1 □2 □3 □4 □5 □6 □7<br>③ □1 □2 □3 □4 □5 □6 □7<br>④ □1 □2 □3 □4 □5 □6 □7<br>⑤ □1 □2 □3 □4 □5 □6 □7
CFG=5	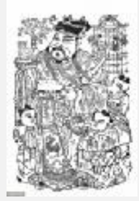<br>① □1 □2 □3 □4 □5 □6 □7<br>② □1 □2 □3 □4 □5 □6 □7<br>③ □1 □2 □3 □4 □5 □6 □7<br>④ □1 □2 □3 □4 □5 □6 □7<br>⑤ □1 □2 □3 □4 □5 □6 □7	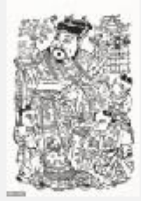<br>① □1 □2 □3 □4 □5 □6 □7<br>② □1 □2 □3 □4 □5 □6 □7<br>③ □1 □2 □3 □4 □5 □6 □7<br>④ □1 □2 □3 □4 □5 □6 □7<br>⑤ □1 □2 □3 □4 □5 □6 □7	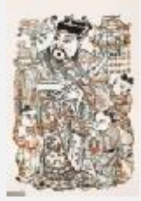<br>① □1 □2 □3 □4 □5 □6 □7<br>② □1 □2 □3 □4 □5 □6 □7<br>③ □1 □2 □3 □4 □5 □6 □7<br>④ □1 □2 □3 □4 □5 □6 □7<br>⑤ □1 □2 □3 □4 □5 □6 □7	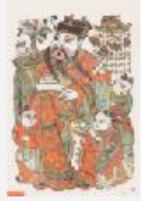<br>① □1 □2 □3 □4 □5 □6 □7<br>② □1 □2 □3 □4 □5 □6 □7<br>③ □1 □2 □3 □4 □5 □6 □7<br>④ □1 □2 □3 □4 □5 □6 □7<br>⑤ □1 □2 □3 □4 □5 □6 □7	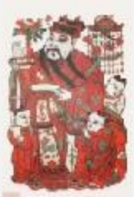<br>① □1 □2 □3 □4 □5 □6 □7<br>② □1 □2 □3 □4 □5 □6 □7<br>③ □1 □2 □3 □4 □5 □6 □7<br>④ □1 □2 □3 □4 □5 □6 □7<br>⑤ □1 □2 □3 □4 □5 □6 □7
CFG=6.5	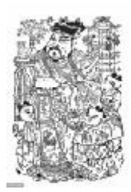<br>① □1 □2 □3 □4 □5 □6 □7<br>② □1 □2 □3 □4 □5 □6 □7<br>③ □1 □2 □3 □4 □5 □6 □7<br>④ □1 □2 □3 □4 □5 □6 □7<br>⑤ □1 □2 □3 □4 □5 □6 □7	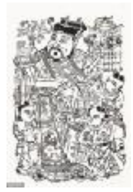<br>① □1 □2 □3 □4 □5 □6 □7<br>② □1 □2 □3 □4 □5 □6 □7<br>③ □1 □2 □3 □4 □5 □6 □7<br>④ □1 □2 □3 □4 □5 □6 □7<br>⑤ □1 □2 □3 □4 □5 □6 □7	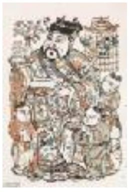<br>① □1 □2 □3 □4 □5 □6 □7<br>② □1 □2 □3 □4 □5 □6 □7<br>③ □1 □2 □3 □4 □5 □6 □7<br>④ □1 □2 □3 □4 □5 □6 □7<br>⑤ □1 □2 □3 □4 □5 □6 □7	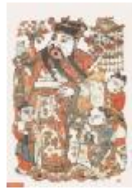<br>① □1 □2 □3 □4 □5 □6 □7<br>② □1 □2 □3 □4 □5 □6 □7<br>③ □1 □2 □3 □4 □5 □6 □7<br>④ □1 □2 □3 □4 □5 □6 □7<br>⑤ □1 □2 □3 □4 □5 □6 □7	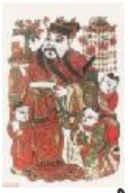<br>① □1 □2 □3 □4 □5 □6 □7<br>② □1 □2 □3 □4 □5 □6 □7<br>③ □1 □2 □3 □4 □5 □6 □7<br>④ □1 □2 □3 □4 □5 □6 □7<br>⑤ □1 □2 □3 □4 □5 □6 □7

	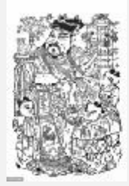	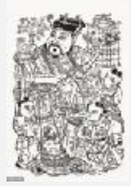	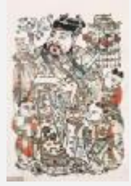	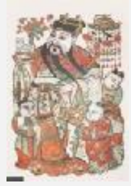	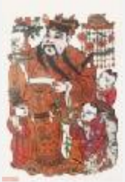
CFG=8	① □1 □2 □3 □4 □5 □6 □7<br>② □1 □2 □3 □4 □5 □6 □7<br>③ □1 □2 □3 □4 □5 □6 □7<br>④ □1 □2 □3 □4 □5 □6 □7<br>⑤ □1 □2 □3 □4 □5 □6 □7	① □1 □2 □3 □4 □5 □6 □7<br>② □1 □2 □3 □4 □5 □6 □7<br>③ □1 □2 □3 □4 □5 □6 □7<br>④ □1 □2 □3 □4 □5 □6 □7<br>⑤ □1 □2 □3 □4 □5 □6 □7	① □1 □2 □3 □4 □5 □6 □7<br>② □1 □2 □3 □4 □5 □6 □7<br>③ □1 □2 □3 □4 □5 □6 □7<br>④ □1 □2 □3 □4 □5 □6 □7<br>⑤ □1 □2 □3 □4 □5 □6 □7	① □1 □2 □3 □4 □5 □6 □7<br>② □1 □2 □3 □4 □5 □6 □7<br>③ □1 □2 □3 □4 □5 □6 □7<br>④ □1 □2 □3 □4 □5 □6 □7<br>⑤ □1 □2 □3 □4 □5 □6 □7	① □1 □2 □3 □4 □5 □6 □7<br>② □1 □2 □3 □4 □5 □6 □7<br>③ □1 □2 □3 □4 □5 □6 □7<br>④ □1 □2 □3 □4 □5 □6 □7<br>⑤ □1 □2 □3 □4 □5 □6 □7
	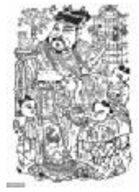	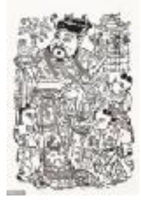	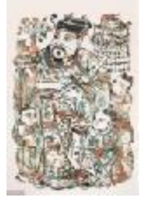	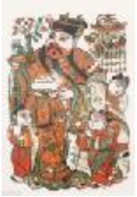	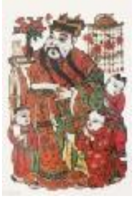
CFG=10	① □1 □2 □3 □4 □5 □6 □7<br>② □1 □2 □3 □4 □5 □6 □7<br>③ □1 □2 □3 □4 □5 □6 □7<br>④ □1 □2 □3 □4 □5 □6 □7<br>⑤ □1 □2 □3 □4 □5 □6 □7	① □1 □2 □3 □4 □5 □6 □7<br>② □1 □2 □3 □4 □5 □6 □7<br>③ □1 □2 □3 □4 □5 □6 □7<br>④ □1 □2 □3 □4 □5 □6 □7<br>⑤ □1 □2 □3 □4 □5 □6 □7	① □1 □2 □3 □4 □5 □6 □7<br>② □1 □2 □3 □4 □5 □6 □7<br>③ □1 □2 □3 □4 □5 □6 □7<br>④ □1 □2 □3 □4 □5 □6 □7<br>⑤ □1 □2 □3 □4 □5 □6 □7	① □1 □2 □3 □4 □5 □6 □7<br>② □1 □2 □3 □4 □5 □6 □7<br>③ □1 □2 □3 □4 □5 □6 □7<br>④ □1 □2 □3 □4 □5 □6 □7<br>⑤ □1 □2 □3 □4 □5 □6 □7	① □1 □2 □3 □4 □5 □6 □7<br>② □1 □2 □3 □4 □5 □6 □7<br>③ □1 □2 □3 □4 □5 □6 □7<br>④ □1 □2 □3 □4 □5 □6 □7<br>⑤ □1 □2 □3 □4 □5 □6 □7

Expert 3

CFG \ Redraw amplitude	Redraw amplitude 0	Redraw amplitude **0.3**	Redraw amplitude **0.5**	Redraw amplitude **0.7**	Redraw amplitude **1.0**
CFG=2	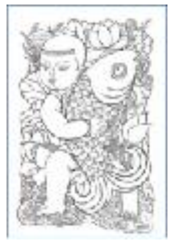<br>① □1 □2 □3 □4 □5 □6 □7<br>② □1 □2 □3 □4 □5 □6 □7<br>③ □1 □2 □3 □4 □5 □6 □7<br>④ □1 □2 □3 □4 □5 □6 □7<br>⑤ □1 □2 □3 □4 □5 □6 □7	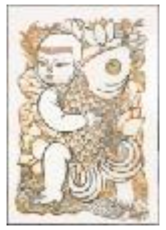<br>① □1 □2 □3 □4 □5 □6 □7<br>② □1 □2 □3 □4 □5 □6 □7<br>③ □1 □2 □3 □4 □5 □6 □7<br>④ □1 □2 □3 □4 □5 □6 □7<br>⑤ □1 □2 □3 □4 □5 □6 □7	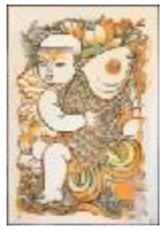<br>① □1 □2 □3 □4 □5 □6 □7<br>② □1 □2 □3 □4 □5 □6 □7<br>③ □1 □2 □3 □4 □5 □6 □7<br>④ □1 □2 □3 □4 □5 □6 □7<br>⑤ □1 □2 □3 □4 □5 □6 □7	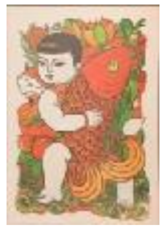<br>① □1 □2 □3 □4 □5 □6 □7<br>② □1 □2 □3 □4 □5 □6 □7<br>③ □1 □2 □3 □4 □5 □6 □7<br>④ □1 □2 □3 □4 □5 □6 □7<br>⑤ □1 □2 □3 □4 □5 □6 □7	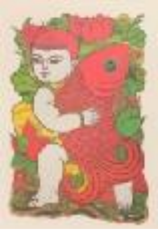<br>① □1 □2 □3 □4 □5 □6 □7<br>② □1 □2 □3 □4 □5 □6 □7<br>③ □1 □2 □3 □4 □5 □6 □7<br>④ □1 □2 □3 □4 □5 □6 □7<br>⑤ □1 □2 □3 □4 □5 □6 □7
CFG=5	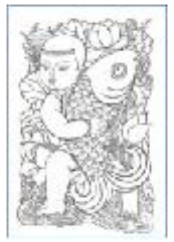<br>① □1 □2 □3 □4 □5 □6 □7<br>② □1 □2 □3 □4 □5 □6 □7<br>③ □1 □2 □3 □4 □5 □6 □7<br>④ □1 □2 □3 □4 □5 □6 □7<br>⑤ □1 □2 □3 □4 □5 □6 □7	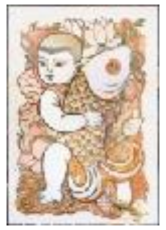<br>① □1 □2 □3 □4 □5 □6 □7<br>② □1 □2 □3 □4 □5 □6 □7<br>③ □1 □2 □3 □4 □5 □6 □7<br>④ □1 □2 □3 □4 □5 □6 □7<br>⑤ □1 □2 □3 □4 □5 □6 □7	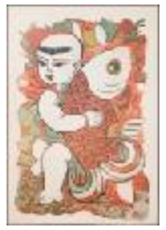<br>① □1 □2 □3 □4 □5 □6 □7<br>② □1 □2 □3 □4 □5 □6 □7<br>③ □1 □2 □3 □4 □5 □6 □7<br>④ □1 □2 □3 □4 □5 □6 □7<br>⑤ □1 □2 □3 □4 □5 □6 □7	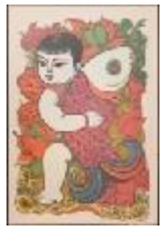<br>① □1 □2 □3 □4 □5 □6 □7<br>② □1 □2 □3 □4 □5 □6 □7<br>③ □1 □2 □3 □4 □5 □6 □7<br>④ □1 □2 □3 □4 □5 □6 □7<br>⑤ □1 □2 □3 □4 □5 □6 □7	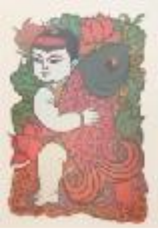<br>① □1 □2 □3 □4 □5 □6 □7<br>② □1 □2 □3 □4 □5 □6 □7<br>③ □1 □2 □3 □4 □5 □6 □7<br>④ □1 □2 □3 □4 □5 □6 □7<br>⑤ □1 □2 □3 □4 □5 □6 □7
CFG=6.5	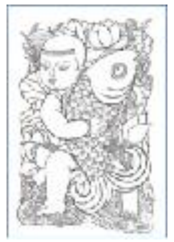<br>① □1 □2 □3 □4 □5 □6 □7<br>② □1 □2 □3 □4 □5 □6 □7<br>③ □1 □2 □3 □4 □5 □6 □7<br>④ □1 □2 □3 □4 □5 □6 □7<br>⑤ □1 □2 □3 □4 □5 □6 □7	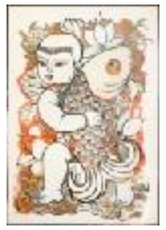<br>① □1 □2 □3 □4 □5 □6 □7<br>② □1 □2 □3 □4 □5 □6 □7<br>③ □1 □2 □3 □4 □5 □6 □7<br>④ □1 □2 □3 □4 □5 □6 □7<br>⑤ □1 □2 □3 □4 □5 □6 □7	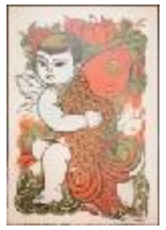<br>① □1 □2 □3 □4 □5 □6 □7<br>② □1 □2 □3 □4 □5 □6 □7<br>③ □1 □2 □3 □4 □5 □6 □7<br>④ □1 □2 □3 □4 □5 □6 □7<br>⑤ □1 □2 □3 □4 □5 □6 □7	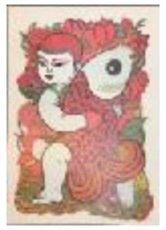<br>① □1 □2 □3 □4 □5 □6 □7<br>② □1 □2 □3 □4 □5 □6 □7<br>③ □1 □2 □3 □4 □5 □6 □7<br>④ □1 □2 □3 □4 □5 □6 □7<br>⑤ □1 □2 □3 □4 □5 □6 □7	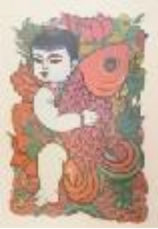<br>① □1 □2 □3 □4 □5 □6 □7<br>② □1 □2 □3 □4 □5 □6 □7<br>③ □1 □2 □3 □4 □5 □6 □7<br>④ □1 □2 □3 □4 □5 □6 □7<br>⑤ □1 □2 □3 □4 □5 □6 □7

	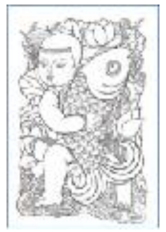	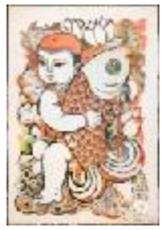	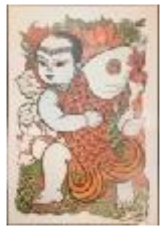	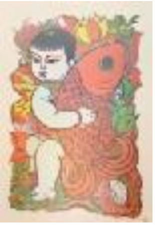	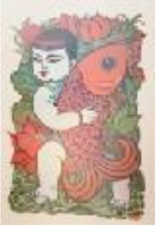
CFG=8	① □1 □2 □3 □4 □5 □6 □7	① □1 □2 □3 □4 □5 □6 □7	① □1 □2 □3 □4 □5 □6 □7	① □1 □2 □3 □4 □5 □6 □7	① □1 □2 □3 □4 □5 □6 □7
	② □1 □2 □3 □4 □5 □6 □7	② □1 □2 □3 □4 □5 □6 □7	② □1 □2 □3 □4 □5 □6 □7	② □1 □2 □3 □4 □5 □6 □7	② □1 □2 □3 □4 □5 □6 □7
	③ □1 □2 □3 □4 □5 □6 □7	③ □1 □2 □3 □4 □5 □6 □7	③ □1 □2 □3 □4 □5 □6 □7	③ □1 □2 □3 □4 □5 □6 □7	③ □1 □2 □3 □4 □5 □6 □7
	④ □1 □2 □3 □4 □5 □6 □7	④ □1 □2 □3 □4 □5 □6 □7	④ □1 □2 □3 □4 □5 □6 □7	④ □1 □2 □3 □4 □5 □6 □7	④ □1 □2 □3 □4 □5 □6 □7
	⑤ □1 □2 □3 □4 □5 □6 □7	⑤ □1 □2 □3 □4 □5 □6 □7	⑤ □1 □2 □3 □4 □5 □6 □7	⑤ □1 □2 □3 □4 □5 □6 □7	⑤ □1 □2 □3 □4 □5 □6 □7
	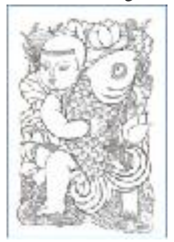	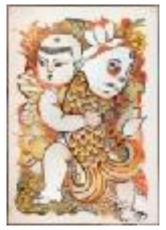	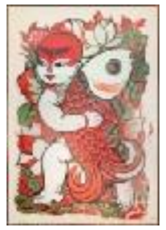	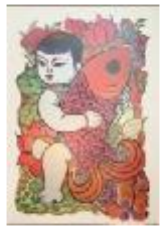	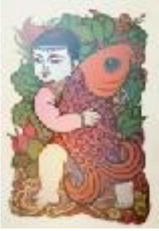
CFG=10	① □1 □2 □3 □4 □5 □6 □7	① □1 □2 □3 □4 □5 □6 □7	① □1 □2 □3 □4 □5 □6 □7	① □1 □2 □3 □4 □5 □6 □7	① □1 □2 □3 □4 □5 □6 □7
	② □1 □2 □3 □4 □5 □6 □7	② □1 □2 □3 □4 □5 □6 □7	② □1 □2 □3 □4 □5 □6 □7	② □1 □2 □3 □4 □5 □6 □7	② □1 □2 □3 □4 □5 □6 □7
	③ □1 □2 □3 □4 □5 □6 □7	③ □1 □2 □3 □4 □5 □6 □7	③ □1 □2 □3 □4 □5 □6 □7	③ □1 □2 □3 □4 □5 □6 □7	③ □1 □2 □3 □4 □5 □6 □7
	④ □1 □2 □3 □4 □5 □6 □7	④ □1 □2 □3 □4 □5 □6 □7	④ □1 □2 □3 □4 □5 □6 □7	④ □1 □2 □3 □4 □5 □6 □7	④ □1 □2 □3 □4 □5 □6 □7
	⑤ □1 □2 □3 □4 □5 □6 □7	⑤ □1 □2 □3 □4 □5 □6 □7	⑤ □1 □2 □3 □4 □5 □6 □7	⑤ □1 □2 □3 □4 □5 □6 □7	⑤ □1 □2 □3 □4 □5 □6 □7

Expert 4

CFG \ Redraw amplitude	Redraw amplitude 0	Redraw amplitude 0.3	Redraw amplitude 0.5	Redraw amplitude 0.7	Redraw amplitude 1.0
CFG=2	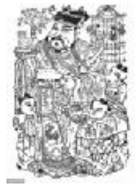<br>① □1 □2 □3 ☑4 □5 □6 □7<br>② □1 ☑2 □3 □4 □5 □6 □7<br>③ □1 ☑2 □3 □4 □5 □6 □7<br>④ ☑1 □2 □3 □4 □5 □6 □7<br>⑤ ☑1 □2 □3 □4 □5 □6 □7	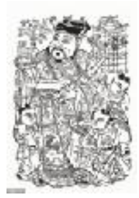<br>① □1 □2 □3 □4 ☑5 □6 □7<br>② □1 □2 ☑3 □4 □5 □6 □7<br>③ □1 □2 ☑3 □4 □5 □6 □7<br>④ □1 □2 ☑3 □4 □5 □6 □7<br>⑤ □1 □2 ☑3 □4 □5 □6 □7	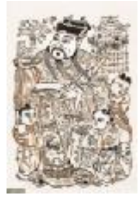<br>① □1 □2 ☑3 □4 □5 □6 □7<br>② □1 □2 □3 ☑4 □5 □6 □7<br>③ □1 □2 □3 ☑4 □5 □6 □7<br>④ □1 □2 □3 □4 ☑5 □6 □7<br>⑤ □1 □2 □3 ☑4 □5 □6 □7	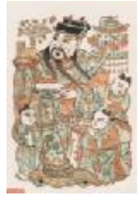<br>① □1 ☑2 □3 □4 □5 □6 □7<br>② □1 □2 □3 □4 □5 ☑6 □7<br>③ □1 □2 □3 □4 ☑5 □6 □7<br>④ □1 □2 □3 □4 ☑5 □6 □7<br>⑤ □1 □2 □3 □4 ☑5 □6 □7	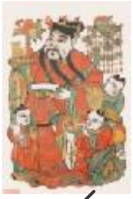<br>① ☑1 □2 □3 □4 □5 □6 □7<br>② □1 □2 □3 □4 □5 □6 ☑7<br>③ □1 □2 □3 □4 ☑5 □6 □7<br>④ □1 □2 ☑3 □4 □5 □6 □7<br>⑤ □1 □2 □3 □4 ☑5 □6 □7
CFG=5	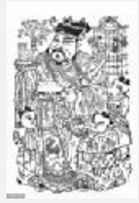<br>① □1 □2 □3 ☑4 □5 □6 □7<br>② □1 ☑2 □3 □4 □5 □6 □7<br>③ ☑1 □2 □3 □4 □5 □6 □7<br>④ □1 ☑2 □3 □4 □5 □6 □7<br>⑤ □1 ☑2 □3 □4 □5 □6 □7	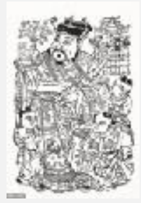<br>① □1 □2 □3 □4 ☑5 □6 □7<br>② □1 □2 ☑3 □4 □5 □6 □7<br>③ □1 ☑2 □3 □4 □5 □6 □7<br>④ □1 □2 □3 ☑4 □5 □6 □7<br>⑤ □1 □2 ☑3 □4 □5 □6 □7	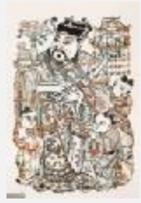<br>① □1 □2 ☑3 □4 □5 □6 □7<br>② □1 □2 □3 ☑4 □5 □6 □7<br>③ □1 □2 ☑3 □4 □5 □6 □7<br>④ □1 □2 □3 □4 □5 ☑6 □7<br>⑤ □1 □2 □3 □4 ☑5 □6 □7	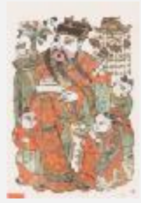<br>① □1 ☑2 □3 □4 □5 □6 □7<br>② □1 □2 □3 □4 □5 ☑6 □7<br>③ □1 □2 □3 □4 ☑5 □6 □7<br>④ □1 □2 □3 □4 ☑5 □6 □7<br>⑤ □1 □2 □3 □4 ☑5 □6 □7	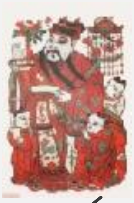<br>① ☑1 □2 □3 □4 □5 □6 □7<br>② □1 □2 □3 □4 ☑5 □6 □7<br>③ □1 □2 □3 □4 □5 ☑6 □7<br>④ □1 □2 □3 ☑4 □5 □6 □7<br>⑤ □1 □2 □3 □4 ☑5 □6 □7
CFG=6.5	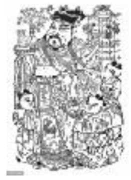<br>① □1 □2 □3 ☑4 □5 □6 □7<br>② □1 ☑2 □3 □4 □5 □6 □7<br>③ □1 ☑2 □3 □4 □5 □6 □7<br>④ ☑1 □2 □3 □4 □5 □6 □7<br>⑤ □1 ☑2 □3 □4 □5 □6 □7	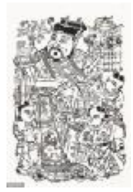<br>① □1 □2 □3 ☑4 □5 □6 □7<br>② □1 □2 ☑3 □4 □5 □6 □7<br>③ □1 ☑2 □3 □4 □5 □6 □7<br>④ □1 □2 ☑3 □4 □5 □6 □7<br>⑤ □1 ☑2 □3 □4 □5 □6 □7	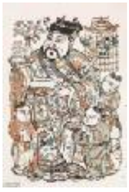<br>① □1 □2 □3 ☑4 □5 □6 □7<br>② □1 □2 □3 ☑4 □5 □6 □7<br>③ □1 □2 ☑3 □4 □5 □6 □7<br>④ □1 □2 □3 □4 ☑5 □6 □7<br>⑤ □1 □2 □3 ☑4 □5 □6 □7	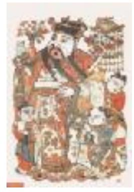<br>① □1 ☑2 □3 □4 □5 □6 □7<br>② □1 □2 □3 □4 □5 ☑6 □7<br>③ □1 □2 □3 □4 ☑5 □6 □7<br>④ □1 □2 □3 □4 □5 ☑6 □7<br>⑤ □1 □2 □3 ☑4 □5 □6 □7	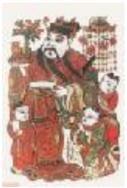<br>① ☑1 □2 □3 □4 □5 □6 □7<br>② □1 □2 □3 □4 ☑5 □6 □7<br>③ □1 □2 □3 □4 ☑5 □6 □7<br>④ □1 □2 ☑3 □4 □5 □6 □7<br>⑤ □1 □2 □3 □4 □5 ☑6 □7

	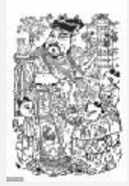	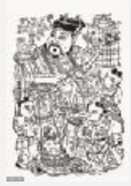	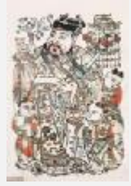	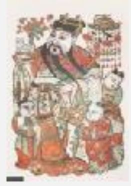	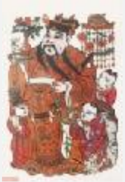
CFG=8	① □1 □2 □3 □4 □5 □6 □7<br>② □1 □2 □3 □4 □5 □6 □7<br>③ □1 □2 □3 □4 □5 □6 □7<br>④ □1 □2 □3 □4 □5 □6 □7<br>⑤ □1 □2 □3 □4 □5 □6 □7	① □1 □2 □3 □4 □5 □6 □7<br>② □1 □2 □3 □4 □5 □6 □7<br>③ □1 □2 □3 □4 □5 □6 □7<br>④ □1 □2 □3 □4 □5 □6 □7<br>⑤ □1 □2 □3 □4 □5 □6 □7	① □1 □2 □3 □4 □5 □6 □7<br>② □1 □2 □3 □4 □5 □6 □7<br>③ □1 □2 □3 □4 □5 □6 □7<br>④ □1 □2 □3 □4 □5 □6 □7<br>⑤ □1 □2 □3 □4 □5 □6 □7	① □1 □2 □3 □4 □5 □6 □7<br>② □1 □2 □3 □4 □5 □6 □7<br>③ □1 □2 □3 □4 □5 □6 □7<br>④ □1 □2 □3 □4 □5 □6 □7<br>⑤ □1 □2 □3 □4 □5 □6 □7	① □1 □2 □3 □4 □5 □6 □7<br>② □1 □2 □3 □4 □5 □6 □7<br>③ □1 □2 □3 □4 □5 □6 □7<br>④ □1 □2 □3 □4 □5 □6 □7<br>⑤ □1 □2 □3 □4 □5 □6 □7
	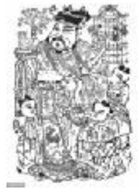	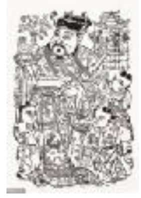	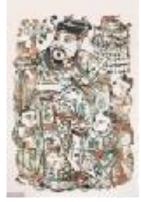	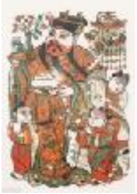	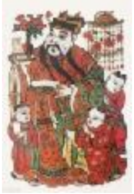
CFG=10	① □1 □2 □3 □4 □5 □6 □7<br>② □1 □2 □3 □4 □5 □6 □7<br>③ □1 □2 □3 □4 □5 □6 □7<br>④ □1 □2 □3 □4 □5 □6 □7<br>⑤ □1 □2 □3 □4 □5 □6 □7	① □1 □2 □3 □4 □5 □6 □7<br>② □1 □2 □3 □4 □5 □6 □7<br>③ □1 □2 □3 □4 □5 □6 □7<br>④ □1 □2 □3 □4 □5 □6 □7<br>⑤ □1 □2 □3 □4 □5 □6 □7	① □1 □2 □3 □4 □5 □6 □7<br>② □1 □2 □3 □4 □5 □6 □7<br>③ □1 □2 □3 □4 □5 □6 □7<br>④ □1 □2 □3 □4 □5 □6 □7<br>⑤ □1 □2 □3 □4 □5 □6 □7	① □1 □2 □3 □4 □5 □6 □7<br>② □1 □2 □3 □4 □5 □6 □7<br>③ □1 □2 □3 □4 □5 □6 □7<br>④ □1 □2 □3 □4 □5 □6 □7<br>⑤ □1 □2 □3 □4 □5 □6 □7	① □1 □2 □3 □4 □5 □6 □7<br>② □1 □2 □3 □4 □5 □6 □7<br>③ □1 □2 □3 □4 □5 □6 □7<br>④ □1 □2 □3 □4 □5 □6 □7<br>⑤ □1 □2 □3 □4 □5 □6 □7

Expert 4

Redraw amplitude<br>CFG	Redraw amplitude 0	Redraw amplitude 0.3	Redraw amplitude 0.5	Redraw amplitude 0.7	Redraw amplitude 1.0
CFG=2	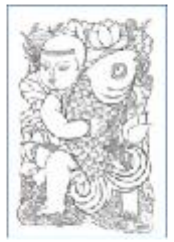	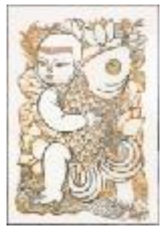	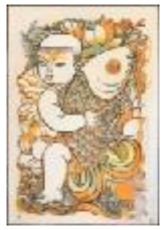	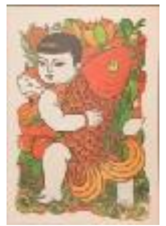	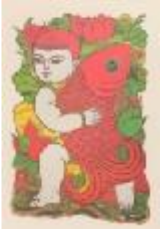
	① □1 □2 □3 □4 □5 □6 □7	① □1 □2 □3 □4 □5 □6 □7	① □1 □2 □3 □4 □5 □6 □7	① □1 □2 □3 □4 □5 □6 □7	① □1 □2 □3 □4 □5 □6 □7
	② □1 □2 □3 □4 □5 □6 □7	② □1 □2 □3 □4 □5 □6 □7	② □1 □2 □3 □4 □5 □6 □7	② □1 □2 □3 □4 □5 □6 □7	② □1 □2 □3 □4 □5 □6 □7
	③ □1 □2 □3 □4 □5 □6 □7	③ □1 □2 □3 □4 □5 □6 □7	③ □1 □2 □3 □4 □5 □6 □7	③ □1 □2 □3 □4 □5 □6 □7	③ □1 □2 □3 □4 □5 □6 □7
	④ □1 □2 □3 □4 □5 □6 □7	④ □1 □2 □3 □4 □5 □6 □7	④ □1 □2 □3 □4 □5 □6 □7	④ □1 □2 □3 □4 □5 □6 □7	④ □1 □2 □3 □4 □5 □6 □7
	⑤ □1 □2 □3 □4 □5 □6 □7	⑤ □1 □2 □3 □4 □5 □6 □7	⑤ □1 □2 □3 □4 □5 □6 □7	⑤ □1 □2 □3 □4 □5 □6 □7	⑤ □1 □2 □3 □4 □5 □6 □7
CFG=5	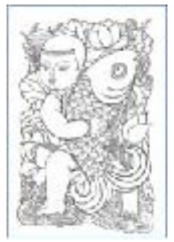	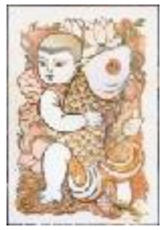	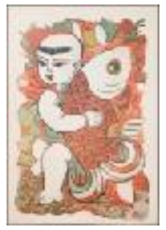	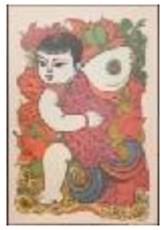	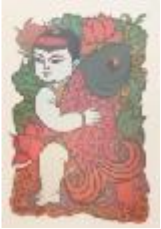
	① □1 □2 □3 □4 □5 □6 □7	① □1 □2 □3 □4 □5 □6 □7	① □1 □2 □3 □4 □5 □6 □7	① □1 □2 □3 □4 □5 □6 □7	① □1 □2 □3 □4 □5 □6 □7
	② □1 □2 □3 □4 □5 □6 □7	② □1 □2 □3 □4 □5 □6 □7	② □1 □2 □3 □4 □5 □6 □7	② □1 □2 □3 □4 □5 □6 □7	② □1 □2 □3 □4 □5 □6 □7
	③ □1 □2 □3 □4 □5 □6 □7	③ □1 □2 □3 □4 □5 □6 □7	③ □1 □2 □3 □4 □5 □6 □7	③ □1 □2 □3 □4 □5 □6 □7	③ □1 □2 □3 □4 □5 □6 □7
	④ □1 □2 □3 □4 □5 □6 □7	④ □1 □2 □3 □4 □5 □6 □7	④ □1 □2 □3 □4 □5 □6 □7	④ □1 □2 □3 □4 □5 □6 □7	④ □1 □2 □3 □4 □5 □6 □7
	⑤ □1 □2 □3 □4 □5 □6 □7	⑤ □1 □2 □3 □4 □5 □6 □7	⑤ □1 □2 □3 □4 □5 □6 □7	⑤ □1 □2 □3 □4 □5 □6 □7	⑤ □1 □2 □3 □4 □5 □6 □7
CFG=6.5	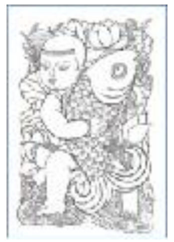	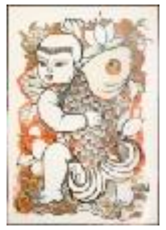	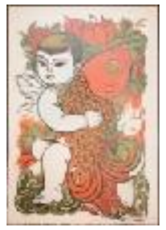	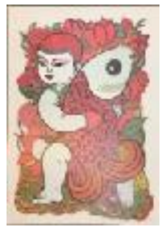	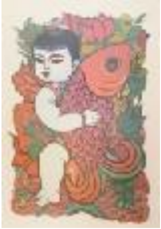
	① □1 □2 □3 □4 □5 □6 □7	① □1 □2 □3 □4 □5 □6 □7	① □1 □2 □3 □4 □5 □6 □7	① □1 □2 □3 □4 □5 □6 □7	① □1 □2 □3 □4 □5 □6 □7
	② □1 □2 □3 □4 □5 □6 □7	② □1 □2 □3 □4 □5 □6 □7	② □1 □2 □3 □4 □5 □6 □7	② □1 □2 □3 □4 □5 □6 □7	② □1 □2 □3 □4 □5 □6 □7
	③ □1 □2 □3 □4 □5 □6 □7	③ □1 □2 □3 □4 □5 □6 □7	③ □1 □2 □3 □4 □5 □6 □7	③ □1 □2 □3 □4 □5 □6 □7	③ □1 □2 □3 □4 □5 □6 □7
	④ □1 □2 □3 □4 □5 □6 □7	④ □1 □2 □3 □4 □5 □6 □7	④ □1 □2 □3 □4 □5 □6 □7	④ □1 □2 □3 □4 □5 □6 □7	④ □1 □2 □3 □4 □5 □6 □7
	⑤ □1 □2 □3 □4 □5 □6 □7	⑤ □1 □2 □3 □4 □5 □6 □7	⑤ □1 □2 □3 □4 □5 □6 □7	⑤ □1 □2 □3 □4 □5 □6 □7	⑤ □1 □2 □3 □4 □5 □6 □7

	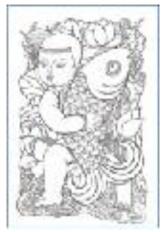	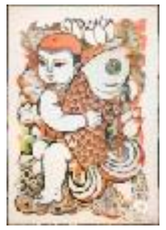	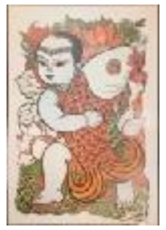	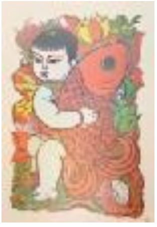	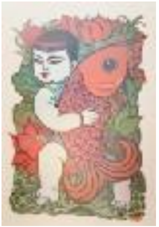
CFG=8	① □1 □2 □3 □4 □5 □6 □7	① □1 □2 □3 □4 □5 □6 □7	① □1 □2 □3 □4 □5 □6 □7	① □1 □2 □3 □4 □5 □6 □7	① □1 □2 □3 □4 □5 □6 □7
	② □1 □2 □3 □4 □5 □6 □7	② □1 □2 □3 □4 □5 □6 □7	② □1 □2 □3 □4 □5 □6 □7	② □1 □2 □3 □4 □5 □6 □7	② □1 □2 □3 □4 □5 □6 □7
	③ □1 □2 □3 □4 □5 □6 □7	③ □1 □2 □3 □4 □5 □6 □7	③ □1 □2 □3 □4 □5 □6 □7	③ □1 □2 □3 □4 □5 □6 □7	③ □1 □2 □3 □4 □5 □6 □7
	④ □1 □2 □3 □4 □5 □6 □7	④ □1 □2 □3 □4 □5 □6 □7	④ □1 □2 □3 □4 □5 □6 □7	④ □1 □2 □3 □4 □5 □6 □7	④ □1 □2 □3 □4 □5 □6 □7
	⑤ □1 □2 □3 □4 □5 □6 □7	⑤ □1 □2 □3 □4 □5 □6 □7	⑤ □1 □2 □3 □4 □5 □6 □7	⑤ □1 □2 □3 □4 □5 □6 □7	⑤ □1 □2 □3 □4 □5 □6 □7
	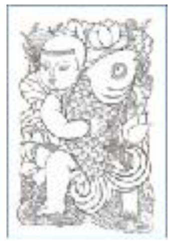	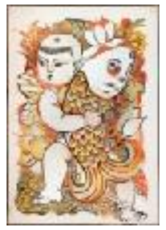	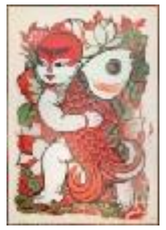	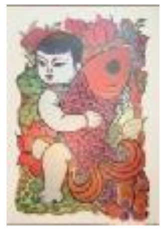	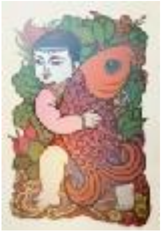
CFG=10	① □1 □2 □3 □4 □5 □6 □7	① □1 □2 □3 □4 □5 □6 □7	① □1 □2 □3 □4 □5 □6 □7	① □1 □2 □3 □4 □5 □6 □7	① □1 □2 □3 □4 □5 □6 □7
	② □1 □2 □3 □4 □5 □6 □7	② □1 □2 □3 □4 □5 □6 □7	② □1 □2 □3 □4 □5 □6 □7	② □1 □2 □3 □4 □5 □6 □7	② □1 □2 □3 □4 □5 □6 □7
	③ □1 □2 □3 □4 □5 □6 □7	③ □1 □2 □3 □4 □5 □6 □7	③ □1 □2 □3 □4 □5 □6 □7	③ □1 □2 □3 □4 □5 □6 □7	③ □1 □2 □3 □4 □5 □6 □7
	④ □1 □2 □3 □4 □5 □6 □7	④ □1 □2 □3 □4 □5 □6 □7	④ □1 □2 □3 □4 □5 □6 □7	④ □1 □2 □3 □4 □5 □6 □7	④ □1 □2 □3 □4 □5 □6 □7
	⑤ □1 □2 □3 □4 □5 □6 □7	⑤ □1 □2 □3 □4 □5 □6 □7	⑤ □1 □2 □3 □4 □5 □6 □7	⑤ □1 □2 □3 □4 □5 □6 □7	⑤ □1 □2 □3 □4 □5 □6 □7

Expert 5

Redraw amplitude / CFG	Redraw amplitude 0	Redraw amplitude 0.3	Redraw amplitude 0.5	Redraw amplitude 0.7	Redraw amplitude 1.0
CFG=2	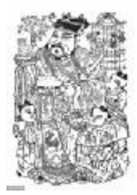<br>① □1 □2 □3 □4 □5 □6 □7<br>② □1 □2 □3 □4 □5 □6 □7<br>③ □1 □2 □3 □4 □5 □6 □7<br>④ □1 □2 □3 □4 □5 □6 □7<br>⑤ □1 □2 □3 □4 □5 □6 □7	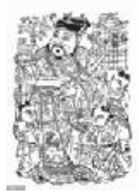<br>① □1 □2 □3 □4 □5 □6 □7<br>② □1 □2 □3 □4 □5 □6 □7<br>③ □1 □2 □3 □4 □5 □6 □7<br>④ □1 □2 □3 □4 □5 □6 □7<br>⑤ □1 □2 □3 □4 □5 □6 □7	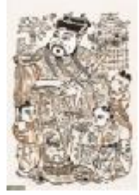<br>① □1 □2 □3 □4 □5 □6 □7<br>② □1 □2 □3 □4 □5 □6 □7<br>③ □1 □2 □3 □4 □5 □6 □7<br>④ □1 □2 □3 □4 □5 □6 □7<br>⑤ □1 □2 □3 □4 □5 □6 □7	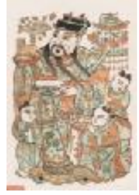<br>① □1 □2 □3 □4 □5 □6 □7<br>② □1 □2 □3 □4 □5 □6 □7<br>③ □1 □2 □3 □4 □5 □6 □7<br>④ □1 □2 □3 □4 □5 □6 □7<br>⑤ □1 □2 □3 □4 □5 □6 □7	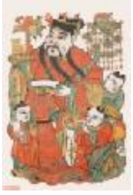<br>① □1 □2 □3 □4 □5 □6 □7<br>② □1 □2 □3 □4 □5 □6 □7<br>③ □1 □2 □3 □4 □5 □6 □7<br>④ □1 □2 □3 □4 □5 □6 □7<br>⑤ □1 □2 □3 □4 □5 □6 □7
CFG=5	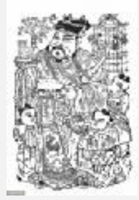<br>① □1 □2 □3 □4 □5 □6 □7<br>② □1 □2 □3 □4 □5 □6 □7<br>③ □1 □2 □3 □4 □5 □6 □7<br>④ □1 □2 □3 □4 □5 □6 □7<br>⑤ □1 □2 □3 □4 □5 □6 □7	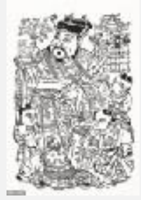<br>① □1 □2 □3 □4 □5 □6 □7<br>② □1 □2 □3 □4 □5 □6 □7<br>③ □1 □2 □3 □4 □5 □6 □7<br>④ □1 □2 □3 □4 □5 □6 □7<br>⑤ □1 □2 □3 □4 □5 □6 □7	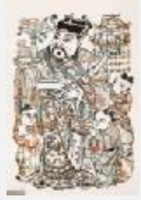<br>① □1 □2 □3 □4 □5 □6 □7<br>② □1 □2 □3 □4 □5 □6 □7<br>③ □1 □2 □3 □4 □5 □6 □7<br>④ □1 □2 □3 □4 □5 □6 □7<br>⑤ □1 □2 □3 □4 □5 □6 □7	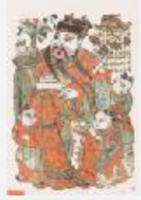<br>① □1 □2 □3 □4 □5 □6 □7<br>② □1 □2 □3 □4 □5 □6 □7<br>③ □1 □2 □3 □4 □5 □6 □7<br>④ □1 □2 □3 □4 □5 □6 □7<br>⑤ □1 □2 □3 □4 □5 □6 □7	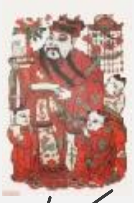<br>① □1 □2 □3 □4 □5 □6 □7<br>② □1 □2 □3 □4 □5 □6 □7<br>③ □1 □2 □3 □4 □5 □6 □7<br>④ □1 □2 □3 □4 □5 □6 □7<br>⑤ □1 □2 □3 □4 □5 □6 □7
CFG=6.5	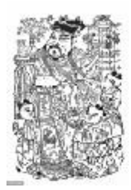<br>① □1 □2 □3 □4 □5 □6 □7<br>② □1 □2 □3 □4 □5 □6 □7<br>③ □1 □2 □3 □4 □5 □6 □7<br>④ □1 □2 □3 □4 □5 □6 □7<br>⑤ □1 □2 □3 □4 □5 □6 □7	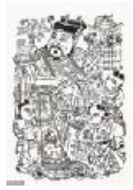<br>① □1 □2 □3 □4 □5 □6 □7<br>② □1 □2 □3 □4 □5 □6 □7<br>③ □1 □2 □3 □4 □5 □6 □7<br>④ □1 □2 □3 □4 □5 □6 □7<br>⑤ □1 □2 □3 □4 □5 □6 □7	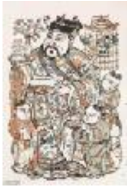<br>① □1 □2 □3 □4 □5 □6 □7<br>② □1 □2 □3 □4 □5 □6 □7<br>③ □1 □2 □3 □4 □5 □6 □7<br>④ □1 □2 □3 □4 □5 □6 □7<br>⑤ □1 □2 □3 □4 □5 □6 □7	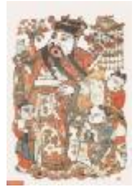<br>① □1 □2 □3 □4 □5 □6 □7<br>② □1 □2 □3 □4 □5 □6 □7<br>③ □1 □2 □3 □4 □5 □6 □7<br>④ □1 □2 □3 □4 □5 □6 □7<br>⑤ □1 □2 □3 □4 □5 □6 □7	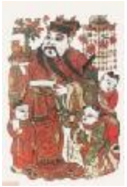<br>① □1 □2 □3 □4 □5 □6 □7<br>② □1 □2 □3 □4 □5 □6 □7<br>③ □1 □2 □3 □4 □5 □6 □7<br>④ □1 □2 □3 □4 □5 □6 □7<br>⑤ □1 □2 □3 □4 □5 □6 □7

	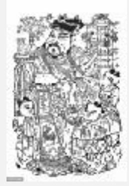	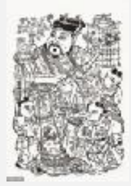	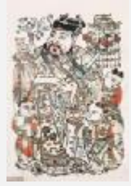	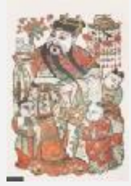	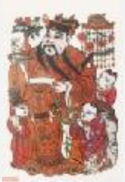
CFG=8	① □1 □2 □3 □4 □5 □6 □7<br>② □1 □2 □3 □4 □5 □6 □7<br>③ □1 □2 □3 □4 □5 □6 □7<br>④ □1 □2 □3 □4 □5 □6 □7<br>⑤ □1 □2 □3 □4 □5 □6 □7	① □1 □2 □3 □4 □5 □6 □7<br>② □1 □2 □3 □4 □5 □6 □7<br>③ □1 □2 □3 □4 □5 □6 □7<br>④ □1 □2 □3 □4 □5 □6 □7<br>⑤ □1 □2 □3 □4 □5 □6 □7	① □1 □2 □3 □4 □5 □6 □7<br>② □1 □2 □3 □4 □5 □6 □7<br>③ □1 □2 □3 □4 □5 □6 □7<br>④ □1 □2 □3 □4 □5 □6 □7<br>⑤ □1 □2 □3 □4 □5 □6 □7	① □1 □2 □3 □4 □5 □6 □7<br>② □1 □2 □3 □4 □5 □6 □7<br>③ □1 □2 □3 □4 □5 □6 □7<br>④ □1 □2 □3 □4 □5 □6 □7<br>⑤ □1 □2 □3 □4 □5 □6 □7	① □1 □2 □3 □4 □5 □6 □7<br>② □1 □2 □3 □4 □5 □6 □7<br>③ □1 □2 □3 □4 □5 □6 □7<br>④ □1 □2 □3 □4 □5 □6 □7<br>⑤ □1 □2 □3 □4 □5 □6 □7
	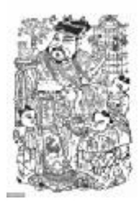	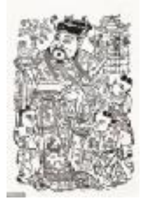	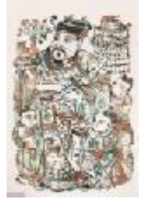	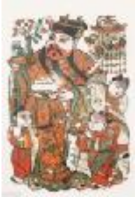	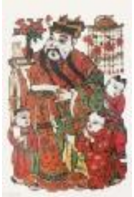
CFG=10	① □1 □2 □3 □4 □5 □6 □7<br>② □1 □2 □3 □4 □5 □6 □7<br>③ □1 □2 □3 □4 □5 □6 □7<br>④ □1 □2 □3 □4 □5 □6 □7<br>⑤ □1 □2 □3 □4 □5 □6 □7	① □1 □2 □3 □4 □5 □6 □7<br>② □1 □2 □3 □4 □5 □6 □7<br>③ □1 □2 □3 □4 □5 □6 □7<br>④ □1 □2 □3 □4 □5 □6 □7<br>⑤ □1 □2 □3 □4 □5 □6 □7	① □1 □2 □3 □4 □5 □6 □7<br>② □1 □2 □3 □4 □5 □6 □7<br>③ □1 □2 □3 □4 □5 □6 □7<br>④ □1 □2 □3 □4 □5 □6 □7<br>⑤ □1 □2 □3 □4 □5 □6 □7	① □1 □2 □3 □4 □5 □6 □7<br>② □1 □2 □3 □4 □5 □6 □7<br>③ □1 □2 □3 □4 □5 □6 □7<br>④ □1 □2 □3 □4 □5 □6 □7<br>⑤ □1 □2 □3 □4 □5 □6 □7	① □1 □2 □3 □4 □5 □6 □7<br>② □1 □2 □3 □4 □5 □6 □7<br>③ □1 □2 □3 □4 □5 □6 □7<br>④ □1 □2 □3 □4 □5 □6 □7<br>⑤ □1 □2 □3 □4 □5 □6 □7

Expert 5

CFG \ Redraw amplitude	Redraw amplitude 0	Redraw amplitude 0.3	Redraw amplitude 0.5	Redraw amplitude 0.7	Redraw amplitude 1.0
CFG=2	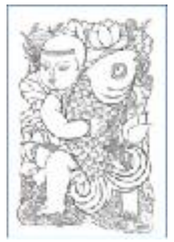<br>① □1 □2 □3 □4 □5 □6 □7<br>② □1 □2 □3 □4 □5 □6 □7<br>③ □1 □2 □3 □4 □5 □6 □7<br>④ □1 □2 □3 □4 □5 □6 □7<br>⑤ □1 □2 □3 □4 □5 □6 □7	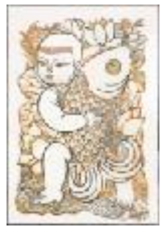<br>① □1 □2 □3 □4 □5 □6 □7<br>② □1 □2 □3 □4 □5 □6 □7<br>③ □1 □2 □3 □4 □5 □6 □7<br>④ □1 □2 □3 □4 □5 □6 □7<br>⑤ □1 □2 □3 □4 □5 □6 □7	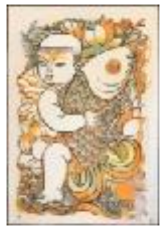<br>① □1 □2 □3 □4 □5 □6 □7<br>② □1 □2 □3 □4 □5 □6 □7<br>③ □1 □2 □3 □4 □5 □6 □7<br>④ □1 □2 □3 □4 □5 □6 □7<br>⑤ □1 □2 □3 □4 □5 □6 □7	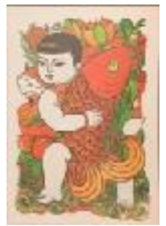<br>① □1 □2 □3 □4 □5 □6 □7<br>② □1 □2 □3 □4 □5 □6 □7<br>③ □1 □2 □3 □4 □5 □6 □7<br>④ □1 □2 □3 □4 □5 □6 □7<br>⑤ □1 □2 □3 □4 □5 □6 □7	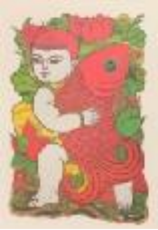<br>① □1 □2 □3 □4 □5 □6 □7<br>② □1 □2 □3 □4 □5 □6 □7<br>③ □1 □2 □3 □4 □5 □6 □7<br>④ □1 □2 □3 □4 □5 □6 □7<br>⑤ □1 □2 □3 □4 □5 □6 □7
CFG=5	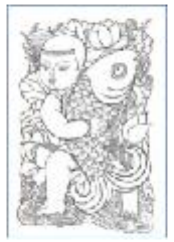<br>① □1 □2 □3 □4 □5 □6 □7<br>② □1 □2 □3 □4 □5 □6 □7<br>③ □1 □2 □3 □4 □5 □6 □7<br>④ □1 □2 □3 □4 □5 □6 □7<br>⑤ □1 □2 □3 □4 □5 □6 □7	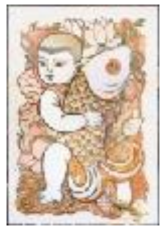<br>① □1 □2 □3 □4 □5 □6 □7<br>② □1 □2 □3 □4 □5 □6 □7<br>③ □1 □2 □3 □4 □5 □6 □7<br>④ □1 □2 □3 □4 □5 □6 □7<br>⑤ □1 □2 □3 □4 □5 □6 □7	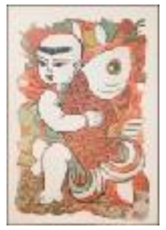<br>① □1 □2 □3 □4 □5 □6 □7<br>② □1 □2 □3 □4 □5 □6 □7<br>③ □1 □2 □3 □4 □5 □6 □7<br>④ □1 □2 □3 □4 □5 □6 □7<br>⑤ □1 □2 □3 □4 □5 □6 □7	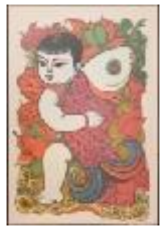<br>① □1 □2 □3 □4 □5 □6 □7<br>② □1 □2 □3 □4 □5 □6 □7<br>③ □1 □2 □3 □4 □5 □6 □7<br>④ □1 □2 □3 □4 □5 □6 □7<br>⑤ □1 □2 □3 □4 □5 □6 □7	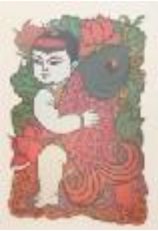<br>① □1 □2 □3 □4 □5 □6 □7<br>② □1 □2 □3 □4 □5 □6 □7<br>③ □1 □2 □3 □4 □5 □6 □7<br>④ □1 □2 □3 □4 □5 □6 □7<br>⑤ □1 □2 □3 □4 □5 □6 □7
CFG=6.5	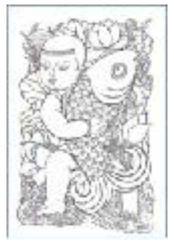<br>① □1 □2 □3 □4 □5 □6 □7<br>② □1 □2 □3 □4 □5 □6 □7<br>③ □1 □2 □3 □4 □5 □6 □7<br>④ □1 □2 □3 □4 □5 □6 □7<br>⑤ □1 □2 □3 □4 □5 □6 □7	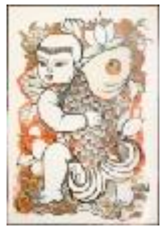<br>① □1 □2 □3 □4 □5 □6 □7<br>② □1 □2 □3 □4 □5 □6 □7<br>③ □1 □2 □3 □4 □5 □6 □7<br>④ □1 □2 □3 □4 □5 □6 □7<br>⑤ □1 □2 □3 □4 □5 □6 □7	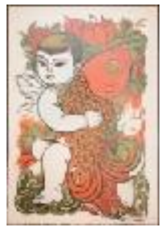<br>① □1 □2 □3 □4 □5 □6 □7<br>② □1 □2 □3 □4 □5 □6 □7<br>③ □1 □2 □3 □4 □5 □6 □7<br>④ □1 □2 □3 □4 □5 □6 □7<br>⑤ □1 □2 □3 □4 □5 □6 □7	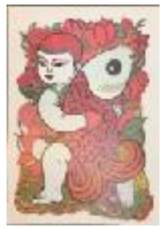<br>① □1 □2 □3 □4 □5 □6 □7<br>② □1 □2 □3 □4 □5 □6 □7<br>③ □1 □2 □3 □4 □5 □6 □7<br>④ □1 □2 □3 □4 □5 □6 □7<br>⑤ □1 □2 □3 □4 □5 □6 □7	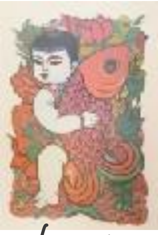<br>① □1 □2 □3 □4 □5 □6 □7<br>② □1 □2 □3 □4 □5 □6 □7<br>③ □1 □2 □3 □4 □5 □6 □7<br>④ □1 □2 □3 □4 □5 □6 □7<br>⑤ □1 □2 □3 □4 □5 □6 □7

CFG=8

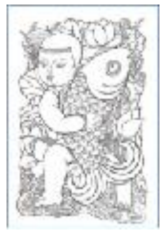	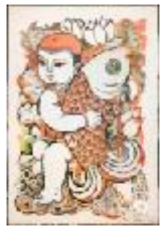	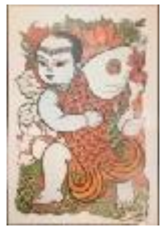	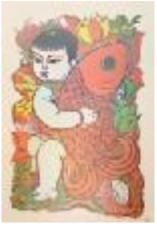	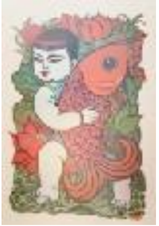
① □1 □2 □3 □4 □5 □6 □7	① □1 □2 □3 □4 □5 □6 □7	① □1 □2 □3 □4 □5 □6 □7	① □1 □2 □3 □4 □5 □6 □7	① □1 □2 □3 □4 □5 □6 □7
② □1 □2 □3 □4 □5 □6 □7	② □1 □2 □3 □4 □5 □6 □7	② □1 □2 □3 □4 □5 □6 □7	② □1 □2 □3 □4 □5 □6 □7	② □1 □2 □3 □4 □5 □6 □7
③ □1 □2 □3 □4 □5 □6 □7	③ □1 □2 □3 □4 □5 □6 □7	③ □1 □2 □3 □4 □5 □6 □7	③ □1 □2 □3 □4 □5 □6 □7	③ □1 □2 □3 □4 □5 □6 □7
④ □1 □2 □3 □4 □5 □6 □7	④ □1 □2 □3 □4 □5 □6 □7	④ □1 □2 □3 □4 □5 □6 □7	④ □1 □2 □3 □4 □5 □6 □7	④ □1 □2 □3 □4 □5 □6 □7
⑤ □1 □2 □3 □4 □5 □6 □7	⑤ □1 □2 □3 □4 □5 □6 □7	⑤ □1 □2 □3 □4 □5 □6 □7	⑤ □1 □2 □3 □4 □5 □6 □7	⑤ □1 □2 □3 □4 □5 □6 □7

CFG=10

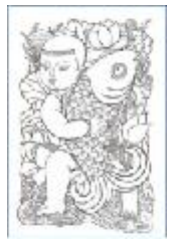	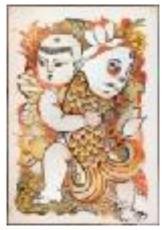	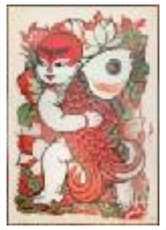	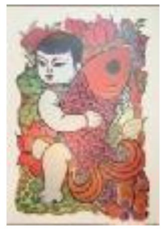	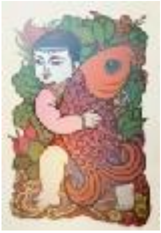
① □1 □2 □3 □4 □5 □6 □7	① □1 □2 □3 □4 □5 □6 □7	① □1 □2 □3 □4 □5 □6 □7	① □1 □2 □3 □4 □5 □6 □7	① □1 □2 □3 □4 □5 □6 □7
② □1 □2 □3 □4 □5 □6 □7	② □1 □2 □3 □4 □5 □6 □7	② □1 □2 □3 □4 □5 □6 □7	② □1 □2 □3 □4 □5 □6 □7	② □1 □2 □3 □4 □5 □6 □7
③ □1 □2 □3 □4 □5 □6 □7	③ □1 □2 □3 □4 □5 □6 □7	③ □1 □2 □3 □4 □5 □6 □7	③ □1 □2 □3 □4 □5 □6 □7	③ □1 □2 □3 □4 □5 □6 □7
④ □1 □2 □3 □4 □5 □6 □7	④ □1 □2 □3 □4 □5 □6 □7	④ □1 □2 □3 □4 □5 □6 □7	④ □1 □2 □3 □4 □5 □6 □7	④ □1 □2 □3 □4 □5 □6 □7
⑤ □1 □2 □3 □4 □5 □6 □7	⑤ □1 □2 □3 □4 □5 □6 □7	⑤ □1 □2 □3 □4 □5 □6 □7	⑤ □1 □2 □3 □4 □5 □6 □7	⑤ □1 □2 □3 □4 □5 □6 □7
